# Troponin: biology, molecular mechanisms, and multidimensional clinical applications

**DOI:** 10.1186/s43556-026-00597-z

**Published:** 2026-09-28

**Authors:** Michaela Adamcova, Lukáš Petráň

**Affiliations:** Charles University, Faculty of Medicine in Hradec Kralove, Department of Physiology, Zborovská 2207, Hradec Kralove, 500 03 Czech Republic

**Keywords:** Troponin, Cancer, Transcriptional regulation, Epigenetics, Mitochondrial metabolism, Nuclear signaling

## Abstract

Troponin is classically recognized as the central regulator of striated muscle contraction. However, emerging evidence indicates that troponin subunits are also expressed in numerous non-muscle cell types, where they perform diverse non-classical functions independent of contractile regulation. Recent studies have implicated troponin in transcriptional regulation, epigenetic remodeling, mitochondrial bioenergetics, and calcium signaling. Troponin has also been shown to inhibit angiogenesis and is present in extracellular vesicles. Together, these findings suggest that troponin participates in fundamental cellular processes that extend far beyond muscle physiology. Despite these advances, the molecular mechanisms underlying these non-classical functions remain incompletely understood, and the relationship between troponin's structural biology and its roles in both cancerous and non-cancerous cells has been only partially explored. In this review, we integrate current experimental evidence with recent bioinformatic analyses to provide a comprehensive overview of troponin biology beyond the sarcomere. Particular emphasis is placed on the molecular mechanisms governing nuclear localization, protein–protein interactions, transcriptional and epigenetic regulation, and mitochondrial function, as well as on the distinct biological roles of individual troponin isoforms. We further discuss the growing evidence linking aberrant troponin expression and function to cancer biology, highlighting its context- and isoform-dependent activities that may either promote or suppress tumor development. Collectively, these findings redefine troponin as a multifunctional regulatory protein and identify it as a promising subject for future research.

## Introduction

Troponin is a highly conserved regulatory protein complex that plays a central role in the Ca^2+^-dependent regulation of striated muscle contraction and has evolved from a fundamental component of muscle physiology into one of the most important molecular biomarkers of myocardial injury [1–4]. The history of troponin research dates to the early 1960 s, when Setsuro Ebashi demonstrated that a previously unrecognized myofibrillar protein was involved in the Ca^2+^-dependent regulation of muscle contraction [1, 2]. In 1963, Ebashi reported this protein as the “third component” involved in the Ca^2+^-dependent regulation of actomyosin, establishing the foundation for the subsequent identification and characterization of troponin [1]. Biochemical studies, including important contributions from S. V. Perry and colleagues, established that troponin is a multiprotein complex composed of three functionally distinct subunits: troponin C (TnC), which binds Ca^2+^; troponin I (TnI), which inhibits actomyosin ATPase activity; and troponin T (TnT), which mediates the interaction of the complex with tropomyosin [3–5]. Structural and biochemical studies have subsequently provided detailed insights into the molecular organization of the troponin complex and its Ca^2+^-dependent conformational regulation [4, 5]. Importantly, the three troponin subunits differ substantially in their tissue specificity. Unlike TnI and TnT, TnC is not sufficiently cardiac-specific for use as a circulating biomarker of myocardial injury [6].

The transition of troponin from a component of the contractile apparatus to a clinically established biomarker was enabled by the development of immunoassays targeting cardiac-specific troponin isoforms. In 1989, Katus and colleagues reported an enzyme-linked immunoassay for cTnT and demonstrated its potential for detecting acute myocardial infarction [7]. In 1992, Katus and colleagues described a new monoclonal antibody-based immunoassay for cTnT [8], while Bodor and colleagues independently developed a double-monoclonal sandwich immunoassay for cTnI [9]. During the 1990s, accumulating clinical evidence demonstrated the superior cardiac specificity and diagnostic performance of cTnI and cTnT compared with conventional biomarkers such as creatine kinase-MB and myoglobin, leading to their incorporation into the diagnostic framework for acute myocardial infarction [10–12]. The subsequent introduction of high-sensitivity cardiac troponin (hs-cTn) assays, which provide improved analytical sensitivity and precision, enabled the detection of substantially lower circulating concentrations of cTn and facilitated earlier diagnosis and risk stratification in patients with suspected acute coronary syndromes [13–16].

Beyond acute coronary syndromes, cardiac troponins have become increasingly important in cardio-oncology, where cTnI and cTnT are used as biomarkers of myocardial injury associated with potentially cardiotoxic anticancer therapies [17–25]. Meta-analyses have demonstrated that increases in cardiac troponin during cancer therapy are associated with an increased risk of subsequent left ventricular dysfunction, supporting their potential role in the early identification of patients at increased risk of cancer therapy-related cardiac dysfunction [26]. Current European cardio-oncology recommendations incorporate cardiac troponin, together with natriuretic peptides and cardiac imaging, into cardiovascular risk assessment and surveillance for selected anticancer therapies [27].

Importantly, an emerging and largely unresolved area of troponin research concerns the possibility that troponin-related proteins may have biological functions extending beyond their established role in the sarcomeric contractile apparatus. These observations have generated hypotheses concerning possible roles of troponin in cellular signaling, transcriptional regulation, angiogenesis, mitochondrial function, and cancer biology [28–31]. The potential involvement of troponin-related proteins in cancer biology represents an even more preliminary but potentially important area of investigation. Several troponin genes have been detected in non-muscle tissues and have been associated with cancer-related molecular phenotypes [32–36]. However, it remains unclear whether altered expression of troponin-related genes in tumors reflects direct tumor-intrinsic mechanisms, changes in cellular differentiation, stromal or tissue remodeling, or other consequences of cancer-associated pathology. This emerging field therefore warrants critical evaluation and provides an additional rationale for a comprehensive molecular review of troponin.

In this review, we aim to provide an integrated overview of troponin biology, connecting its fundamental molecular functions with its pathological, diagnostic, and emerging translational significance. We first summarize the genetics and structure of troponin and describe the isoforms and physiological functions of TnC, TnI, and TnT. We then discuss tissue-specific expression, developmental regulation, and post-translational modifications of troponin and examine how alterations in these processes affect sarcomeric function. Next, we focus on emerging non-classical aspects of troponin biology. Recent studies have implicated troponin in transcriptional regulation, epigenetic remodeling, mitochondrial bioenergetics, and calcium signaling. Troponin has also been shown to inhibit angiogenesis and to be present in extracellular vesicles. Finally, we specifically address the emerging and unresolved question of whether troponin may participate directly in cancer biology and tumor pathogenesis, critically distinguishing established experimental evidence from preliminary observations and hypotheses. We integrate current experimental evidence with bioinformatic analyses to provide a coherent framework for understanding the expanding biological and translational significance of troponin and to identify priorities for future research.

## Molecular biology, biochemistry, and functional regulation of troponin

The troponin complex is a highly conserved regulatory protein complex that plays a central role in excitation–contraction coupling in skeletal and cardiac muscle. It translates changes in intracellular Ca^2+^ concentration into the regulation of actin–myosin interaction through Ca^2+^-dependent conformational changes within the complex. The troponin complex consists of three subunits: troponin T (TnT), troponin I (TnI), and troponin C (TnC). Each subunit exists in multiple tissue-specific isoforms encoded by distinct genes. TnT binds to tropomyosin and anchors the complex within the thin filament. TnI interacts with actin and, together with tropomyosin, inhibits actin–myosin interaction under resting, low-Ca^2+^ conditions. TnC serves as the Ca^2+^-sensing regulatory subunit; upon Ca^2+^ binding, it undergoes conformational changes that alter the interactions and positioning of the other troponin subunits, ultimately allowing actin–myosin interaction and muscle contraction [37].

Although the fundamental mechanism of troponin function is conserved across muscle types, differences in the sequences and structures of individual tissue-specific isoforms, alternative splicing, and post-translational modifications contribute to the fine-tuning of Ca^2+^ sensitivity and contractile regulation under different conditions [38–43]. Maintaining of this fine-tuning is therefore essential for maintaining appropriate Ca^2+^ responsiveness and contractile function, whereas alterations in the structure, expression, or post-translational regulation of individual troponin subunits may disrupt this balance. Such disturbances can have important pathological consequences, particularly in the heart, where altered troponin function can impair Ca^2+^-dependent regulation of contraction and contribute to the development of cardiomyopathies and other diseases [44–49].

### Troponin gene families and isoform diversity

Three genes encode the troponin T isoforms: *TNNT1* (19q13.4), encoding slow skeletal muscle troponin T (ssTnT); *TNNT2* (1q32), encoding cardiac troponin T (cTnT); and* TNNT3 *(11p15.5), encoding fast skeletal muscle troponin T (fsTnT). *TNNT1* gene is composed of 14 exons. Of those 14 exons, exon 5 undergoes alternative splicing generating 2 alternative splicing variants [39]. Unlike *TNNT1*, cardiac and fast skeletal troponin genes are subject to greater levels of alternative splicing [40]. *TNNT2* gene is composed of 17 exons. Exons 4, 5, and 13 undergo alternative splicing. Exon 5 is expressed only during embryogenesis and is not present in adult cardiac troponin T [39]. *TNNT1* is the most extensively spliced troponin T gene. From its 19 exons, exons 4, 5, 6, 7, 8, 16, 17 are spliced [40]. The troponin I isoforms are encoded by *TNNI1* (1q31.3), encoding slow skeletal muscle troponin I (ssTnI); *TNNI2* (11p15.5), encoding fast skeletal muscle troponin I (fsTnI); and *TNNI3* (19q13.4), encoding cardiac troponin I (cTnI) [42]. Unlike troponin T, troponin I was not shown to undergo alternative splicing, instead its function is regulated by other means. The post-translational modifications [41] and switching between slow skeletal and cardiac variants during embryonic development ensure its proper functionality [38]. Troponin C isoforms are encoded by two genes: *TNNC1* (3p21.1), encoding cardiac troponin C (cTnC) functioning in both cardiac and slow skeletal muscle fibers, and *TNNC2* (20q12–q13.11), encoding fast skeletal muscle troponin C [50]. Troponin C also does not undergo alternative splicing [51].

### Structure of troponin subunits

#### Molecular architecture of troponin T

Troponin T is the largest subunit of the troponin complex. It interacts with the thin filament, anchoring the remaining troponin subunits to the actin–tropomyosin filament, and also contributes to the positioning of the troponin complex relative to the actin filament [52, 53].

Structurally, troponin T is divided into three major domains: the N-terminal domain, the linker region, and the C-terminal domain. Early fragmentation studies based on chymotryptic cleavage separated troponin T into two fragments, termed T1 and T2 [53, 54]. Those fragments correspond to the N-terminal and C-terminal domains, respectively [55]. The N-terminal region begins with a hypervariable region [54]. Although this region does not contain binding sites for other sarcomeric proteins, it undergoes alternative splicing during postnatal development and under pathological conditions [40, 56, 57], thereby modulating the affinity of troponin T for tropomyosin [58]. Part of the N-terminal domain interacts with the N-terminal region of tropomyosin [59]. The remaining portion of the T1 domain extends over the N-/C-terminal overlapping domain of tropomyosin and the C-terminal region of an adjacent tropomyosin molecule [60]. The linker region connects the N-terminal T1 domain with the C-terminal T2 domain and provides structural flexibility to the molecule. This region appears to be largely intrinsically disordered [52]. The C-terminal domain of troponin T begins with a second tropomyosin binding site [59]. Structurally, it consists of two α-helices, named H1 and H2, followed by a C-terminal peptide. Helix H2 forms an antiparallel coiled-coil structure with helix H2 of troponin I and additionally interacts with helix H1 of troponin I and the C-terminal region of troponin C. The C-terminal peptide itself is unstructured [38, 61].

**Fig. 1:**
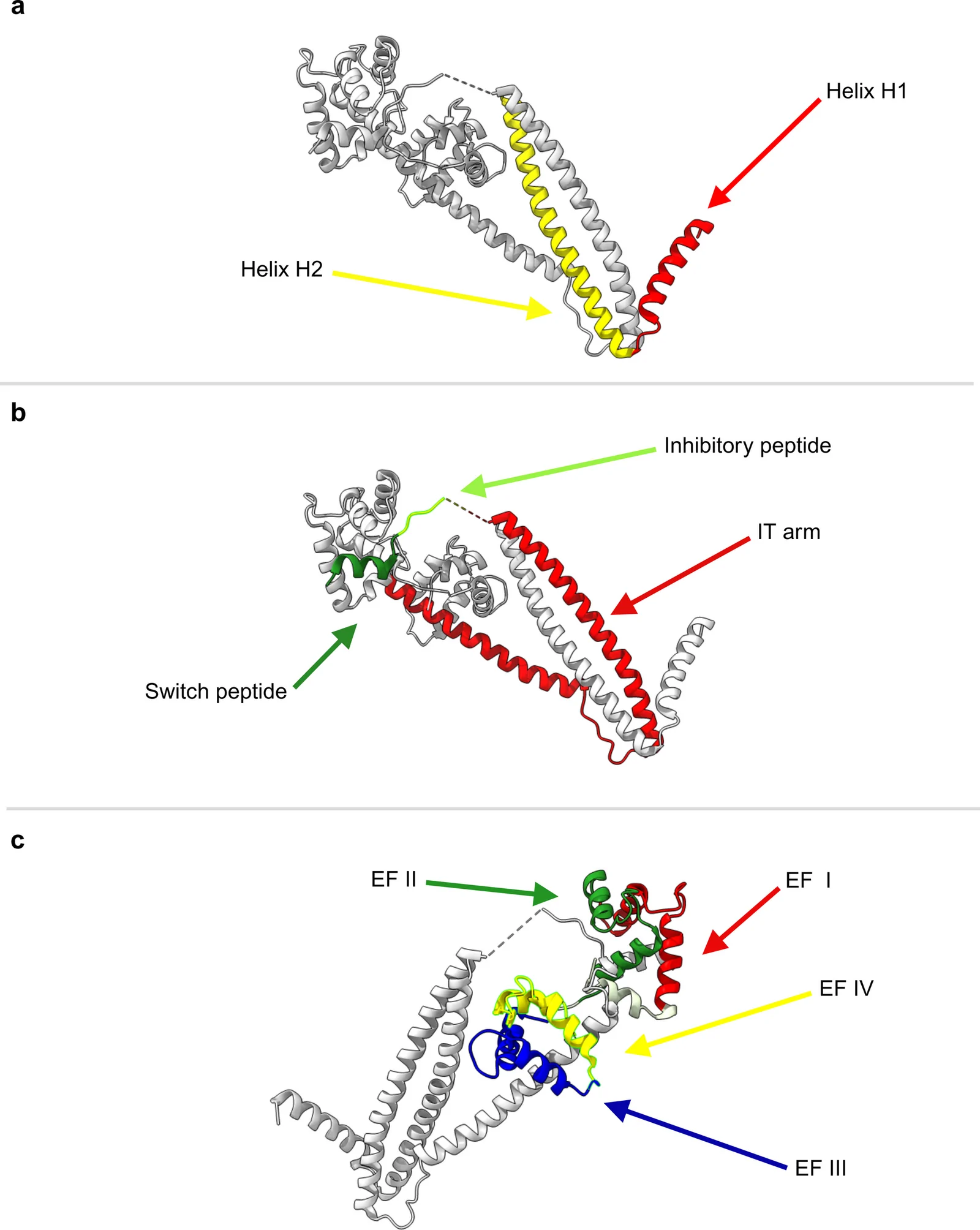
The figure shows the cardiac troponin complex. **a** Cardiac troponins I and C are displayed in light gray, while cardiac troponin T helix H1 is highlighted in red and helix H2 in yellow. Troponin T is composed of three major domains: the N-terminal domain, the linker region, and the C-terminal domain. Based on fragmentation studies, it is also divided into the T1 and T2 fragments, corresponding to the N-terminal and C-terminal domains, respectively. The N-terminal domain begins with a hypervariable region and includes a tropomyosin-binding site. The linker region connects the N-terminal and C-terminal domains. The C-terminal domain consists of helices H1 and H2, as well as the C-terminal peptide. In this model, only helices H1 and H2 are shown. Helix H2 interacts with troponins I and C. **b** Cardiac troponins T and C are displayed in light gray, while the domains of cardiac troponin I are highlighted. The IT arm domain is shown in red, the inhibitory peptide in yellow, and the switch peptide in green. Troponin I consists of six structural regions: the N-terminal cardiac-specific extension, the N-terminal region, the IT arm, the inhibitory peptide, the switch peptide, and the C-terminal region. The N-terminal cardiac-specific extension is unique to cardiac troponin I. The N-terminal region forms the H1 α-helix and interacts with troponin C. The IT arm consists of helices H1 and H2 connected by a U-turn and interacts with troponins T and C. The function of the inhibitory peptide is not fully elucidated. The switch peptide contains helix H3 and interacts with troponin C. The C-terminal region includes helix H4 and interacts with actin in the Ca^2+^-free state. **c** Cardiac troponins T and I are displayed in light gray, while the domains of cardiac troponin C are highlighted. EF-hand motif I is shown in red, EF-hand motif II in green, EF-hand motif III in blue, and EF-hand motif IV in yellow. The EF-hand motif is a helix–loop–helix structural motif capable of binding divalent ions. Troponin C consists of two globular domains connected by a linker region. Each globular domain contains two EF-hand motifs. EF-hand motifs I and II are located in the N-terminal globular domain and are highly selective for calcium ions. EF-hand motifs III and IV are located in the C-terminal globular domain and are less specific for calcium. Under physiological conditions, motifs III and IV are occupied by divalent ions. EF-hand motif II functions as the primary Ca^2+^ sensor

The structure of cardiac troponin T (TNNT2), including annotated functional domains, is shown in Fig. 1. The 3D structure of the troponin complex used in Fig. 1. was downloaded from the PDBe website [62] and edited using ChimeraX [63]. The annotation was based on the work of Marston and Zamora [52] and on the Uniprot entry for TNNC1. The Uniprot ID of the TNNC1 is P63316. The PDB ID of the complex is pdb_00001j1d. The primary publication associated with this PDB entry was published by Takeda et al. [64].

#### Molecular architecture of troponin I

Troponin I acts as an inhibitor of actomyosin interaction. Together with tropomyosin, it blocks the myosin binding sites on actin [65]. In the Ca^2+^-saturated state, troponin C interacts with troponin I and induces a conformational change that permits myosin binding [54].

Troponin I consists of six structural regions: the N-terminal cardiac-specific extension, N-terminal region, IT arm, inhibitory peptide, switch peptide, and C-terminal region. The N-terminal cardiac-specific extension is present only in cardiac troponin I (TNNI3) [55, 66]. It can undergo phosphorylation on serine residues 23 and 24, which alters the calcium sensitivity of the troponin complex [66]. Structurally, this region contains an acidic segment and an Xaa–Pro motif that can form an unstable α-helix [52]. The N-terminal region forms an H1 α-helix [55, 66] and interacts with the C-terminal domain of troponin C [55]. The IT arm consists of two α-helices (H1 and H2) connected by a U-turn. They interact with troponin I to form a rigid coiled-coil structure. This domain also anchors the C-terminal domain of troponin C [52]. The inhibitory peptide was historically thought to interact with troponin C, actin, and myosin [42, 55]; however, more recent studies have questioned this interpretation [52, 66]. The switch peptide contains helix H3 and binds to the N-terminal domain of troponin C. The C-terminal region includes helix H4 and interacts with tropomyosin in a Ca^2+^-dependent manner [42, 67, 68]. The binding of Ca^2+^ to troponin C induces a conformational change in the switch region of troponin I, which moves the C-terminal domain away from actin, thereby enabling actomyosin interaction [66, 69] The structure of cardiac troponin I (TNNI3) with annotated domains is shown in Fig. 1.

#### Molecular architecture of troponin C

Troponin C differs from the other troponin subunits by the presence of EF-hand helix–loop–helix motifs, which enable Ca^2+^ binding [55, 70, 71]. Structurally, troponin C consists of two globular domains connected by a flexible linker region [50, 52]. Each globular domain contains two EF-hand motifs, which differ in their calcium-binding properties.

EF-hand motifs I and II, located in the N-terminal globular domain, exhibit lower calcium affinity but higher selectivity for Ca^2+^. In contrast, EF-hand motifs III and IV display higher calcium affinity and are also capable of binding other divalent cations, such as Mg^2+^. Under physiological conditions, EF-hand motifs III and IV are occupied by divalent ions, whereas EF-hand motif I remains unoccupied. EF-hand motif II functions as the primary Ca^2+^ sensor [52, 71, 72]. Each globular domain contains four α-helices [50, 52, 72]. The N-terminal domain is also called the regulatory domain, because it contains the Ca^2+^-sensing EF-hand motif II. The C-terminal domain is referred to as the structural domain and mediates interactions with the rest of the troponin complex [38, 52]. The linker region differs between cardiac TNNC1 and fast skeletal TNNC2 isoforms. In TNNC1, the linker is intrinsically disordered, providing increased conformational flexibility, whereas in TNNC2 it adopts a more rigid α-helical structure [64, 73]. Upon Ca^2+^ binding, troponin C undergoes a significant conformational change that enables interaction with the troponin I switch region and the exposure of myosin-binding sites on actin [50]. The structure of cardiac troponin C (TNNC1) with annotated domains is shown in Fig. 1.

### Molecular mechanisms of Ca^2+^-dependent thin-filament regulation

The regulatory role of troponin in controlling thin filament activation in response to increases in intracellular Ca^2+^ levels is well established, with the fundamental mechanisms underlying this regulation having been extensively described [52]. More recent studies, however, have provided increasingly detailed insights into the molecular structure of the troponin complex. Structural data obtained using cryo-electron microscopy have enabled a more comprehensive characterization of the conformational changes within the troponin complex induced by Ca^2+^ binding [69, 74–76]. These advances have also provided structural insights into the mechanisms underlying the inactivation of striated muscle contraction, a previously poorly understood aspect of troponin function [69].

The basic structure of the thin filament consists of a repeating pattern of two actin strands, each composed of 14 G-actin molecules arranged in a double helix, two tropomyosin molecules that coil around the actin strands, and two troponin complexes. The two troponin complexes positioned opposite to each other are not identical in their arrangement, with one extending over one G-actin molecule farther along the filament than the other [77]. In the Ca^2+^-unsaturated state, the troponin complex extends along the actin double helix within the sarcomere. However, inhibition of the actin–myosin interaction is not mediated solely by TnI. The N-terminal region of TnT, the core domain of the troponin complex, and the C-terminal region of TnI all contribute to the inhibition of muscle contraction by sterically interfering with actin–myosin interaction. This inhibition is further reinforced by the overlap between the N-terminal region of TnT and tropomyosin [74]. Furthermore, a single troponin complex does not interact exclusively with one tropomyosin molecule but instead cross-links tropomyosin molecules associated with both actin strands [77].

When Ca^2+^ binds to the Ca^2+^-sensing EF-hand motif II, located within the N-terminal domain of TnC, it induces substantial conformational changes in the troponin complex [38, 52]. TnC adopts a more open conformation [76], exposing a hydrophobic pocket within its N-terminal region. This conformational change enables binding of the region of TnI known as the switch peptide to TnC [50, 67, 68]. Binding of the switch peptide to TnC weakens the interaction between the C-terminal domain of TnI and actin–tropomyosin [76]. Detachment of this region of TnI from actin represents one of the most prominent structural changes following Ca^2+^ binding and is a crucial step in permitting actin–myosin interaction. In response to these conformational changes within the troponin complex, tropomyosin shifts azimuthally along the actin filament, exposing the myosin-binding sites on actin. Following myosin attachment to actin, tropomyosin shifts further toward the fully open position [74, 75, 78]. This further movement may limit the interaction between the C-terminal domain of TnI and tropomyosin, thereby promoting stabilization of the open state of the thin filament regulatory system [76].

Modern structural studies further indicate that thin-filament regulation cannot be adequately described as a simple binary on–off mechanism. Instead, the troponin–tropomyosin complex exists in multiple dynamic conformational states that transition into each other during activation of muscle contraction, providing a more nuanced view of the structural mechanisms underlying Ca^2+^-dependent regulation [76].

### Tissue-specific expression and ontogeny

The troponin complex exhibits highly coordinated tissue-specific expression that reflects the structural and functional specialization of striated muscle tissues. It consists of three regulatory subunits-troponin C, troponin I, and troponin T-each encoded by distinct gene isoforms with characteristic expression profiles [37]. The cardiac troponin complex comprises the subunits TNNT2, TNNI3, and TNNC1. In fast skeletal muscle fibers, the troponin complex consists of TNNT3, TNNI2, and TNNC2, whereas slow skeletal muscle fibers express TNNT1, TNNI1, and TNNC1 [52]. In the adult human body, troponin subunit expression is highly selective [51].

During embryonic development, the expression of sarcomeric proteins and the myogenic differentiation of muscle cells are regulated by specific transcription factors [79–82]. These factors include MyoD, Six1, Six4, Sox6, Prdm1, Prox1, GTF3, PGC-1α, and Mef2. They play crucial roles in skeletal muscle fiber differentiation and influence the expression of troponin isoforms characteristic of adult fast and slow muscle fibers [51, 83–89]. Analogous transcription factors, including GATA-4, TBX5, and Myocardin, are involved in the development of cardiomyocytes and have been shown to regulate the expression of cardiac troponin isoforms [90, 91]. This regulatory network ensures the selection of troponin subunits appropriate for each muscle cell type while also enabling isoform switching through changes in transcription factor levels [51].

Troponin T isoforms provide a clear example of such developmental switching. Cardiac TNNT2 is expressed in embryonic skeletal muscle during development [39, 92, 93], whereas the slow skeletal muscle isoform TNNT1 is transiently expressed in embryonic cardiac tissue [94, 95]. In contrast, TNNT3 is exclusively expressed in fast skeletal muscle [96]. Troponin T also undergoes alternative splicing during development [39]. Similarly, the slow skeletal muscle isoform TNNI1 is expressed in developing cardiomyocytes and is subsequently replaced by the cardiac isoform TNNI3 as the heart matures [42, 97, 98]. In contrast, both cardiac TNNI3 and fast skeletal TNNI2 exhibit highly restricted expression in their respective cell types [99, 100]. Although TNNC1 is predominantly associated with cardiac and slow skeletal muscle in adulthood, it is also transiently expressed during the development of fast skeletal muscle fibers [101–104]. TNNC2, by contrast, is expressed exclusively in fast skeletal muscle cells [51, 105]. Unlike troponin T, neither troponin I nor troponin C is known to undergo alternative splicing during development [51, 55].

Developmental switching of troponin isoforms has important structural and functional consequences. In cardiomyocytes, changes in troponin isoform expression are associated with the maturation of sarcomere structure [106]. During cardiac ontogenesis, the myocardium also undergoes substantial changes in calcium sensitivity, which can be largely attributed to the replacement of TNNI1 by TNNI3 [107, 108]. In addition to exhibiting higher calcium sensitivity [107], the embryonic cardiac troponin complex shows reduced responsiveness to calcium sensitizers [109, 110] and diminished sensitivity to adrenergic stimulation [110, 111]. In this context, the N-terminal cardiac-specific extension of TNNI3, which is absent from other TNNI isoforms, appears to play a critical role. This domain undergoes protein kinase A (PKA)-dependent phosphorylation in response to β-adrenergic signaling. This phosphorylation is thought to mediate the effects of β-adrenergic signaling on sarcomere contractility [110, 112].

Isoform switching is not limited to normal muscle development. Re-expression of embryonic troponin variants has been reported in heart failure, where they may partially replace adult isoforms. However, the functional consequences of this phenomenon remain incompletely understood [106, 113, 114].

### Post-translational regulation of troponin

Troponin is a regulatory protein complex composed of troponin C, I, and T, which controls calcium-dependent contraction and relaxation in cardiac muscle by regulating actin–myosin interactions [101]. After translation, troponin undergoes several post-translational modifications (PTMs), including phosphorylation, oxidation, and proteolytic cleavage [39, 40, 42, 43]. These modifications alter troponin structure and function, thereby influencing calcium sensitivity, myocardial contractility, and overall cardiac performance in both physiological and pathological states.

Phosphorylation of cardiac troponin I (cTnI) is the best-characterized PTM [45, 115–117]. Under normal conditions, PKA, activated by β-adrenergic signaling, phosphorylates cTnI and reduces calcium sensitivity, which promotes faster myocardial relaxation during stress [42, 115]. In heart failure, this signaling is disrupted, and abnormal protein kinase C (PKC)-mediated phosphorylation contributes to reduced contractile force and maladaptive cardiac remodeling [45–49]. These changes in phosphorylation balance are closely linked to systolic and diastolic dysfunction [118, 119]. Oxidative stress during ischemia or chronic cardiac disease leads to oxidative modifications of troponin, which impair actin–myosin interactions and reduce contractile efficiency [43, 120, 121]. Another important PTM is proteolysis [39, 42], where calcium-dependent calpains and caspases cleave cardiac troponin I and T during necrosis or apoptosis [122–124]. This damages the troponin complex and produces fragments that are released into the bloodstream [125].

Therapeutically, several strategies aim to correct dysregulated troponin signaling pathways. β-blockers such as metoprolol, bisoprolol, and carvedilol reduce chronic β-adrenergic overstimulation and indirectly help normalize phosphorylation signaling [126, 127]. Levosimendan is currently used primarily in the treatment of acute and advanced heart failure, where it improves myocardial contractility by sensitizing troponin C to calcium without significantly increasing intracellular calcium levels [128–130]. Experimental approaches also include PKC inhibitors and cardiac myosin activators like omecamtiv mecarbil, which aim to improve systolic function and reduce pathological remodeling [131, 132].

Overall, troponin PTMs play a central role in regulating cardiac function, and their dysregulation contributes directly to heart failure and ischemic injury. Understanding these molecular mechanisms is essential for developing targeted therapies in cardiovascular disease. Troponin in Cellular and Extracellular Compartments.

## Molecular mechanisms underlying the non-canonical functions of troponin

### Nuclear localization and functions

Previous investigations have emphasized the extra myofibrillar roles of troponin, particularly its functions beyond the traditional troponin–tropomyosin complex. Many of troponin’s observed effects can be attributed primarily to its localization and activity within the nucleus, which have been documented repeatedly in both muscle cells and various non-muscle cell types [133–135].

In cardiomyocytes, the presence of intranuclear troponin has been validated by immunofluorescence studies [134–136]. All three cardiac troponin subunits: C, T, and I, were detected in neonatal rat ventriculocytes stained five days after differentiation [134]. Cardiac troponin I was also identified in adult murine cardiomyocytes [137]. In human cardiomyocytes, cardiac troponin subunits T and I have likewise been reported to localize to the nucleus [135, 136]. However, these studies did not characterize the subnuclear localization of cardiac troponin subunits. Another cell type known to exhibit cytoplasmic troponin complex expression is skeletal muscle cells. Using TNNT3-DsRed (fast skeletal muscle troponin T-red fluorescent protein) fusion protein overexpression in the C2C12 mouse skeletal muscle cell line, Zhang et al. observed TNNT3 association with dense nucleolar structures. In addition to a fluorescent construct containing full-length TNNT3, several TNNT3 fragments were fused with DsRed protein. In this case, the nuclear staining pattern depended on the specific fusion construct used. Beyond fusion protein experiments, Zhang et al. also reported the presence of TNNT3 and its fragments in the nuclear fraction of non-transfected cell lysates following immunoblotting [133]. Variable nuclear staining patterns were also observed in cancer cell lines. In human lung adenocarcinoma cell line SPCA-1, human gastric adenocarcinoma cell line BGC 823, and human hepatocellular carcinoma cell lines Huh-7, MHCC-97L, and MHCC-97H, immunofluorescence patterns differed depending on the specific epitope targeted by anti-TNNT3 antibodies [138]. TNNC1 with added tag sequence was also found in nuclei of non-small cell lung adenocarcinoma (NSCLC) A549 cells using Anti-V5 epitope antibody [139, 140].

Taken together, these findings suggest that at least some troponin subunits exhibit specific subnuclear localization. Future studies examining the subnuclear localization of additional troponin subunits could thus provide important insights. Moreover, the distinct staining patterns observed with epitope-specific antibodies (which can also be interpreted as the presence of distinct troponin conformational states) and troponin T fragment–DsRed fusion proteins hint at the possibility that truncated troponin subunits are present within the nucleus. Such intranuclear troponin fragmentation may also have functional relevance, as the TNNT3 carboxy-terminal fragment colocalized with RNA polymerase I, whereas full-length TNNT3 recruited RNA polymerase II [133]. This area, therefore, represents a knowledge gap warranting further investigation.

Earlier studies revealed that several cytoskeletal proteins are present in the nucleus [141–144] and interact with nuclear lamins [145]. For example, actin-myosin complexes have been implicated in the positioning of the transcription apparatus and the regulation of transcriptional activity [146]. The intricate structure responsible for this activity is known as the nucleoskeleton. Extensive research [142, 146–150] has shown that the nucleoskeleton forms numerous interactions with chromatin and can modulate chromatin architecture, nuclear morphology, and gene expression both via the mechanical positioning of transcriptional complexes and by forming a protein scaffold.

In addition to their structural role within the nucleoskeleton, nuclear actin and lamins have also been shown to participate in nuclear protein complexes in their monomeric forms [151–153]. Non-polymerized A-type lamins are present in the nucleoplasm, where they associate with lamina-associated polypeptide 2α (LAP2α) [151, 152, 154]. This complex has been implicated in the activation of retinoblastoma tumor suppressor protein (pRb) mediated transcriptional repression, although the precise underlying mechanism remains incompletely understood [155]. Nuclear G-actin (monomeric globular actin) has been proposed to form part of the INO80 and SWR1 chromatin-remodeling complexes, as well as the NuA4 histone acetyltransferase complex [153]. These findings show that at least some nucleoskeletal proteins play a role in nuclear processes without the involvement of their mechanical partners. Given troponin’s well-established cytoplasmic role [37] and the confirmed presence of other cytoskeletal components in the nucleus [141–144], one might hypothesize that troponin exerts its nuclear functions via canonical interactions with actin–myosin elements. However, current evidence remains insufficient to confirm that. To date, no studies have conclusively demonstrated the incorporation of any troponin isoform into the nucleoskeleton. Several independent nuclear functions of troponin have been documented [28, 29, 156–158]; whether these require nucleoskeletal interactions remains unknown. These functions will be discussed in greater detail in the following sections.

There are also questions about the stoichiometry of troponin subunits in the nucleus. In muscle cells, specifically neonatal rat ventriculocytes, all three compatible subunits of the cardiac troponin complex have been detected [134]. However, due to the lack of detailed subnuclear localization data, it remains unclear whether these subunits colocalize within the same subnuclear compartments. Asumda et al. proposed the existence of troponin-containing complexes resembling the Ca^2+^-regulated thin filaments based on the nuclear presence of their constituent proteins but were unable to experimentally confirm their formation. As noted above, this topic therefore represents an important knowledge gap worth exploring.

It is also not clear if isoforms capable of assembling into a complete tissue-specific troponin complex coexist within the nuclei of individual non-muscle cells. Co-expression of cardiac and non-cardiac subunits of the troponin complex has been reported in embryonic muscle tissues [39, 94, 106] as well as under pathological conditions [159–163]. Furthermore, several studies have demonstrated the formation of troponin complexes composed of subunits with different tissue specificities [112, 164–166]. It is therefore plausible that these heterogeneous troponin isoforms interact with one another within the nucleus, potentially opening new avenues for their functional roles.

Bioinformatics analyses suggest that at least some cell types lack nuclear co-expression of mutually compatible triplets of troponin complex subunits. One such cell type is lung cancer cells, which are a relevant model given their non-muscular origin and the availability of non-omic studies confirming their expression of troponin [138, 140, 167, 168]. Data from the Expression Atlas database [169] reveal a lack of co-expression of cell type-specific troponin subunits and a high degree of variability in isoform expression levels. Similar patterns of inconsistent expression exist in many other cell lines [169]. As stated above, troponin monomers can exert nuclear functions independently of other components of the troponin complex, as demonstrated by Zhang et al. [157]. However, it remains unknown whether intranuclear troponin subunits colocalize within the same subnuclear compartments or whether they can function in dimeric or trimeric forms.

#### Nuclear localization signals and leucine zipper domains

As previously discussed, troponin subunits are present in the nuclear compartment of both muscle and non-muscle cells [133, 134, 138]. Proteins capable of crossing the nuclear membrane typically do so by binding to karyopherin proteins. These karyopherins interact with small Ran GTPases, whose nucleotide state (GTP- or GDP-bound) regulates the ability of karyopherins to bind and transport their cargo [170–173]. Not all proteins can interact with karyopherins. Only proteins containing specific nuclear localization signals (NLS) or nuclear export signals (NES) within their sequences are able to do so [174–176]. Given the experimentally confirmed intranuclear presence of troponin [133, 134, 138], it is plausible that it contains an NLS within its sequence.

Bioinformatics methods were used to identify putative NLS in troponin. Bergmann et al. employed the PSORT II [177] subcellular localization prediction tool and reported that the TNNI3 and TNNT2 subunits contain four putative NLS sequences, suggesting that troponin may be translocated into the nucleus via specific mechanisms. However, the exact predicted sequences were not specified [136].

In addition to the NLS, the C-terminal region of another troponin isoform, TNNT3, presumably contains a leucine zipper domain (LZD) [178], a well-known motif commonly involved in the binding of transcription factors to DNA [179, 180]. PDBsum analysis [181] predicted that mouse troponin isoforms TNNT1 and TNNT2 contain classical leucine zipper domains, whereas TNNT3 contains a relaxed leucine repeat [182–186], in which the fourth leucine is replaced by another hydrophobic amino acid, phenylalanine. It was also shown that this motif is conserved across mammalian species. The human TNNT3 predicted non-canonical LZD sequence differs from the mouse ortholog by two substitutions: tyrosine to histidine at position 13 and threonine to isoleucine at position 17 [178]. To further investigate the functional relevance of this motif, Zhang et al. removed the LZD from TNNT3 by truncation and by substituting the second and third leucine residues with alanine. This resulted in reduced apoptosis in skeletal muscle cells expressing the altered TNNT3 protein. Importantly, nuclear localization of TNNT3 was not affected, suggesting that the LZD is not required for nuclear import but may be involved in apoptotic signaling pathways [178].

**Fig. 2:**
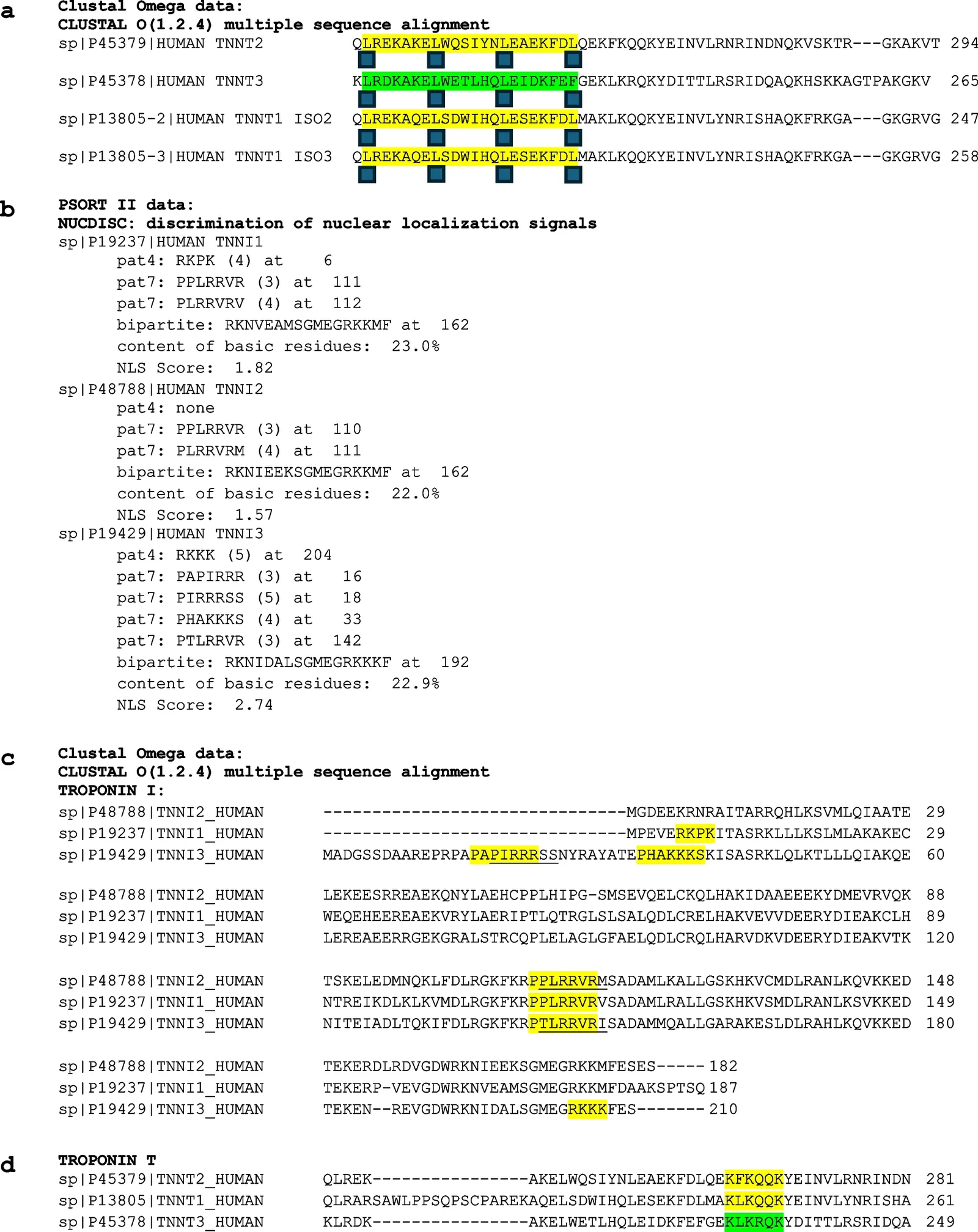
We employed bioinformatic tools to further elucidate nuclear localization sequences (NLSs) and leucine zipper domains (LZD) in structure of troponin complex subunits. We used PSORT II web based subcellular localization prediction tool, to look for NLSs and Clustal Omega tool for multiple sequence alignment of troponin isoforms. All sequences were downloaded from Uniprot database. **a** TNNT3 non-canonical LZD sequence is highlighted in green. Canonical LZD motifs of TNNT2 and isoforms 2 and 3 of TNNT1 are highlighted in yellow. Leucines (L) and phenylalanine (F) are marked by squares. Presence of non-canonical LZD motif in mouse TNNT3 was proposed and supported by changes in apoptosis induced by mutation in the proposed LZD sequence. The human TNNT3 also has a predicted non-canonical LZD sequence that differs from the mouse ortholog only by two substitutions: tyrosine to histidine at position 13 and threonine to isoleucine at position 17. Other troponin T isoforms sequences include predicted cannonical LZD motif. Isoform 1 of TNNT1 is not included in the picture, because it does not include canonical LZD motif. It is plausible that it contains non-cannonical LZD. **b** PSORT II prediction of putative NLSs in TNNI isoforms. Human TNNI3 NLSs were tested and all execept the bipartite one, shown diminished nuclear localization after their dysabeling mutation. Specific sequences of predicted NLSs are shown with their localization in protein sequence. **c** Putative NLSs are highlighted in green. Overlapping NLS sequences are underlined. Monopartide NLSs of TNNI3 were experimentally tested. NLSs of other troponin I isoforms are putative. Bipartite NLSs are not shown. **d** NLS of human TNNT3 that has the same sequences as experimentally verified mouse TNNT3 NLS is highlighted in green. Corresponding sequences in TNNT1 and 2 are highlighted in yellow. TNNT3 NLS contains the canonical classical NLS consensus motif K-(K/R)-X-(K/R). Corresponding sequences do not contain this motif, but are still short, basic amino acid-rich sequences, which is characteristic of NLS motifs

We employed bioinformatics tools to search for possible LZD motifs in other human troponin T subunits. TNNT2 and two of the three TNNT1 isoforms contain canonical LZDs [187] (Fig. 2). It can also be reasonably assumed that the third TNNT1 isoform contains non-canonical LZD. While the LZD motif is common and its presence alone does not confirm DNA binding, the combination of experimentally verified DNA interactions [157, 188] and high evolutionary conservation suggests it may be functionally relevant even in untested isoforms of troponin T.

The NLS sequence identified in this study [178] differs from other such motifs predicted using software tools. The deletion of the NLS sequence failed to alter mouse TNNT3 nuclear localization. This novel, previously unpredicted NLS features a KLKRQK motif. Using in silico methods, the authors also identified the same monopartide NLS motif in the rat, baboon, and human orthologs of mouse TNNT3 [178]. We compared this sequence with corresponding regions in human TNNT1 and TNNT2. Although they do not contain the canonical classical NLS consensus motif K-(K/R)-X-(K/R), they are still short, basic amino acid-rich sequences, which is characteristic of NLS motifs [175] (Fig. 2). In addition to the NLS sequence, the C-terminus of TNNT3 contains a nuclear export signal (NES). This was demonstrated by the increased cytoplasmic localization of a TNNT3 variant with a truncated C-terminus that lacked the NLS but retained a predicted NES sequence located within the GATAKGKVGGRWK region [178].

In a 2020 article, Kharnitov et al. studied NLS sequences of another troponin isoform with proven nuclear localization, TNNI3 [189, 190]. They tested the functionality of PSORT II predicted sequences by substituting all positively charged amino acids in each predicted NLS sequence with alanines. To visualize the changes in nuclear localization, a fusion protein composed of human TNNI3 and EGFP (enhanced green fluorescent protein) was used. Mutations in all predicted sequences, except for the bipartite sequence, influenced nuclear localization, demonstrating their functionality [190]. These sequences are visualized in Fig. 2. This study further demonstrated that TNNI3 enters the nucleus via both passive diffusion through nuclear pores and the karyopherin-α/β import cascade [171, 191], with the latter being responsible for its significant nuclear accumulation. It should be noted that this accumulation was observed in undifferentiated human myoblasts, and the nuclear fluorescent signal emitted by EFGP-TNNI3 was lost as differentiation proceeded and myofibrils formed. The most plausible explanation for this is that TNNI3 becomes incorporated into newly formed myofibrils, depleting its unbound cytoplasmic fraction. This dynamic redistribution suggests that troponin may be involved in the replication of genes essential for proper myoblast differentiation but that its function is effectively silenced upon incorporation into developing myofibrils [190].

Another level of regulation of troponin's nuclear function was identified in research on embryonic cell division and apicobasal differentiation in the Drosophila melanogaster S2 cell line. In this context, troponin I localized to the nucleus only after undergoing SUMOylation. This post-translational modification may be necessary for its nuclear import, given the absence of any apparent NLS sequence within the Drosophila TnI structure [189]. SUMOylation has previously been shown to facilitate nuclear transport in other proteins [192, 193], including foreign proteins such as adenoviral DNA-binding protein E2A [194]. It is therefore plausible that SUMOylation serves a similar function in this case. As discussed above, human troponin I isoforms contain both predicted and experimentally validated NLS sequences [190]; therefore, they do not require SUMOylation for nuclear entry. SUMOylation of human TNNI3 has been reported, although its functional significance remains unknown [195]. Further research is needed to determine whether SUMOylation of troponin I plays a role in models other than Drosophila.

To clarify the presence and role of NLS motifs in troponins, we used the web-based tools PSORT II [177] for nuclear localization signal prediction and Clustal Omega [187] for multiple sequence alignment. The resulting predicted occurrence of NLS motifs across troponin I isoforms is shown in Fig. 2, alongside NLS prediction results for individual troponin isoforms from PSORT II (Fig. 2). Although several NLS sequences were predicted for troponin T, we excluded them based on the findings of an earlier study on mouse TNNT3 [178] and only searched for motifs corresponding to the newly described NLS in humans. The occurrence of these motifs in troponin T isoforms is illustrated in Fig. 2. To date, no NLSs have been reported in troponin C isoforms, and PSORT II also does not predict any putative NLS sequences. Nevertheless, TNNC1 has been detected in the nuclei of neonatal rat ventricular cardiomyocytes [134] and NSCLC cells [140], suggesting that its nuclear localization is mediated by an as-yet unidentified mechanism and requires further study.

Although these findings are not definitive, they provide meaningful insights and establish a basis for further investigation. In the following sections, we will revisit this evidence to explore the potential functions of troponin in greater detail.

#### Regulation of transcription

Beyond its well-established structural role, troponin has emerged to be potentially involved in the regulation of gene expression, suggesting a broader and more complex function than previously recognized. Substantial evidence supports this expanded role.

For instance, research on possible troponin DNA binding sequences revealed the binding of TNNT3 to the TGCCT motif using ChIP-Seq analysis [188]. Interestingly, this motif appears in the promoter regions of several genes regulated by the p53 protein and is one of its known binding sites [196, 197]. This prompted an effort to determine whether fluctuations in TNNT3 expression affected the activity of p53-regulated genes. Elevated intracellular levels of TNNT3 were found to strongly correlate with the expression of p53-controlled ribonucleotide reductase M2 B (*RRM2B*), a p53-regulated gene containing the TGCCT motif. The gene *RRM2B* also represented one of the most enriched sequences for TNNT3 binding in ChIP-Seq analysis [188]. Ribonucleotide reductases are enzymes responsible for the reduction of ribonucleoside diphosphates to deoxyribonucleotides, a crucial step in DNA synthesis [198]. RRM2B specifically plays a role in DNA repair, mitochondrial DNA (mtDNA) synthesis, cell cycle regulation, and resistance to oxidative stress [199]. Whether TNNT3 binding to the promoter regions of p53-regulated genes interferes with p53 function or instead supports it by activating these genes remains unknown and may be context-dependent [188].

Moreover, an earlier study had shown that TNNT3 binds to a similar motif (ATCTGCC—AG) in the promoter of the *Cacna1s* gene, encoding the α1S subunit of the CaV 1.1 calcium channel [157]. These results support the conclusion that TNNT3 likely binds to gene sequences regulated by p53—a finding that may partly explain troponin’s involvement in cancer development, given the central role that p53 plays in oncogenesis [200].

Unlike TNNT3, TNNI3 lacks a clearly defined DNA-binding sequence. However, there is evidence that it can nevertheless modulate gene expression: the expression of the *ATP2A2* gene (which encodes the sarcoplasmic reticulum calcium pump) increased upon TNNI3 overexpression and decreased significantly upon TNNI3 knockout. A subsequent ChIP-Seq analysis revealed an association between TNNI3 and the *ATP2A2* gene promoter, which was further confirmed by ChIP-qPCR. The exact binding sequence for TNNI3 was computationally analyzed as CCAT [158]. This sequence was previously shown to interact with the Yin-Yang 1 (YY1) protein [201–203]. To investigate a potential interaction between TNNI3 and YY1, the study authors performed immunoprecipitation experiments, which confirmed an interaction between TNNI3 and YY1 [158]. YY1 is a transcription factor implicated in the regulation of cell proliferation, apoptosis, embryonic development, and DNA damage repair [204–206]. As its name suggests, the Yin-Yang 1 protein exhibits dual and often antagonistic regulatory functions, acting either as a transcriptional activator or repressor depending on the context [207, 208]. This functional duality can be explained by two proposed mechanisms: post-translational modifications of YY1 and its association with cofactors [209–211]. These cofactors may act as co-activators (E1A, YY1AP) or co-repressors (mRPD3, SAP30) [211–215], thereby modulating YY1’s ability to bind promoter sequences of target genes [212]. Since overexpression of TNNI3 leads to increased expression of the *ATP2A2* gene, it can be hypothesized that, in this context, TNNI3 acts as a co-activator of YY1.

A follow-up study found an association between lower autophagy and lower TNNI3 intranuclear levels in heart muscle tissue. In this study, one of the genes, whose expression was altered the most by differences in TNNI3 expression, was the *FOS* gene. Interaction between the *FOS* gene promoter and TNNI3 was validated using ChIP‐q‐PCR [137]. Gene product c-Fos interacts with c-Jun protein, and together they form the heterodimeric activator protein 1 (AP-1) transcription factor. As a part of this heterodimer, c-Fos influences proliferation, differentiation, apoptosis, and inflammation [216]. YY1 was also previously shown to bind the promoter region of the *FOS* gene [217–219]. Authors of the study therefore suggested that TNNI3 acted as a co-activator of YY1, with its lower nuclear levels causing downregulation of FOS, which they connected with lower transcription of central autophagy regulator ATG5 [220] and impaired autophagy [137].

It should be noted that modulation of gene expression does not always occur through direct binding to regulatory DNA sequences. For example, the fast muscle troponin TNNI2 affects gene expression indirectly via its interaction with the estrogen receptor-related receptor α (ERRα) [221]. ERRα is an orphan receptor, i.e., a receptor with no known endogenous ligand, and was one of the first such receptors to be discovered in humans [222]. Despite having no known ligand, ERRα exerts significant influence over gene transcription, as do all members of the entire ERR receptor family [223]. Instead of endogenous ligands, its activity is modulated by coactivators such as TNNI2. TNNI2 exerts its regulatory effect by interacting with ERRα via its LXXLL motif [221]. This motif is also known as the NR box and is commonly found in the structure of nuclear hormone receptor coactivators [224]. The interaction between ERRα and TNNI2 was validated using a yeast two-hybrid (Y2H) assay, with LacZ serving as the reporter gene. The involvement of the LXXLL binding motif was verified by comparing Y2H results obtained using wild-type TNNI2 and an NR box–truncated TNNI2 construct [221].

**Fig. 3:**
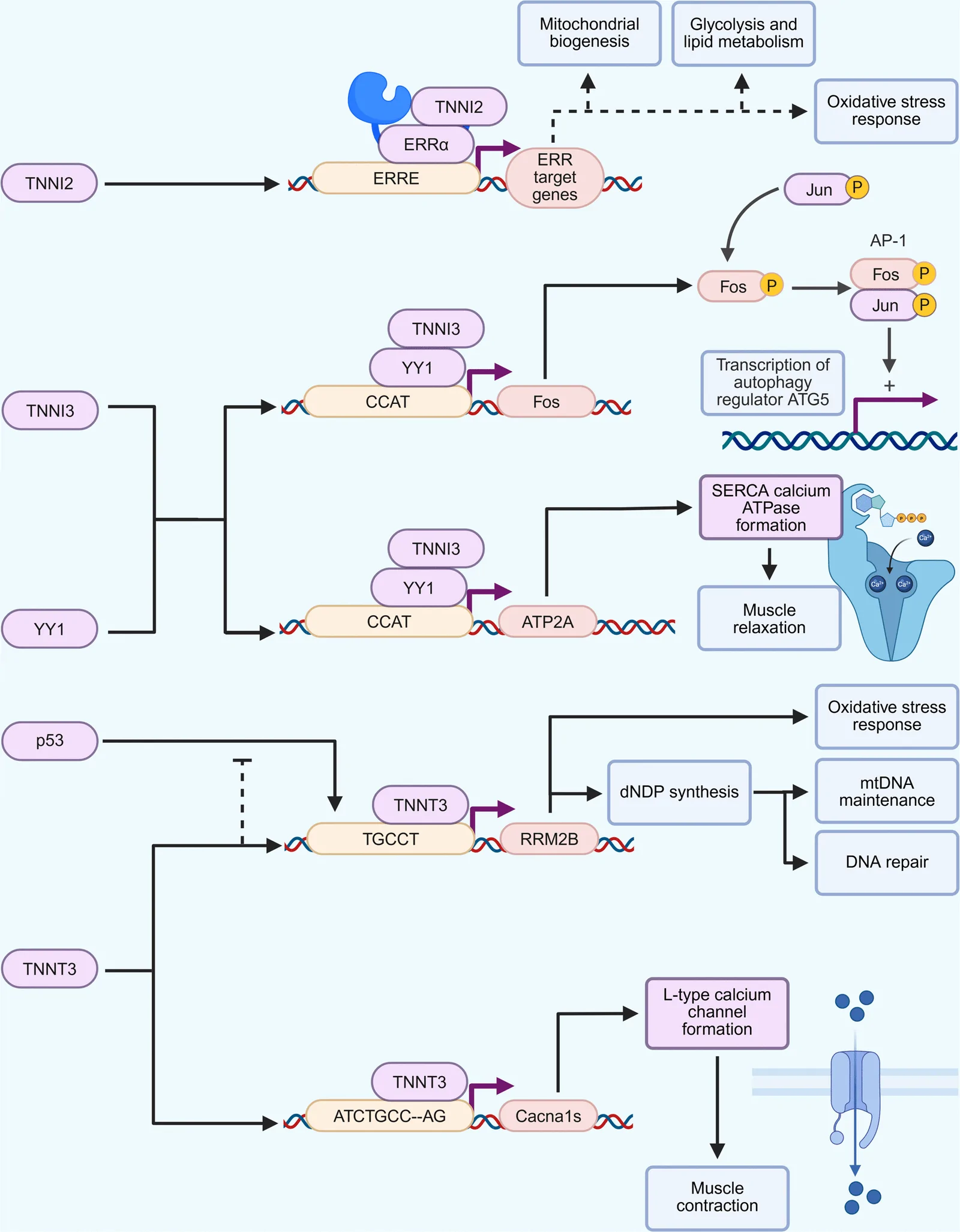
Several troponin isoforms are involved in the regulation of gene expression. TNNI2 binds to the nuclear receptor ERRα via its LXXLL motif and functions as a co-activator. The TNNI2-ERRα complex binds to ERREs and regulates the expression of ERRα downstream targets. Because ERRα target genes are co-activator-specific and those regulated specifically by TNNI2 have not been described, dashed lines are used to connect this complex to several known functions of ERRα. TNNI3 associates with the transcription factor YY1, which recruits cofactors (co-activators and co-repressors) that modulate its activity. Both TNNI3 and YY1 bind to the CCAT motif. The TNNI3-YY1 complex binds to the promoter of the *ATP2A2* gene and upregulates its expression. *ATP2A2* encodes SERCA2, which is essential for the reuptake of calcium into the sarcoplasmic reticulum following muscle contraction. The TNNI3-YY1 complex also binds to a CCAT motif in the promoter of the *FOS* gene, resulting in increased c-Fos expression. Although c-Fos has multiple functions, it also promotes the expression of the key autophagy regulator ATG5. Through this mechanism, TNNI3 enhances autophagy. TNNT3 increases the expression of *RRM2B* by interacting with a CCAT motif in the *RRM2B* promoter, which also serves as a p53-binding site [168]. Whether TNNT3 binding interferes with p53 signaling remains to be determined. RRM2B reduces ribonucleoside diphosphates to deoxyribonucleoside diphosphates, thereby contributing to the maintenance of mitochondrial DNA (mtDNA) integrity and DNA repair. RRM2B is also involved in the oxidative stress response. TNNT3 also upregulates *CACNA1S* expression by binding to an ATCTGCC--AG motif in its promoter. This supports the maintenance of excitation-contraction coupling and muscle strength. Abbreviations: ERRα: estrogen receptor–related receptor α, ERRE: estrogen receptor–related receptor α response element, YY1: Yin Yang 1, ATG5: autophagy-related protein 5, ATP2A: gene encoding sarcoplasmic/endoplasmic reticulum calcium ATPase, SERCA: sarcoplasmic/endoplasmic reticulum calcium ATPase, RRM2B: p53-controlled ribonucleotide reductase M2B, dNDP: deoxyribonucleoside diphosphates, mtDNA: mitochondrial DNA, Cacna1s: α1S subunit of the CaV1.1 calcium channel

Multiple sequence alignment using Clustal Omega [187] revealed that the LXXLL motif of TNNI2 corresponds to the LRALL sequence in TNNI1. However, the precise mechanism by which TNNI2 binding alters ERRα function remains unclear. The ERRα receptor significantly influences the development of estrogen receptor-negative breast cancer [225] and helps regulate the cellular hypoxia response [226]. Interaction with ERRα may thus be another pathway by which troponin influences oncogenic processes. The targets of troponin transcriptional regulation are summarized in Fig. 3.

#### Epigenetic regulation

The expression of troponin subunits is tightly regulated, including through epigenetic mechanisms [227], to maintain the correct ratio of TNNC, TNNT, and TNNI in the troponin complex. However, two studies by Zhao et al. suggest that troponin can also function as an epigenetic regulator. Both of these studies focused on the mutant troponin variant R193H, which is responsible for the development of restrictive cardiomyopathy (RCM) [68, 228, 229]. It has been shown that a mutation in the C-terminal region of TNNI3 alters the Ca^2+^-regulated interaction of the troponin complex with tropomyosin through changes in the interaction of the TNNI3 C-terminal domain with tropomyosin, TNNT2, and TNNC1. This leads to elevated diastolic cardiac muscle tension and reduced myocardial compliance [68, 229]. Apart from its function in the sarcomere, TNNI3 also appears to be involved in the pathogenesis of RCM through less well-characterized mechanisms.

In cells expressing the TNNI3 R193H variant, phosphodiesterase 4D (PDE4D), a key negative regulator of β-adrenergic signaling in cardiomyocytes [230], is downregulated compared to cells expressing wild-type TNNI3. This downregulation coincides with the appearance of several epigenetic modifications consistent with gene repression in the *PDE4D* promoter region, including reduced histone acetylation (H3K4ac, H3K9ac) and trimethylation (H3K4me3). It was also observed that binding of histone deacetylase 1 (HDAC1) and the methyltransferase SET and MYND domain-containing protein 1 (SMYD1) to the *PDE4D* promoter region was increased [28]. The decrease in acetylation can be readily explained by increased HDAC1 binding. In this context, SMYD1 likely associates with HDAC1 and enhances its function. The interaction between HDAC1 and SMYD1 was confirmed by co-immunoprecipitation [28], and SMYD1’s role as a co-repressor acting in concert with HDAC1 has also been previously reported [231, 232]. The cause of reduced trimethylation remains unknown and given the established opposing effects of SMYD1 on H3K4 methylation [233], this change cannot be explained by its increased binding. In a follow-up study, co-immunoprecipitation experiments demonstrated that TNNI3 interacts with HDAC1. In a primary neonatal mouse cardiomyocyte model carrying the TNNI3 R193H mutation, this interaction was stronger and was associated with reduced PDE4D expression [29]. Thus, TNNI3 appears to influence the epigenetic regulation of PDE4D expression. The mechanism by which decreased PDE4D expression contributes to RCM remains unclear, although PDE4D has previously been implicated in heart failure and dilated cardiomyopathy [230, 234].

R193H-associated RCM is not the only disease in which troponin has been found to interact with proteins involved in epigenetic regulation. Cardiac troponin T R173W is a mutant variant of troponin T associated with the development of dilated cardiomyopathy (DCM) [235]. It was shown that these troponin variants are located more in the nucleus, compared to wild-type TNNT2. The authors proposed that this change results from alteration of a nuclear localization signal (NLS) by the R173W mutation [236]. The region affected by this mutation contains the basic amino acid-rich sequence RKK; however, PSORT II [177] does not identify any NLS within this region. A similar sequence, KRR, is present at the corresponding position in TNNT3 and has previously been shown not to influence its nuclear localization [178]. Furthermore, substitution of arginine with tryptophan would be expected to reduce, rather than enhance, nuclear localization potential. Taken together, these observations suggest that the altered nuclear localization is unlikely to result from direct disruption of an NLS.

Co-immunoprecipitation experiments further demonstrated that TNNT2 interacts with histone demethylases KDM1A and KDM5A, as well as histone H3. This interaction was increased in cells expressing mutant TNNT2. The authors speculated that this enhanced interaction might lead to epigenetically mediated upregulation of PDE2A and PDE3A expression [236]. However, there is currently no direct evidence supporting this hypothesis. Apart from histone acetylation and methylation, other epigenetic mechanisms, such as non-coding RNA-mediated regulation and DNA methylation, also exist [237, 238]. Whether troponin is involved in these regulatory pathways remains unknown. However, its interactions with multiple epigenetic regulators suggest that further investigation may be warranted.

### Ca^2+^ signaling crosstalk

Calcium signaling plays a crucial role in oncogenesis, making proteins involved in this pathway promising drug targets [239]. The interaction between troponin and calcium is well known [37], but are there other non-traditional mechanisms by which troponin affects Ca^2+^ signaling?

Zhang et al. found that the LZD motif of TNNT3 enables an unusual effect on Ca^2+^ signaling: TNNT3 interacts with the β1 subunit of the voltage-gated calcium channel (VGCC) family of complexes and causes the subunit to become localized in the nucleus [156]. Searches of the Expression Atlas database [169] revealed that the β1 subunit is expressed in heart and brain tissue but is also upregulated in several types of tumors [240]. Moreover, in addition to its interaction with the VGCC complex, the β1 subunit modulates calcium-mediated gene expression [241, 242]. TNNT3 thus indirectly regulates Ca^2+^ signaling. TNNT3, as a transcriptional regulator, upregulates the expression of the *Cacna1s* gene. Gene encoding the α1S subunit of the dihydropyridine receptor provides another unexpected avenue by which troponin influences a VGCC component, and by extension, Ca^2+^ signaling. The interaction between TNNT3 and the *Cacna1s* promoter has been thoroughly characterized, revealing the key role of the LZD motif in TNNT3 [157].

Troponin thus interferes with calcium signaling in multiple ways [156, 157]. Given the critical role of Ca^2+^ signaling in carcinogenesis [239] and the reported upregulation of TNNT3 in certain cancers [32], it is reasonable to hypothesize that troponin influences pro-oncogenic Ca^2^⁺ signaling.

### Troponin in the extracellular environment

In addition to the above-mentioned unconventional functions of troponin, its potential involvement in intercellular communication via microvesicles should be considered. This form of signaling has recently attracted considerable attention in cancer research because it appears to play a significant role in establishing a suitable microenvironment for tumor growth [243].

Unfortunately, evidence for the presence of troponin within microvesicles remains limited and fragmentary, originating from transcriptomic and proteomic studies that were not specifically focused on troponin. According to data from the Vesiclepedia database [244], troponin has been detected in microvesicles originating from several cancer types, including melanoma and breast cancer. Table 1 presents an overview of these findings, including information on whether the evidence was found at the mRNA or protein level. Given the incompleteness of the available data, this topic is not explored further here.

**Table 1:** Isoforms of troponin present in cancer-related extracellular vesicles according to the Vesiclepedia database

Type of troponin	Gene	Species	Origin of vesicles	Type of vesicle	Evidence on the mRNA level	Evidence on the protein level
Troponin T1, slow skeletal type	TNNT1	*Homo sapiens*	colorectal cancer cells, glioblastoma cells	microvesicles	yes	
Troponin T3, fast skeletal type	TNNT3	*Homo sapiens*	brain cancer cells, breast cancer cells, colorectal cancer cells, kidney cancer cells, melanoma cells, ovarian cancer cells	extracellular vesicles		yes
Troponin I3, cardiac type	TNNI3	*Homo sapiens*	colorectal cancer cells	microvesicles	yes	
Troponin C1, slow skeletal and cardiac type	TNNC1	*Homo sapiens*	brain cancer cells, colorectal cancer cells, kidney cancer cells, leukemia cells, lung cancer cells, melanoma cells, ovarian cancer cells	extracellular vesicles (in case of colorectal cancer, cells are also present in microvesicles)	yes (on both protein and mRNA levels, only in colorectal cancer cells)	yes
Troponin C2, fast skeletal type	TNNC2	*Homo sapiens*	ovarian cancer cells	exosomes		yes
Troponin C2, fast skeletal type	TNNC2	*Mus musculus*	melanoma cells	extracellular vesicles		yes

### Inhibition of angiogenesis

Angiogenesis is a physiological process of new blood vessel formation that plays a significant role in various pathological processes, including tumor growth [245]. Due to its central role in disease development, targeting angiogenesis with specific inhibitors has emerged as a promising therapeutic approach.

The first established link between troponin I and angiogenesis dates to 1999, when Moses et al. explored the avascular properties of cartilage. Their work demonstrated the inhibitory effect of TNNI2 on angiogenesis: systemic administration of TNNI2 in vivo inhibited angiogenesis despite stimulation with basic fibroblast growth factor (bFGF or FGF2) [246]. Subsequent research using TNNI2-derived peptides identified the specific sequence responsible for its inhibitory effect, showing that vascular growth was suppressed only when the Glu94–Leu123 peptide was present, whereas the control peptide comprising residues 124–181 had no such effect [31]. Despite these findings, the mechanism by which TNNI2 inhibits angiogenesis remains unclear. As troponin only exhibits its effect in the presence of angiogenic stimuli (i.e., bFGF or VEGF) and not under baseline conditions, it can be assumed that it disrupts the signaling pathways associated with these growth factors [246]. This conclusion is supported by the increased expression of bFGF and VEGF-D after exposure of Morris hepatoma cancer cells to elevated TNNI2 levels [35]. However, further studies are needed to provide direct evidence for this proposed mechanism.

### Mitochondrial functions

Cancer cells exhibit a set of defining characteristics that differentiate them from normal cell populations, collectively referred to as the hallmarks of cancer. One such hallmark is the reprogramming of cellular energy metabolism [247]. It is therefore interesting that one of troponin’s unorthodox functions is performed in the mitochondria – the central organelles of cellular metabolism.

The TNNI3 R193H mutation, linked to restrictive cardiomyopathy, alters calcium sensitivity [248] and influences PDE4D expression through interaction with HDAC1 [29]. Additionally, this mutation influences mitochondrial activity and induces morphological changes observable by transmission electron microscopy, including vacuolization, breaks in cristae, and signs of mitophagy. These changes are accompanied by impaired mitochondrial function in the form of decreased ATP production in the respiratory chain.

Western blotting revealed that the R193H mutation reduced levels of several mitochondrial metabolism-related proteins, including ND5 (ubiquinone oxidoreductase chain 5), LRPPRC (a leucine-rich protein of the pentatricopeptide repeat family), and PGC-1α (PPARG co-activator 1 alpha) [249]. However, the mechanism underlying these changes remains unclear. Current interaction databases such as IntAct do not list any direct interactions between TNNI3 and these proteins [250]. While this lack of evidence does not preclude such interactions, it does suggest the potential involvement of alternative mechanisms such as changes in epigenetic regulation. The R193H troponin variant was experimentally demonstrated to affect HDAC1 activity; given the diversity of histone deacetylase targets, an epigenetic mechanism seems at least plausible, although it requires further investigation.

Another study [30] offered some preliminary insights into the mechanisms by which troponin influences mitochondrial function, revealing a surprising direct interaction between TNNI3 and mitochondrial F1F0 ATP synthase. It should be clarified, however, that this research used a different mouse model, namely a model of myocardial infarction induced by ligation of the LAD (left anterior descending artery) and subsequent reperfusion injury. During cardiomyocyte ischemia, the breakdown of the troponin–tropomyosin complex leads to an increase in free cytoplasmic troponin subunits, including TNNI3. Free TNNI3 then migrates to the mitochondrial compartment, significantly increasing its levels there relative to those seen under non-ischemic conditions. Within mitochondria, TNNI3 binds to F₁F₀ ATP synthase, specifically to its d subunit. This interaction requires the N-terminal region of TNNI3, which binds to the peripheral stalk region of the ATP synthase, as is consistent with modeling data from the Molecular Operating Environment (MOE) and the results of a proximity ligation assay (PLA). Molecular Operating Environment is a software platform used for molecular modeling, molecular docking, and drug discovery [251], whereas proximity ligation assay is an immunoassay used to detect protein–protein interactions through DNA signal amplification triggered by the proximity of two target molecules [252–255]. TNNI3 binding reduced ATP synthase activity while increasing hydrolase activity and ATP breakdown, disrupting mitochondrial energy metabolism and promoting cellular damage.

**Fig. 4:**
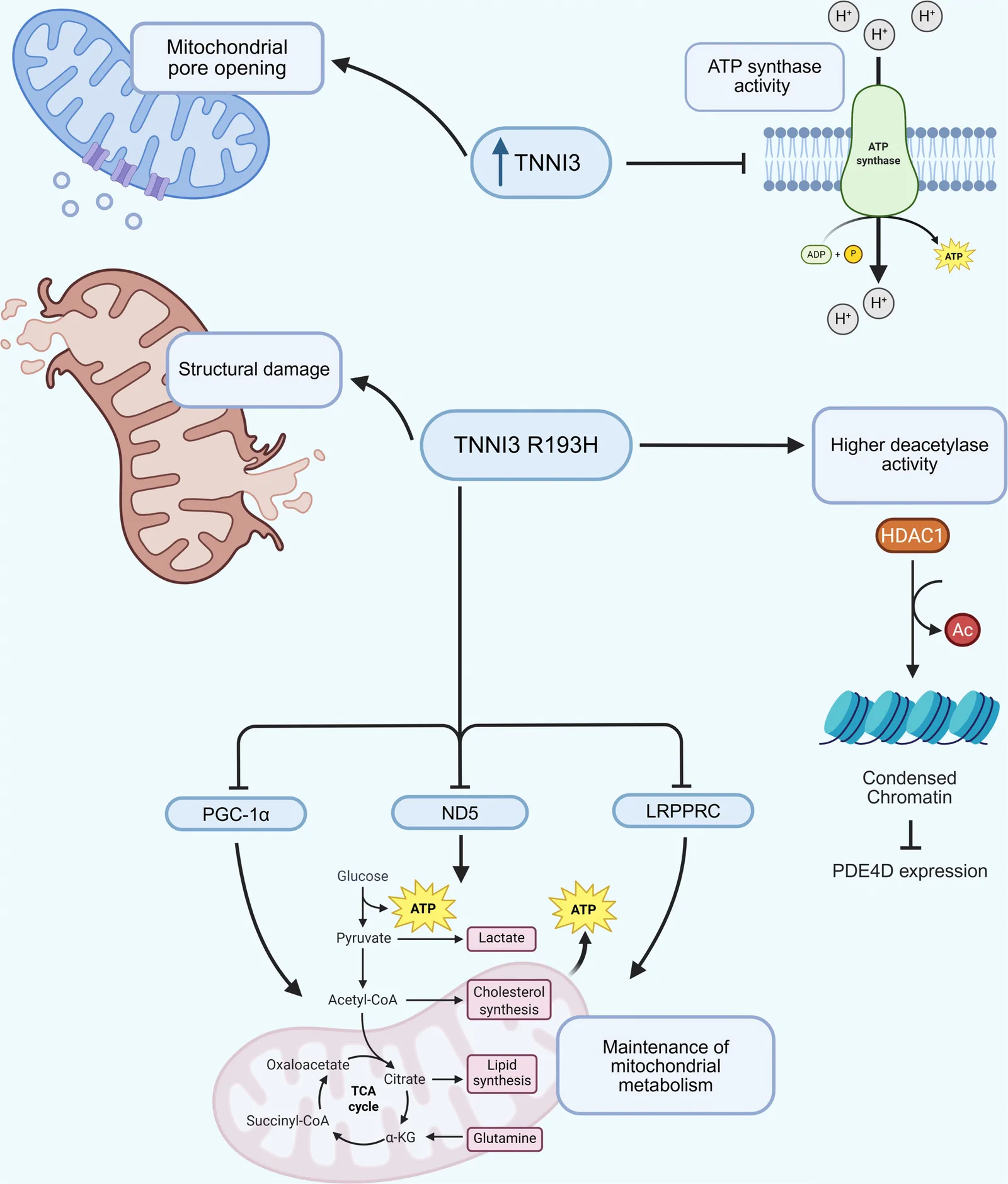
Summary of the effects of troponin isoforms on mitochondria. TNNI3 interacts with the d subunit of mitochondrial F₁F₀-ATP synthase. This interaction requires the N-terminal region of TNNI3, which binds to the peripheral stalk of the F₁F₀-ATP synthase complex. Increased TNNI3 binding reduces ATP synthase activity while enhancing its ATP hydrolase activity and ATP degradation. These changes disrupt mitochondrial energy metabolism and promote cellular damage. Elevated TNNI3 levels also increase the permeability of the mPTP, thereby promoting apoptosis. TNNI3 R193H mutant variant interacts with HDAC1 and enhances its binding to the promoter region of the *PDE4D* gene. This leads to reduced histone acetylation and repression of PDE4D expression. Expression of the TNNI3 R193H is also associated with decreased levels of proteins involved in mitochondrial metabolism, including ND5, LRPPRC, and PGC-1α. The mechanism by which TNNI3 R193H causes these changes remains unknown. The same mutation is also associated with mitochondrial structural damage, including vacuolization, disrupted cristae, and signs of mitophagy. Abbreviations: mPTP: mitochondrial permeability transition pore, HDAC1: histone deacetylase 1, PDE4D: phosphodiesterase 4D, ND5: ubiquinone oxidoreductase chain 5, LRPPRC: a leucine-rich protein of the pentatricopeptide repeat family. PGC-1α: PPARG co-activator 1 alpha

The final observed change was a faster decline in the mitochondrial TMRM fluorescence signal. Comparing these results with fluorescence measurements in cells treated with cyclosporine A suggested that this accelerated decrease was caused by changes in the permeability of the mitochondrial permeability transition pore (mPTP). The translocation of TNNI3 into mitochondria thus appears to enhance mPTP permeability, thereby promoting the activation of proapoptotic pathways [30]. The effect of troponin on mitochondria is summarized in Fig. 4.

Further study of other troponin subunits and their variants is needed to better understand troponin’s interactions with mitochondrial proteins. Different forms of TNNI3 may interact uniquely with F₁F₀ ATP synthase, which could be important in the development of cardiomyopathies [30]. However, only a few studies [249] have explored this topic. Additionally, variations in TNNI3 phosphorylation, especially in the N-terminal domain, might affect its binding to ATP synthase [256].

Another important question relates to the size of the free cytoplasmic pool of troponin subunits. While total cytoplasmic levels of TNNI3 (6–8%) and TNNT2 (2–4%) have been reported [257, 258], little is known about the dynamics of this pool other than that these proteins are rapidly released into the cytoplasm during myocardial infarction [259]. However, it is precisely this dynamic that is key to gaining a clearer understanding of the role of free troponin and the potential importance of TNNI3’s interactions with ATP synthase under various conditions, including physiological ones.

Dysfunction of mitochondria and of oxidative phosphorylation is characteristic of cancer cells [260–265]. This dysfunction is caused by multiple factors, with one of them being inhibition of ATP synthase by endogenous inhibition factor 1 (IF1) [266–269]. IF1 inhibits ATP synthase by binding the βE-subunit of the F₁ domain and blocks the rotation of the γ-subunit [270]. Interestingly, phosphorylation of IF1 in S39 prevents its interaction with ATP synthase [266]. As discussed previously, TNNI3 also affects ATP synthase activity, but unlike IF1, it interacts with the d-subunit of the F₀ domain of ATP synthase. TNNI3 also does not include a motif apparently similar to IF1 N-terminal inhibitory region [270]. Other ATP synthase inhibitors exist but are of exogenous origin. They interact with multiple different ATP synthase subunits and are structurally diverse [271, 272]. Whether TNNI3 affects ATP synthase in cancer is currently unknown, and further research in this field is required.

## Troponin in tumorigenesis

The previous chapters documented several unconventional roles of troponin beyond its conventional function in the troponin-tropomyosin complex, including its involvement in inhibiting angiogenesis and mediating nuclear signaling. It is not surprising that a protein involved in such a wide range of cellular processes is also implicated in tumorigenesis.

Aberrant troponin expression has been reported in multiple cancer types. A nearly comprehensive overview is provided by Johnston et al. [32], who compiled and contextualized data from online databases on the presence of troponin in cancer cells. Moreover, many studies, especially in recent years, have linked changes in troponin expression to altered cancer cell behavior that may correlate with either favorable [140] or unfavorable [273, 274] prognoses. However, these findings remain somewhat disorganized and often lack detailed descriptions of troponin-induced intracellular changes. The following sections summarize current knowledge about troponin’s impact on cancer cell behavior and explore mechanisms that may underpin these effects.

### Isoform-dependent functions of troponin I

The TNNI subunit of troponin has three isoforms, TNNI1–3, whose effects in cancer cells are often antagonistic. Unlike humans, Drosophila expresses only one troponin I isoform encoded by the wupA gene, whose overexpression in the Drosophila wing disc accelerates cell proliferation. Analysis of the causative factors of this enhanced proliferation revealed that TNNI induction leads to the overexpression of three genes strongly linked to tumorigenesis: insulin receptor (*InR*), Ras-related small GTPase protein 1 (*Rap1*), and insulin-like peptide 8 (*Dilp8*) [34, 275]. The expression of other proliferation-associated genes, including *Notch*, *Ras*, and *Myc*, was unaffected. These observations were subsequently extended to a mammalian model, in which TNNI1 is the closest homolog of the Drosophila *wupA* gene. Unsurprisingly, suppression of TNNI1 expression in non-small cell lung cancer (NSCLC) cells significantly reduced cancer growth compared to control cells [275]. Unfortunately, the mechanism by which TNN1 caused these changes was not explored. These results also suggest that TNNI1 selectively modulates gene expression without broadly activating other oncogenes. Based on these findings, the study's authors suggested that TNNI1 may function as a direct transcriptional regulator rather than as a part of an intranuclear motor protein complex, since less selective changes would be expected if TNNI1 acted alongside intranuclear actin to regulate RNA polymerase I activity [275, 276]. This conclusion is further supported by observations in non-cancerous cells [28, 137], where troponin I was shown to operate independently.

The effects of elevated TNNI2 expression on cancer cell growth are opposed to those of TNNI1. The angiogenesis-inhibiting effect of TNNI2 has already been discussed [31, 246]. However, TNNI2 also suppresses tumor growth by other mechanisms. Its overexpression increases apoptosis in SK-OV-3 ovarian carcinoma and MH3924A Morris hepatoma cells, and decreases their proliferation rate [35, 36], to a degree that cannot be explained solely by stress resulting from insufficient angiogenesis [35]. The mechanisms underlying the TNNI2 effect on cancer cell proliferation have not been further investigated. One possible pathway by which TNNI2 may affect cell proliferation is through disruption of bFGF signaling. Since bFGF signaling is known to support cancer cell proliferation by activating the Ras-MAPK and phosphatidylinositol 3-kinase/protein kinase B (PI3K/Akt) pathways [277–280], and TNNI2 has previously been shown to influence this signaling cascade [31, 246], this represents a plausible mechanism.

TNNI2 expression in hepatoma cells was associated with elevated expression of several pro-apoptotic factors, including ICE-like cysteine protease (caspase), leukocyte common antigen-related receptor (LAR), and the A2b adenosine receptor gene (ADORA2B). Analysis using an anti-p53 antibody further demonstrated that p53 levels were not elevated in TNNI2-expressing cells, suggesting the involvement of p53-independent mechanisms. These cells also exhibited markers of oxidative stress. However, the mechanism by which TNNI2 promotes pro-apoptotic signaling remains unknown [35].

An effective response to oxidative stress is a critical determinant of cell survival, and proteins involved in this process are known to activate apoptotic signaling when cellular defense mechanisms fail [281, 282]. One protein implicated in the oxidative stress response is the multifunctional nuclear receptor ERRα [283], which has previously been shown to interact with TNNI2 [221]. Depending on the cellular context, ERRα can either promote or suppress apoptotic signaling. In hepatocellular carcinoma, ERRα appears to enhance apoptosis and function as a tumor suppressor [284–286]. Further investigation of the interaction between TNNI2 and ERRα in hepatoma cells may therefore reveal a mechanistic link between TNNI2 expression and cancer cell apoptosis and provide new insights into the role of troponins in cancer biology.

Like the two previously discussed troponin variants, TNNI3 is expressed in cancer cells: it was detected in almost two-thirds of NSCLC cell lines that were examined: in squamous cell lung carcinoma, lung adenocarcinoma, and gastric adenocarcinoma. Moreover, it has been shown to exhibit nuclear localization in several cancer types [138]. Given the previously discussed regulatory role of TNNI1 in gene expression, it is plausible that TNNI3 also contributes to nuclear regulation in cancer cells.

The complex and sometimes contradictory roles of troponin I suggest that the documented interaction between TNNI3 and the YY1 protein may be important [137, 158]. The YY1 protein is known for its versatility in gene regulation, which results largely from its ability to interact with a wide range of binding partners [208]. Different TNNI isoforms could potentially modulate YY1's regulatory activity, which would be a plausible explanation for the functional diversity of troponin I. However, there is little evidence supporting this hypothesis at present: interaction databases such as IntAct [250] and Reactome [287] lack evidence linking YY1 to troponin I. Neither is the TNNI3/YY1 complex described. Understanding the precise structure of this complex is crucial for determining whether other troponin I isoforms could also bind to YY1. Overall, we are inclined to believe that troponin I can interact with multiple proteins involved in gene regulation, which may explain its versatility. However, this conclusion requires experimental validation, although existing evidence, including troponin I interactions with YY1 [137, 158], ERRα [221], and histone deacetylase 1 [28], as well as the presence of intrinsically disordered regions in the structure of troponin I [52] that enable conformational flexibility, supports this possibility.

### Context-dependent functions of troponin C

The case of TNNC1 highlights the critical role of the cellular context in determining the impact of changes in the expression of single genes. In many cases, TNNC1 acts as a proto-oncogene. For example, its expression correlates with poor prognosis in tongue cancer [288]. In hepatocellular carcinoma, TNNC1 supports cell migration. In these cells, TNNC1 overexpression results in increased phosphorylation of PI3K and AKT kinases and activation of their signaling pathway. TNNC1 knockdown has the opposite effect. Thus, TNNC1 likely supports cell migration in hepatocellular carcinoma by activating the PI3K/AKT signaling pathway [289].

TNNC1 also facilitates metastasis in gastric cancer by activating the transforming growth factor beta (TGF-β)/Smad pathway, as shown using TNNC1 silencing and by treating affected cells with TGF-β. TNNC1 silencing also induces apoptosis in these cells. E2F was identified as a transcription factor driving TNNC1 expression, with its binding sequences in the TNNC1 promoter described [273]. Similarly, in ovarian cancer, TNNC1 expression is associated with enhanced susceptibility to epithelial-mesenchymal transition (EMT) and enhanced cell motility [290, 291]. In A224, ALST, and OVCA432 cell lines, TNNC1 upregulation is driven by microfibril-associated protein 5 (MFAP5), which increases cancer cell motility through its effect on the calcium-dependent FAK/CREB/TNNC1 signaling pathway [291].

TNNC1 contributes to increased EMT and metastasis in SKOV-3–13 ovarian cancer cell line. Cells with TNNC1 knockout show decreased levels of phosphorylated AKT and phosphorylated Glycogen synthase kinase-3 beta (GSK-3β). This downregulation of AKT/GSK-3β signaling leads to reduced levels and nuclear localization of the transcription factors Snail and Slug [292–294]. In this case, TNNC1 overexpression is MFAP5-independent [290]. Interestingly, TNNC1 does not act as a protooncogene in lung adenocarcinoma cells, where low TNNC1 expression is associated with more aggressive cancer behavior and greater metastatic potential [140, 167]. It was also shown that TNNC1 levels correlate negatively with the activity of the KRAS pathway, which plays a key role in metastasis [140, 295].

It is tempting to speculate that the role of TNNC1 in cancer metastasis can be explained mechanistically by its interaction with other cytoskeletal proteins involved in cancer cell movement. However, migrating cancer cells are known to lack sarcomeric organization in their contractile machinery, and their cytoskeletal architecture more closely resembles that of smooth muscle cells. [296, 297]. In both cell types, the involvement of troponin in the contractile apparatus remains controversial and has not been conclusively demonstrated, although its expression in smooth muscle cells has been reported [298, 299]. Furthermore, troponin's primary role in regulating actomyosin contraction is performed by other proteins and regulatory networks in both cell types, making it functionally redundant [300–303]. Moreover, the studies cited above describe mechanisms by which TNNC1 can influence cancer cell motility without direct cytoskeletal involvement [273, 289, 290], with some studies also demonstrating restoration of TNNC1-induced metastatic potential following substitution of its known downstream targets in TNNC1 knockout cells [273]. Current evidence, therefore, suggests that TNNC1 involvement in cancer cell movement can be explained by its regulation of pro-oncogenic signaling pathways rather than by direct participation in the cytoskeleton.

Another notable observation is that DNA damage induces increased TNNC1 expression and nuclear localization. Given that cells exhibiting TNNC1 activity were halted from progressing into the G1 phase, it can be assumed that TNNC1 contributes to this response and hinders tumor progression. TNNC1, therefore, acts as a tumor suppressor in lung adenocarcinoma cells [140].

Similar downregulation has been reported for TNNC2 in head and neck squamous cell carcinoma cells [304, 305]. However, the effects of TNNC2 at the molecular level were not determined in this case, so we can only speculate that it might participate in a tumor suppressive cascade similar to that demonstrated for TNNC1.

### Troponin T promotes epithelial-mesenchymal transition

TNNT1 is expressed in several cancer cell lines [306]. In addition, the molecular mechanism by which TNNT1 overexpression contributes to the development of colorectal adenocarcinoma (COAD) has been characterized. Cells with elevated TNNI1 levels displayed enhanced proliferation together with corresponding increases in the expression of EMT-associated proteins, including N-cadherin, vimentin, matrix metalloproteinase MMP-9, and the transcription factor Snail. Conversely, expression of the EMT-inhibiting protein E-cadherin was reduced [307]. Similar effects linked to elevated troponin expression were observed in lung squamous cell carcinoma and lung adenocarcinoma. In these cases, the same EMT-related changes were seen, and a potential underlying mechanism was identified through TNNT1 knockdown [168].

**Fig. 5:**
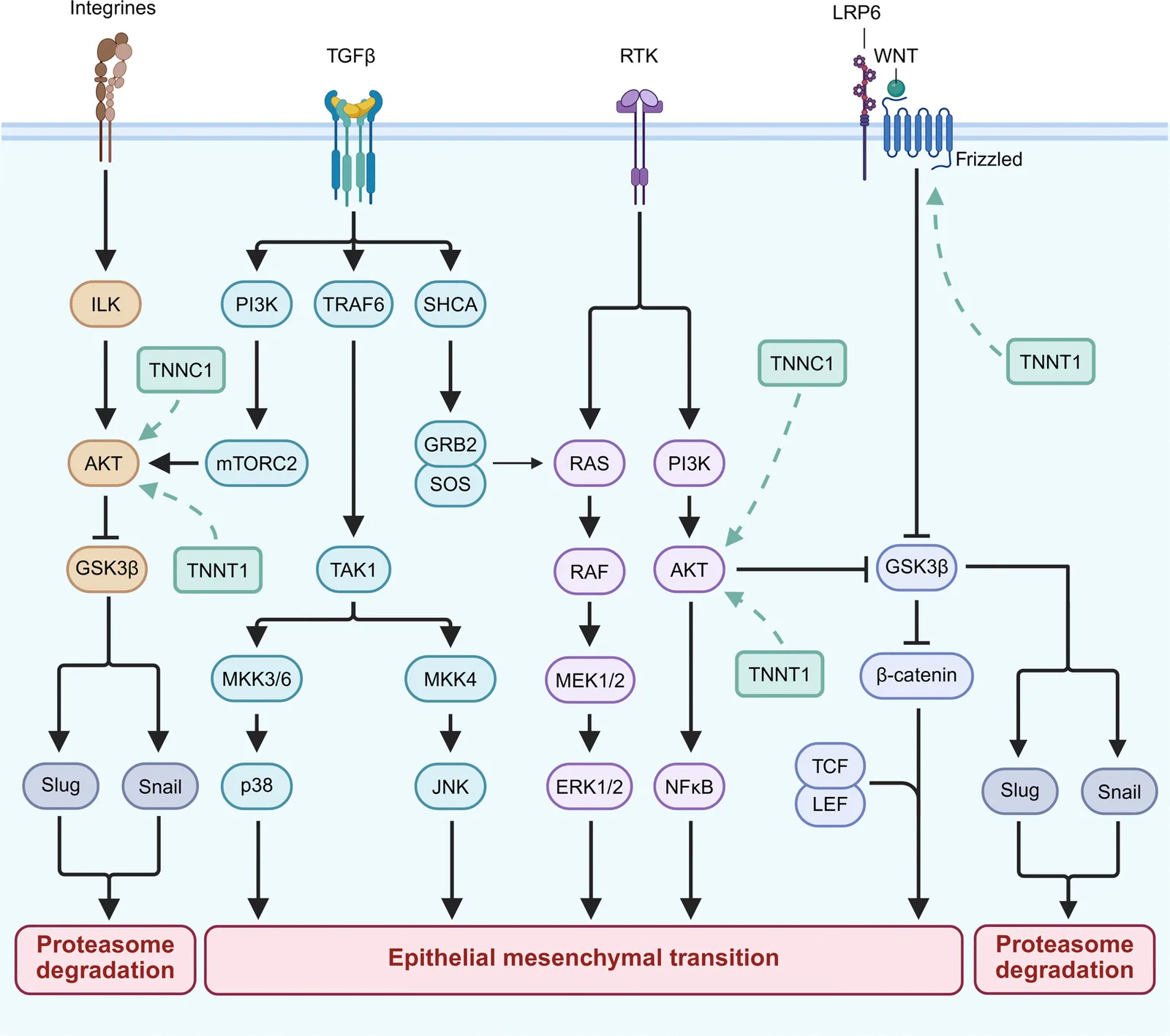
Troponin isoforms are involved in the regulation of EMT in cancer cells. Specifically, TNNC1 promotes EMT in ovarian cancer cells, whereas TNNT1 promotes EMT in colorectal carcinoma, lung squamous cell carcinoma and lung adenocarcinoma cells. EMT is regulated by several signaling pathways, including integrin, TGF-β, receptor tyrosine kinases, and Wnt signaling pathways. TNNC1 expression is associated with the phosphorylation of Akt and GSK-3β. Akt phosphorylates GSK-3β at serine 9, leading to its inactivation. In its active unphosphorylated state, GSK-3β suppresses EMT by phosphorylating transcription factors such as Snail and Slug, thereby targeting them for proteasomal degradation. GSK-3β is also inhibited by LRP6, a co-receptor in the Wnt/β-catenin signaling pathway. TNNT1 expression is associated with increased levels of several EMT-related proteins, including the transcription factor Snail. TNNT1 is presumed to promote this increase in Snail levels by affecting GSK-3β activity; however, the mechanism by which TNNT1 inhibits GSK-3β has not yet been elucidated with potential involvement of Wnt cascade being proposed.Abbreviations:EMT: epithelial-mesenchymal transition, TGF-β: transforming growth factor beta, GSK-3β: Glycogen synthase kinase-3 beta, LRP6: Low-density lipoprotein receptor-related protein 6

This knockdown resulted in a decrease in the levels of Snail, Slug, N-cadherin, and vimentin, and an increase in E-cadherin levels. Lithium chloride (LiCl), a known EMT activator [308–311], was used to treat TNNT1 knockdown cells, resulting in partial restoration of EMT. LiCl inhibits GSK-3β, which acts as a negative regulator of Snail and Slug proteins [293, 312, 313]. During EMT, GSK-3β is deactivated by phosphorylation at serine 9 by the Akt kinase, which is a downstream target of several EMT-related pathways, including the TGF-β and integrin-linked kinase (ILK) pathways. GSK-3β is also inhibited by low-density lipoprotein receptor-related protein 6 (LRP6), a protein involved in the Wnt/β-catenin pathway [314–319]. In this case, following LiCl treatment, levels of β-catenin and c-Myc proteins increased [168]. This is consistent with involvement of the Wnt/β-catenin pathway in TNNT1 function but does not exclude involvement of Akt kinase and its upstream activators. To further investigate the role of TNNT1 in EMT, it would be necessary to characterize changes in GSK-3β phosphorylation induced by TNNT1 downregulation, as well as levels of additional proteins more specific to the Wnt/β-catenin and Akt upstream activator pathways than β-catenin and c-Myc alone. Potential mechanisms by which troponin affects EMT are summarized in Fig. 5.

Another mechanism recently shown to contribute to tumorigenesis is miRNA-mediated regulation of expression [320]. One protein subject to this mode of regulation is troponin: miR-873 inhibited TNNT1 expression and suppressed its pro-oncogenic effect in colorectal cancer [321]. In silico analyses have revealed correlations between TNNT1 expression and the tumor microenvironment. Specifically, higher TNNT1 levels were associated with increased infiltration of dendritic cells and neutrophils, as well as a greater presence of tumor-associated fibroblasts within the tumor stroma [306]. However, these findings require further in vitro and in vivo validation. Another recently reported function of TNNT1 is the induction of paclitaxel resistance in human breast cancer cells. This likely results from activation of the RAS/RAF1 cascade by TNNT1, because the resistance was weakened by silencing TNNT1 and restored by treatment with a RAS/RAF1 cascade activator [322].

The roles of the other two TNNT isoforms in cancer are less well characterized. However, emerging evidence suggests their involvement in cancer development. For instance, TNNT2 has been detected in colorectal cancer cells, where its expression level correlated with poorer prognosis for patients [323].

At least in the case of TNNT1, there is evidence that troponin T participates in multiple oncogenesis-related pathways, highlighting its functional versatility. However, its precise mechanisms of action remain unclear. The studies discussed above focused on phenotypic and pathway-level changes in cancer cells without detailing the interactions between troponin T and its molecular targets. However, there have been some notable structural studies on TNNT that may offer useful insights. The presence of the LZD motif within the TNNT3 molecule has been experimentally confirmed (and the corresponding LZD motif has been predicted in other TNNT isoforms) [178], and its role in regulating gene expression has been established [157, 188]. Although this finding derives from non-oncological models, troponin T isoforms may influence tumorigenesis, at least in part through similar mechanisms.

Another important question regarding the role of troponins in cancer is the interaction among their different isoforms. Several studies have shown that multiple troponin isoforms are co-expressed in specific cancer types. For example, TNNI3, TNNC1, and TNNT1 are co-expressed in lung adenocarcinoma; TNNI2 and TNNC1 in ovarian and hepatocellular carcinoma [35, 36, 138, 140, 167, 168, 289–291]. Although these subunits do not normally form complexes in adult muscle tissues, some of them have been shown to interact during embryonic development and under pathological conditions [39, 94, 106, 112, 159–161, 163], raising the possibility that similar interactions may occur in cancer cells. Unfortunately, these potential interactions have not yet been investigated, and a detailed understanding of the mechanisms by which troponin isoforms influence cancer cell behavior remains lacking. For several isoforms, only correlations with favorable or unfavorable patient prognosis have been reported, while their underlying molecular mechanisms have not been explored [275, 304, 305, 323]. To summarize current knowledge, the roles of different troponin isoforms are presented in Table 2.

**Table 2:** Overview of troponin isoforms´ effects on tumorigenesis

Troponin subunit	Role in cancer	Notes	Cancer types	Key references
TNNI1	Promotes proliferation	Induces tumorigenic genes; drives growth	Non-small cell lung cancer	[275]
TNNI2	Suppresses tumor growth	Increases apoptosis; inhibits angiogenesis	Hepatoma; ovarian cancer	[35, 36]
TNNI3	Not well established	Nuclear presence	Lung squamous cell carcinoma; lung adenocarcinoma; gastric cancers	[138]
TNNC1	Proto-oncogene or tumor suppressor	Promotes metastasis or suppresses tumors based on context	Tongue; gastric cancer; hepatoma; lung adenocarcinoma	[140, 167, 273, 288–290]
TNNC2	Likely tumor suppressor	Reduced expression observed	Head and neck carcinoma	[304, 305]
TNNT1	Promotes EMT and drug resistance	increases EMT markers; increases proliferation; paclitaxel resistance	Colorectal; lung squamous cell carcinoma; lung adenocarcinoma; breast cancer	[168, 306, 307, 321, 322]

Further research is therefore required to determine whether troponin isoforms function independently in cancer cells or interact with one another. Given their frequently antagonistic effects on tumorigenesis, it is conceivable that interactions between troponin isoforms may inhibit their binding to downstream targets, thereby representing a potentially important tumor-suppressive mechanism.

## Clinical applications beyond myocardial infarction

### Non-cardiovascular diseases

Although cardiac troponins are primarily recognized as biomarkers of myocardial injury, increasing evidence indicates that elevated troponin concentrations are also frequently observed in non-cardiovascular diseases [324–327]. Conditions such as sepsis, chronic kidney disease, pulmonary embolism, stroke, severe respiratory infections, and systemic inflammatory disorders can induce secondary myocardial injury through mechanisms including hypoxia, oxidative stress, inflammation, microvascular dysfunction, and neurohormonal activation [324, 325, 328–335]. In these contexts, troponin elevation often reflects subclinical cardiomyocyte stress or systemic disease–associated myocardial involvement rather than acute coronary occlusion [324, 325, 328]. The adoption of high-sensitivity troponin assays has increased detection of low-level elevations in critically ill patients and highlights the complex relationship between systemic disease and cardiac injury [336–338].

From a molecular biomedicine perspective, troponin release in systemic diseases provides important insight into the interconnected nature of organ systems and cellular stress responses. Experimental studies suggest that inflammatory cytokines, mitochondrial dysfunction, endothelial injury, and apoptosis contribute to cardiomyocyte membrane instability and troponin release during systemic disease states [324, 325, 339, 340]. In addition, chronic renal dysfunction may impair troponin clearance and amplify circulating troponin concentrations [325, 329]. Troponin measurement, therefore, has prognostic value beyond cardiology: elevated levels in non-cardiovascular diseases are frequently associated with increased mortality, multiorgan dysfunction, and worse outcomes [341–344].

Future research integrating troponin biomarkers with proteomics, metabolomics, and inflammatory profiling may elucidate mechanisms of systemic myocardial injury and support precision medicine approaches in multisystem disorders.

### Troponin as a prognostic biomarker in cancer

Cardiac troponins have emerged as important prognostic biomarkers not only in cardiovascular disorders but also in cancer. In oncology, elevated troponin concentrations are predictive of chemotherapy- and radiotherapy-related cardiotoxicity, particularly in patients treated with anthracyclines, immune checkpoint inhibitors, HER2-targeted agents, or thoracic radiotherapy [17, 20, 23, 345–350]. High-sensitivity troponin assays can detect subclinical myocardial injury before cardiac dysfunction becomes apparent on imaging studies [337, 338]. Persistent or rising troponin levels during cancer therapy correlate strongly with left ventricular dysfunction, heart failure, and adverse cardiovascular outcomes [345, 346]. Consequently, troponin monitoring has become integral to cardio-oncology programs and enables early cardioprotective interventions [351–353].

Unlike in myocardial infarction, no diagnostic window for troponin has been established in cancer therapy-related cardiotoxicity; therefore, the optimal timing of blood sampling remains unknown. Adamcova et al. used a rabbit model to examine daunorubicin-induced cardiotoxicity. In this study, the plasma levels of cardiac troponins progressively increased with the rising number of chemotherapy cycles. A significant rise in cTnT and cTnI was observed after the fifth and eighth administrations. Two hours after these administrations, troponin levels increased significantly, peaked between 4 and 6 h, and declined over the following 24 h. Low-level troponin release persisted even after cessation of therapy [20].

In a prospective study of patients with breast cancer treated with anthracyclines and trastuzumab, TNNT2 levels peaked one month after treatment; however, these peak levels were not predictive of clinical outcomes [354]. Other studies investigating anthracycline- and trastuzumab-induced cardiotoxicity similarly reported only an association between elevated troponin levels and poorer clinical outcomes compared with patients who maintained normal troponin levels, without determining the predictive value of different troponin levels [26, 355–357].

Establishing clinically relevant troponin cut-off values that predict cancer therapy-induced cardiotoxicity would enable more effective monitoring of patients and could potentially improve clinical outcomes and survival.

### Potential clinical applications

Although troponin has been implicated in the pathogenesis of a wide range of diseases, and multiple non-classical mechanisms by which it modulates pathological cell signaling have been identified, these findings have not yet been translated into clinical research or routine clinical practice. As a result, troponin remains primarily utilized as a biomarker of cardiac injury. Nevertheless, evidence from basic research highlights several emerging opportunities for the development of clinical applications that target the non-classical functions of troponin.

Changes in the structure of troponin are primary drivers of several cardiomyopathies. The pathogenetic processes underlying the development of these conditions have traditionally been explained by alterations in excitation–contraction coupling caused by an altered troponin response to calcium binding. However, novel studies have shown that mutant troponin variants are also implicated in epigenetic regulation through interactions with histone deacetylases and demethylases [29, 236], as well as in the disruption of mitochondrial metabolism [249]. Nevertheless, an important question remains: how significant are these pathways in the pathogenesis of cardiomyopathies, and can they be therapeutically targeted? One possible approach to correcting troponin-mediated intranuclear signaling is to focus on its downstream targets. Inhibitors of histone deacetylases and demethylases are currently being investigated in both non-cardiac and cardiac diseases, with some already being used in cancer therapy [358–361]. In addition to epigenetic enzyme inhibitors, therapies targeting phosphodiesterases in cardiac disease have already been implemented [362, 363]. Given that PDE2A, PDE3A, and PDE4D are putative downstream targets of troponin [30, 236], their efficacy in troponin mutation–driven cardiomyopathies merits preclinical evaluation.

Overexpression of troponin in non-cardiac cells has been shown to affect tumorigenesis [35, 168, 289, 291]. In addition to its potential use as a molecular prognostic marker [140, 167, 288, 304, 305], troponin may also represent a therapeutic target. However, a key limitation in targeting troponin in cancer lies in its essential role in muscle physiology. Targeting tissue-specific troponin isoforms would therefore likely be impractical due to significant off-target effects. One potential way to overcome this limitation is to target troponin variants that differ structurally from their tissue-specific counterparts, such as mutant or re-expressed fetal isoforms. However, it remains unknown whether such variants are expressed in cancer cells, representing a significant knowledge gap that warrants further investigation.

Another potential strategy is to target upstream or downstream components of troponin signaling. However, troponin’s position within the cancer cellular network is only partially understood, making it currently difficult to evaluate potential drug targets within this pathway. Troponin isoform expression levels also differ across cancer types, and their tumorigenic or tumor-suppressive roles appear to be context-dependent [288–291]. Identifying the factors that determine these differences, particularly those that promote the expression of tumor-suppressive troponin isoforms, may offer additional therapeutic opportunities.

Troponin isoforms might also not form complete complexes inside cancer cells and instead interact with binding partners in monomeric form. Such monomeric troponins might be targeted by drugs specifically designed to bind regions of troponin that are normally hidden through interactions with other subunits of the troponin complex. This represents a speculative therapeutic approach, but it could allow for targeting troponin in cancer without significantly affecting muscle physiology, given that troponin predominantly exists in its complex form in these cells [257, 258]. Whether this potential therapeutic strategy is viable (for example, monomeric troponin may play an important role in physiological intranuclear signaling in muscle cells) remains to be determined by future research.

One potential therapeutic approach targeting troponin without affecting healthy muscle tissue is the use of cold atmospheric plasma (CAP). CAP is a near-room-temperature, partially ionized gas that destroys target cells primarily through oxidative stress-induced apoptosis [364, 365]. When applied to cancer cell tissue, CAP has been shown to selectively kill cancer cells, presumably because these cells exhibit higher basal levels of reactive oxygen species [366]. Calcium and calcium signaling appear to play a significant role in CAP-induced apoptosis. Not only is this apoptotic process calcium-dependent, but CAP also induces calcium influx into cells [364, 365, 367, 368]. Given that troponin is involved in cellular calcium signaling [363, 364] and influences processes in cancer cells, including apoptosis [35, 36], it may be one of the proteins contributing to the cancer cell-specific effects of CAP.

Using the CAPmed-BC database [369], we conducted a preliminary analysis of CAP effects on troponin expression in triple-negative breast cancer (TNBC) cells [370, 371]. Among the analyzed troponin isoforms, TNNC2 exhibited a log2-fold change in expression of 0.41 when comparing untreated TNBC cells with TNBC cells 1 h after CAP treatment. TNNT1 and TNNI2 showed log2-fold changes in expression of 0.72 and 0.24, respectively, when comparing untreated TNBC cells with TNBC cells 8 h after CAP treatment. All comparisons were performed using a significance level of 0.05. The functional roles of these isoforms in breast cancer apoptosis have not been established; therefore, the biological significance of these expression changes remains uncertain. Nonetheless, these findings suggest that CAP can modulate troponin expression and justify targeted follow-up studies to determine the causal relationships and functional consequences of these changes.

## Outstanding questions and future perspectives

There is now a substantial body of evidence indicating that troponin performs functions beyond its classic role in regulating actin-myosin interaction. Outside the nucleus, it influences mitochondrial metabolism and participates in angiogenesis inhibition. It plays a role in many intranuclear processes, acts as a transcription factor, and influences epigenetic regulation by modulating the activity of methyltransferases and deacetylases. Many of these functions influence tumorigenesis, where troponin intriguingly serves both as a tumor suppressor and an oncogene depending on the circumstances. Despite extensive research on troponin’s non-traditional roles, many questions remain unanswered, and we are only beginning to unravel them.

First, the relationship between troponin and the nucleoskeleton remains unresolved. Although troponin localizes to the nucleus [133–140] and performs distinct nuclear functions [157, 188, 221, 236], it has not been established whether these functions are independent or mediated through incorporation into the nucleoskeleton. Several independent nuclear functions of troponin have been documented [28, 29, 156–158]; however, whether these functions require interactions with the nucleoskeleton remains unknown. Other cytoskeletal proteins that localize to the nucleus can function both independently [151–155] and as components of the nucleoskeleton [142, 146–150]. Determining whether the same is true for troponin will require evidence of its stable association with the nucleoskeleton.

Second, it is unknown whether troponin subunits that co-localize in the nucleus assemble into intranuclear complexes, and if so, whether these are identical to the tissue-specific heterotrimers found in the cytoplasm or represent heterogeneous assemblies of different isoforms. Formation of heterogeneous complexes has been experimentally demonstrated for some isoforms [112, 164–166], so defining the composition and functional relevance of nuclear troponin complexes is a priority.

Third, several troponin isoforms contain predicted nuclear localization signals (NLS) and leucine zipper–like domains (LZD) [136, 178, 189, 190], but the functionality of many of these motifs remains unvalidated. Notably, the confirmed NLS in TNNT3 differed from in silico predictions [178], underscoring the need for experimental testing of NLS/LZD activity across isoforms to provide mechanistic insight into troponin nuclear import and protein–protein interactions.

Fourth, although troponin influences transcription, epigenetic modification, Ca^2^⁺ signaling outside the sarcomere, and mitochondrial function [30, 157, 188, 221, 236, 249], how these effects integrate into broader signaling networks is poorly understood. Elucidating upstream regulators, downstream effectors, and the crosstalk between these pathways will clarify troponin’s cellular roles.

Fifth, isoform-specific functions merit deeper study. For example, TNNI2 inhibits angiogenesis and is implicated in tumor suppression, but few studies have explored how its anti-angiogenic activity integrates with other tumor-suppressive mechanisms [31, 35, 36, 246]. Similarly, TNNI3 inhibits mitochondrial ATP synthase and increases mitochondrial membrane permeability to promote pro-apoptotic signaling [30]; given the centrality of mitochondrial dysfunction in cancer and precedent for other ATP synthase inhibitors in tumorigenesis [260–265, 267, 268], the impact of TNNI3 on cancer cell bioenergetics and survival warrants focused investigation.

**Fig. 6:**
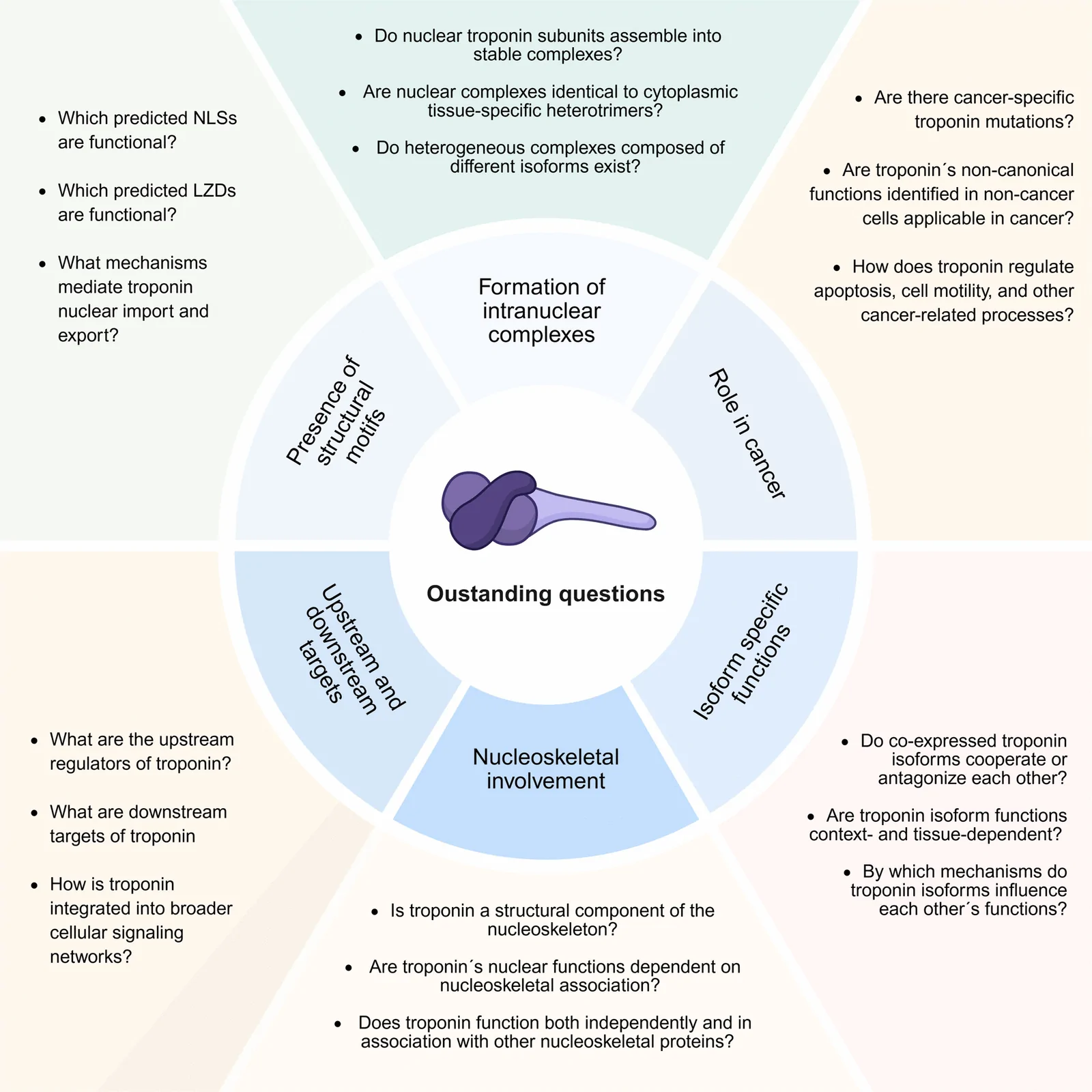
Outstanding questions regarding the non-classical functions of troponin. This figure provides an overview of the most important unanswered questions, the answers to which would substantially advance our understanding of troponin's cellular roles under both physiological and pathological conditions. Abbreviations: NLS: nuclear localization signal, LZD: leucine zipper domain

Finally, although multiple troponin isoforms have been associated with cancer progression, important mechanistic questions remain. The molecular basis for troponin-regulated apoptosis and motility has been partially characterized [35, 36, 168, 290, 291, 307], but validation, identification of direct binding partners, and mapping of signaling cascades are required. It is also unclear whether interactions observed between troponin and epigenetic or transcriptional regulators in non-cancer contexts [157, 188, 221, 236] contribute to tumorigenesis. Crucially, co-expression of multiple isoforms within the same tumors [35, 36, 138, 140, 167, 168, 289–291] raises the question of whether isoforms cooperate, antagonize one another, or function independently-knowledge that could reveal novel mechanisms of tumor suppression or progression and identify cancer-specific troponin alterations amenable to therapeutic targeting. Outstanding questions regarding troponin´s non-classical functions are summarized in Fig. 6.

Addressing these questions will require integrated approaches combining high-resolution imaging, proteomics, functional genomics, and in vivo models to define troponin’s context-dependent molecular architecture and signaling roles in health and disease.

## Conclusion

The evidence presented in this review demonstrates that troponin should no longer be viewed merely as a structural component of the contractile apparatus or a biomarker of cardiac injury. Instead, it is emerging as a multifunctional regulator involved in transcriptional and epigenetic regulation, mitochondrial metabolism, calcium signaling, and angiogenesis. These non-canonical functions establish mechanistic links between troponin and fundamental cellular processes, including proliferation, apoptosis, and tumor progression.

This evolving perspective significantly broadens the current understanding of troponin biology and highlights its potential as both a diagnostic biomarker and a therapeutic target across cardiovascular disease and cancer. Further investigation of these unconventional roles will not only clarify the physiological and pathological significance of troponin but may also reveal new opportunities for precision diagnostics and targeted therapies.

## Data Availability

The data that support the findings of this study are available in Uniprot database (https://www.uniprot.org), Protein Data Bank in Europe (https://www.ebi.ac.uk/pdbe/), Expression Atlas database (https://www.ebi.ac.uk/gxa/home), Vesiclepedia database (www.microvesicles.org), IntAct database (https://www.ebi.ac.uk/intact/home), Reactome database (https://reactome.org) and CAPmed-BC database (https://capbc.hiplot.com.cn/). Data were downloaded between August and September 2025 and, in the case of the CAPmed-BC database, in June 2026.

## References

[1] Ebashi S. Third component participating in the superprecipitation of “natural actomyosin.” Nature. 1963;200:1010. 10.1038/2001010a0. 14097720

[2] Ebashi S, Ebashi F. A new protein component participating in the superprecipitation of myosin B. J Biochem. 1964;55:604–13. 10.1093/oxfordjournals.jbchem.a127933. 14216404

[3] Perry SV, Grand RJ, Nairn AC, Vanaman TC, Wall CM. Calcium-binding proteins and the regulation of contractile activity. Biochem Soc Trans. 1979;7(4):619–22. 10.1042/bst0070619. 383543

[4] Gomes AV, Potter JD, Szczesna-Cordary D. The role of troponins in muscle contraction. IUBMB Life. 2002;54(6):323–33. 10.1080/15216540216037. 12665242

[5] Perry SV. Calcium ions and the function of the contractile proteins of muscle. Biochem Soc Symp. 1974;39:115–32. 4220103

[6] Park KC, Gaze DC, Collinson PO, Marber MS. Cardiac troponins: from myocardial infarction to chronic disease. Cardiovasc Res. 2017;113(14):1708–18. 10.1093/cvr/cvx183. PMC5852618 29016754

[7] Bodor GS, Porter S, Landt Y, Ladenson JH. Development of monoclonal antibodies for an assay of cardiac troponin-I and preliminary results in suspected cases of myocardial infarction. Clin Chem. 1992;38(11):2203–14. 1424112

[8] Alpert JS, Thygesen K, Antman E, Bassand JP. Myocardial infarction redefined–a consensus document of The Joint European Society of Cardiology/American College of Cardiology Committee for the redefinition of myocardial infarction. J Am Coll Cardiol. 2000;36(3):959–69. 10.1016/s0735-1097(00)00804-4. 10987628

[9] Apple FS, Collinson PO, Biomarkers ITFoCAoC. Analytical characteristics of high-sensitivity cardiac troponin assays. Clin Chem. 2012;58(1):54–61. 10.1373/clinchem.2011.165795. 21965555

[10] Apple FS, Wu AH, Jaffe AS. European Society of Cardiology and American College of Cardiology guidelines for redefinition of myocardial infarction: how to use existing assays clinically and for clinical trials. Am Heart J. 2002;144(6):981–6. 10.1067/mhj.2002.124048. 12486421

[11] Katus HA, Looser S, Hallermayer K, Remppis A, Scheffold T, Borgya A, et al. Development and in vitro characterization of a new immunoassay of cardiac troponin T. Clin Chem. 1992;38(3):386–93. 1547556

[12] Collinson PO, Saenger AK, Apple FS, Ifcc CC. High sensitivity, contemporary and point-of-care cardiac troponin assays: educational aids developed by the IFCC Committee on Clinical Application of Cardiac Bio-Markers. Clin Chem Lab Med. 2019;57(5):623–32. 10.1515/cclm-2018-1211. 30530880

[13] Sandoval Y, Jaffe AS. Using high-sensitivity cardiac troponin T for acute cardiac care. Am J Med. 2017;130(12):1358-65 e1. 10.1016/j.amjmed.2017.07.033. 28843652

[14] Gulati M, Levy PD, Mukherjee D, Amsterdam E, Bhatt DL, Birtcher KK, et al. 2021 AHA/ACC/ASE/CHEST/SAEM/SCCT/SCMR guideline for the evaluation and diagnosis of chest pain: a report of the American College of Cardiology/American Heart Association Joint Committee on Clinical Practice Guidelines. Circulation. 2021;144(22):e368–454. 10.1161/CIR.0000000000001029. 34709879

[15] Thygesen K, Alpert JS, Jaffe AS, Chaitman BR, Bax JJ, Morrow DA, et al. Fourth universal definition of myocardial infarction (2018). J Am Coll Cardiol. 2018;72(18):2231–64. 10.1016/j.jacc.2018.08.1038. 30153967

[16] Sandoval Y, Jaffe AS. Type 2 myocardial infarction: JACC review topic of the week. J Am Coll Cardiol. 2019;73(14):1846–60. 10.1016/j.jacc.2019.02.018. 30975302

[17] Adamcova M, Gersl V, Hrdina R, Melka M, Mazurova Y, Vavrova J, et al. Cardiac troponin T as a marker of myocardial damage caused by antineoplastic drugs in rabbits. J Cancer Res Clin Oncol. 1999;125(5):268–74. 10.1007/s004320050273. PMC12199899 10359131

[18] Simunek T, Klimtova I, Adamcova M, Gersl V, Hrdina R, Sterba M, et al. Cardiac troponin T as an indicator of reduced left ventricular contractility in experimental anthracycline-induced cardiomyopathy. Cancer Chemother Pharmacol. 2003;52(5):431–4. 10.1007/s00280-003-0675-z. 14618274

[19] Adamcova M, Simunek T, Kaiserova H, Popelova O, Sterba M, Potacova A, et al. In vitro and in vivo examination of cardiac troponins as biochemical markers of drug-induced cardiotoxicity. Toxicology. 2007;237(1–3):218–28. 10.1016/j.tox.2007.05.016. 17587482

[20] Adamcova M, Lencova-Popelova O, Jirkovsky E, Mazurova Y, Palicka V, Simko F, et al. Experimental determination of diagnostic window of cardiac troponins in the development of chronic anthracycline cardiotoxicity and estimation of its predictive value. Int J Cardiol. 2015;201:358–67. 10.1016/j.ijcard.2015.07.103. 26310978

[21] Jirkovsky E, Jirkovska A, Bavlovic-Piskackova H, Skalicka V, Pokorna Z, Karabanovich G, et al. Clinically translatable prevention of anthracycline cardiotoxicity by dexrazoxane is mediated by topoisomerase II beta and not metal chelation. Circ Heart Fail. 2021;14(11):e008209. 10.1161/CIRCHEARTFAILURE.120.008209. 34551586

[22] Adamcova M, Sterba M, Simunek T, Potacova A, Popelova O, Gersl V. Myocardial regulatory proteins and heart failure. Eur J Heart Fail. 2006;8(4):333–42. 10.1016/j.ejheart.2005.09.007. 16309957

[23] Pudil R, Mueller C, Celutkiene J, Henriksen PA, Lenihan D, Dent S, et al. Role of serum biomarkers in cancer patients receiving cardiotoxic cancer therapies: a position statement from the Cardio-Oncology Study Group of the Heart Failure Association and the Cardio-Oncology Council of the European Society of Cardiology. Eur J Heart Fail. 2020;22(11):1966–83. 10.1002/ejhf.2017. 33006257

[24] Adamcova M, Sterba M, Simunek T, Potacova A, Popelova O, Mazurova Y, et al. Troponin as a marker of myocardiac damage in drug-induced cardiotoxicity. Expert Opin Drug Saf. 2005;4(3):457–72. 10.1517/14740338.4.3.457. 15934853

[25] Armenian SH, Lacchetti C, Barac A, Carver J, Constine LS, Denduluri N, et al. Prevention and monitoring of cardiac dysfunction in survivors of adult cancers: American Society of Clinical Oncology Clinical Practice Guideline. J Clin Oncol. 2017;35(8):893–911. 10.1200/JCO.2016.70.5400. 27918725

[26] Michel L, Mincu RI, Mahabadi AA, Settelmeier S, Al-Rashid F, Rassaf T, et al. Troponins and brain natriuretic peptides for the prediction of cardiotoxicity in cancer patients: a meta-analysis. Eur J Heart Fail. 2020;22(2):350–61. 10.1002/ejhf.1631. 31721381

[27] Lyon AR, Lopez-Fernandez T, Couch LS, Asteggiano R, Aznar MC, Bergler-Klein J, et al. 2022 ESC Guidelines on cardio-oncology developed in collaboration with the European Hematology Association (EHA), the European Society for Therapeutic Radiology and Oncology (ESTRO) and the International Cardio-Oncology Society (IC-OS). Eur Heart J. 2022;43(41):4229–361. 10.1093/eurheartj/ehac244. 36017568

[28] Zhao W, Wu X, Wang Z, Pan B, Liu L, Liu L, et al. Epigenetic regulation of phosphodiesterase 4d in restrictive cardiomyopathy mice with cTnI mutations. Sci China Life Sci. 2020;63(4):563–70. 10.1007/s11427-018-9463-9. 30900165

[29] Zhao W, Qian L, Luo J, Pan B, Liu LJ, Tian J. Cardiac troponin I R193H mutant interacts with HDAC1 to repress phosphodiesterase 4D expression in cardiomyocytes. Genes Dis. 2021;8(4):569–79. 10.1016/j.gendis.2020.01.004. PMC8209310 34179318

[30] Elezaby A, Lin AJ, Vijayan V, Pokhrel S, Kraemer BR, Bechara LRG, et al. Cardiac troponin I directly binds and inhibits mitochondrial ATP synthase with a noncanonical role in the post-ischemic heart. Nat Cardiovasc Res. 2024;3(8):987–1002. 10.1038/s44161-024-00512-1. PMC11700703 39196031

[31] Kern BE, Balcom JH, Antoniu BA, Warshaw AL, Fernandez-del Castillo C. Troponin I peptide (Glu94-Leu123), a cartilage-derived angiogenesis inhibitor: in vitro and in vivo effects on human endothelial cells and on pancreatic cancer. J Gastrointest Surg. 2003;7(8):961–8. 10.1016/j.gassur.2003.08.003. 14675705

[32] Johnston JR, Chase PB, Pinto JR. Troponin through the looking-glass: emerging roles beyond regulation of striated muscle contraction. Oncotarget. 2018;9(1):1461–82. 10.18632/oncotarget.22879. PMC5787451 29416706

[33] Rezazadeh M, Danaei-Mehrabad S, Afshar Zakariya N, Kazemi F, Moslehian MS, Tamadon A, et al. Identification of key pathways and molecular players potentially involved in endometrial cancer metastasis through integrated bioinformatics analyses. Bioimpacts. 2025;15:30222. 10.34172/bi.30222. PMC11954752 40161945

[34] Garelli A, Gontijo AM, Miguela V, Caparros E, Dominguez M. Imaginal discs secrete insulin-like peptide 8 to mediate plasticity of growth and maturation. Science. 2012;336(6081):579–82. 10.1126/science.1216735. 22556250

[35] Schmidt K, Hoffend J, Altmann A, Kiessling F, Strauss L, Koczan D, et al. Troponin I overexpression inhibits tumor growth, perfusion, and vascularization of morris hepatoma. J Nucl Med. 2006;47(9):1506–14. 16954560

[36] Xiong G, Yang L, Wei Y, Wang S, Tian L, Lei S, et al. Expression of the human fast-twitch skeletal muscle troponin I cDNA in a human ovarian carcinoma suppresses tumor growth. Sci China C Life Sci. 2007;50(1):93–100. 10.1007/s11427-007-2032-7. 17393089

[37] Gokhan I, Dong W, Grubman D, Mezue K, Yang D, Wang Y, et al. Clinical biochemistry of serum troponin. Diagnostics (Basel). 2024;14(4). 10.3390/diagnostics14040378. PMC10887818 38396417

[38] Ragusa R, Caselli C. Focus on cardiac troponin complex: from gene expression to cardiomyopathy. Genes Dis. 2024;11(6):101263. 10.1016/j.gendis.2024.101263. PMC11357864 39211905

[39] Wei B, Jin JP. TNNT1, TNNT2, and TNNT3: isoform genes, regulation, and structure-function relationships. Gene. 2016;582(1):1–13. 10.1016/j.gene.2016.01.006. PMC5325693 26774798

[40] Wei B, Jin JP. Troponin T isoforms and posttranscriptional modifications: evolution, regulation and function. Arch Biochem Biophys. 2011;505(2):144–54. 10.1016/j.abb.2010.10.013. PMC3018564 20965144

[41] Solaro RJ, Rosevear P, Kobayashi T. The unique functions of cardiac troponin I in the control of cardiac muscle contraction and relaxation. Biochem Biophys Res Commun. 2008;369(1):82–7. 10.1016/j.bbrc.2007.12.114. PMC2365727 18162178

[42] Sheng JJ, Jin JP. TNNI1, TNNI2 and TNNI3: evolution, regulation, and protein structure-function relationships. Gene. 2016;576(1 Pt 3):385–94. 10.1016/j.gene.2015.10.052. PMC5798203 26526134

[43] Reinoso TR, Landim-Vieira M, Shi Y, Johnston JR, Chase PB, Parvatiyar MS, et al. A comprehensive guide to genetic variants and post-translational modifications of cardiac troponin C. J Muscle Res Cell Motil. 2021;42(2):323–42. 10.1007/s10974-020-09592-5. PMC8110610 33179204

[44] Lu QW, Wu XY, Morimoto S. Inherited cardiomyopathies caused by troponin mutations. J Geriatr Cardiol. 2013;10(1):91–101. 10.3969/j.issn.1671-5411.2013.01.014. PMC3627712 23610579

[45] Messer AE, Jacques AM, Marston SB. Troponin phosphorylation and regulatory function in human heart muscle: dephosphorylation of Ser23/24 on troponin I could account for the contractile defect in end-stage heart failure. J Mol Cell Cardiol. 2007;42(1):247–59. 10.1016/j.yjmcc.2006.08.017. 17081561

[46] Wijnker PJ, Murphy AM, Stienen GJ, van der Velden J. Troponin I phosphorylation in human myocardium in health and disease. Neth Heart J. 2014;22(10):463–9. 10.1007/s12471-014-0590-4. PMC4188840 25200323

[47] Bodor GS, Oakeley AE, Allen PD, Crimmins DL, Ladenson JH, Anderson PA. Troponin I phosphorylation in the normal and failing adult human heart. Circulation. 1997;96(5):1495–500. 10.1161/01.cir.96.5.1495. 9315537

[48] Bowling N, Walsh RA, Song G, Estridge T, Sandusky GE, Fouts RL, et al. Increased protein kinase C activity and expression of Ca2+-sensitive isoforms in the failing human heart. Circulation. 1999;99(3):384–91. 10.1161/01.cir.99.3.384. 9918525

[49] Zhang R, Zhao J, Potter JD. Phosphorylation of both serine residues in cardiac troponin I is required to decrease the Ca2+ affinity of cardiac troponin C. J Biol Chem. 1995;270(51):30773–80. 10.1074/jbc.270.51.30773. 8530519

[50] Li MX, Hwang PM. Structure and function of cardiac troponin C (TNNC1): implications for heart failure, cardiomyopathies, and troponin modulating drugs. Gene. 2015;571(2):153–66. 10.1016/j.gene.2015.07.074. PMC4567495 26232335

[51] Rasmussen M, Jin JP. Troponin variants as markers of skeletal muscle health and diseases. Front Physiol. 2021;12:747214. 10.3389/fphys.2021.747214. PMC8559874 34733179

[52] Marston S, Zamora JE. Troponin structure and function: a view of recent progress. J Muscle Res Cell Motil. 2020;41(1):71–89. 10.1007/s10974-019-09513-1. PMC7109197 31030382

[53] Tanokura M, Tawada Y, Ono A, Ohtsuki I. Chymotryptic subfragments of troponin T from rabbit skeletal muscle. Interaction with tropomyosin, troponin I and troponin C. J Biochem. 1983;93(2):331–7. 10.1093/oxfordjournals.jbchem.a134185. 6841341

[54] Perry SV. Troponin T: genetics, properties and function. J Muscle Res Cell Motil. 1998;19(6):575–602. 10.1023/a:1005397501968. 9742444

[55] Sheng JJ, Jin JP. Gene regulation, alternative splicing, and posttranslational modification of troponin subunits in cardiac development and adaptation: a focused review. Front Physiol. 2014;5:165. 10.3389/fphys.2014.00165. PMC4012202 24817852

[56] Larsson L, Wang X, Yu F, Hook P, Borg K, Chong SM, et al. Adaptation by alternative RNA splicing of slow troponin T isoforms in type 1 but not type 2 Charcot-Marie-Tooth disease. Am J Physiol Cell Physiol. 2008;295(3):C722–31. 10.1152/ajpcell.00110.2008. PMC2544436 18579801

[57] Jin JP, Lin JJ. Rapid purification of mammalian cardiac troponin T and its isoform switching in rat hearts during development. J Biol Chem. 1988;263(15):7309–15. 3366782

[58] Amarasinghe C, Jin JP. N-terminal hypervariable region of muscle type isoforms of troponin T differentially modulates the affinity of tropomyosin-binding site 1. Biochemistry. 2015;54(24):3822–30. 10.1021/acs.biochem.5b00348. 26024675

[59] Jin JP, Chong SM. Localization of the two tropomyosin-binding sites of troponin T. Arch Biochem Biophys. 2010;500(2):144–50. 10.1016/j.abb.2010.06.001. PMC2904419 20529660

[60] Pavadai E, Rynkiewicz MJ, Ghosh A, Lehman W. Docking troponin T onto the tropomyosin overlapping domain of thin filaments. Biophys J. 2020;118(2):325–36. 10.1016/j.bpj.2019.11.3393. PMC6976810 31864661

[61] Zamora JE, Papadaki M, Messer AE, Marston SB, Gould IR. Troponin structure: its modulation by Ca(2+) and phosphorylation studied by molecular dynamics simulations. Phys Chem Chem Phys. 2016;18(30):20691–707. 10.1039/c6cp02610a. 27412261

[62] Armstrong DR, Berrisford JM, Conroy MJ, Gutmanas A, Anyango S, Choudhary P, et al. PDBe: improved findability of macromolecular structure data in the PDB. Nucleic Acids Res. 2020;48(D1):D335–43. 10.1093/nar/gkz990. PMC7145656 31691821

[63] Meng EC, Goddard TD, Pettersen EF, Couch GS, Pearson ZJ, Morris JH, et al. UCSF ChimeraX: tools for structure building and analysis. Protein Sci. 2023;32(11):e4792. 10.1002/pro.4792. PMC10588335 37774136

[64] Takeda S, Yamashita A, Maeda K, Maeda Y. Structure of the core domain of human cardiac troponin in the Ca(2+)-saturated form. Nature. 2003;424(6944):35–41. 10.1038/nature01780. 12840750

[65] Wang Z, Raunser S. Structural biochemistry of muscle contraction. Annu Rev Biochem. 2023;92:411–33. 10.1146/annurev-biochem-052521-042909. 37001141

[66] Han D, Lim Y, Lee S, Eyun SI. Troponin I - a comprehensive review of its function, structure, evolution, and role in muscle diseases. Anim Cells Syst (Seoul). 2025;29(1):446–68. 10.1080/19768354.2025.2533821. PMC12305882 40735528

[67] Zhang Z, Akhter S, Mottl S, Jin JP. Calcium-regulated conformational change in the C-terminal end segment of troponin I and its binding to tropomyosin. FEBS J. 2011;278(18):3348–59. 10.1111/j.1742-4658.2011.08250.x. PMC3168705 21777381

[68] Akhter S, Bueltmann K Jr., Huang X, Jin JP. Restrictive cardiomyopathy mutations demonstrate functions of the C-terminal end-segment of troponin I. Arch Biochem Biophys. 2014;552–553:3–10. 10.1016/j.abb.2013.12.001. 24326031

[69] Yamada Y, Namba K, Fujii T. Cardiac muscle thin filament structures reveal calcium regulatory mechanism. Nat Commun. 2020;11(1):153. 10.1038/s41467-019-14008-1. PMC6952405 31919429

[70] Gillis TE, Marshall CR, Tibbits GF. Functional and evolutionary relationships of troponin C. Physiol Genomics. 2007;32(1):16–27. 10.1152/physiolgenomics.00197.2007. 17940202

[71] Rayani K, Seffernick J, Li AY, Davis JP, Spuches AM, Van Petegem F, et al. Binding of calcium and magnesium to human cardiac troponin C. J Biol Chem. 2021;296:100350. 10.1016/j.jbc.2021.100350. PMC7961095 33548225

[72] Aborode AT, Olamilekan Adesola R, Idris I, Adio WS, Scott GY, Chakoma M, et al. Troponin C gene mutations on cardiac muscle cell and skeletal regulation: a comprehensive review. Gene. 2024;927:148651. 10.1016/j.gene.2024.148651. 38871035

[73] Vinogradova MV, Stone DB, Malanina GG, Karatzaferi C, Cooke R, Mendelson RA, et al. Ca(2+)-regulated structural changes in troponin. Proc Natl Acad Sci U S A. 2005;102(14):5038–43. 10.1073/pnas.0408882102. PMC555973 15784741

[74] Tobacman LS. Troponin revealed: uncovering the structure of the thin filament on-off switch in striated muscle. Biophys J. 2021;120(1):1–9. 10.1016/j.bpj.2020.11.014. PMC7820733 33221250

[75] Lehman W, Rynkiewicz MJ. Troponin-I-induced tropomyosin pivoting defines thin-filament function in relaxed and active muscle. J Gen Physiol. 2023;155(7). 10.1085/jgp.202313387. PMC10227645 37249525

[76] Risi CM, Belknap B, Atherton J, Coscarella IL, White HD, Bryant Chase P, et al. Troponin structural dynamics in the native cardiac thin filament revealed by cryo electron microscopy. J Mol Biol. 2024;436(6):168498. 10.1016/j.jmb.2024.168498. PMC11007730 38387550

[77] Sevrieva IR, Kampourakis T, Irving M. Structural changes in troponin during activation of skeletal and heart muscle determined in situ by polarised fluorescence. Biophys Rev. 2024;16(6):753–72. 10.1007/s12551-024-01245-y. PMC11735716 39830118

[78] Lehman W, Pavadai E, Rynkiewicz MJ. C-terminal troponin-I residues trap tropomyosin in the muscle thin filament blocked-state. Biochem Biophys Res Commun. 2021;551:27–32. 10.1016/j.bbrc.2021.03.010. PMC8026701 33714756

[79] Akazawa H, Komuro I. Cardiac transcription factor Csx/Nkx2-5: its role in cardiac development and diseases. Pharmacol Ther. 2005;107(2):252–68. 10.1016/j.pharmthera.2005.03.005. 15925411

[80] Zammit PS. Function of the myogenic regulatory factors Myf5, MyoD, Myogenin and MRF4 in skeletal muscle, satellite cells and regenerative myogenesis. Semin Cell Dev Biol. 2017;72:19–32. 10.1016/j.semcdb.2017.11.011. 29127046

[81] Braun T, Gautel M. Transcriptional mechanisms regulating skeletal muscle differentiation, growth and homeostasis. Nat Rev Mol Cell Biol. 2011;12(6):349–61. 10.1038/nrm3118. 21602905

[82] Hernandez-Hernandez JM, Garcia-Gonzalez EG, Brun CE, Rudnicki MA. The myogenic regulatory factors, determinants of muscle development, cell identity and regeneration. Semin Cell Dev Biol. 2017;72:10–8. 10.1016/j.semcdb.2017.11.010. PMC5723221 29127045

[83] Lin J, Wu H, Tarr PT, Zhang CY, Wu Z, Boss O, et al. Transcriptional co-activator PGC-1 alpha drives the formation of slow-twitch muscle fibres. Nature. 2002;418(6899):797–801. 10.1038/nature00904. 12181572

[84] Petchey LK, Risebro CA, Vieira JM, Roberts T, Bryson JB, Greensmith L, et al. Loss of Prox1 in striated muscle causes slow to fast skeletal muscle fiber conversion and dilated cardiomyopathy. Proc Natl Acad Sci U S A. 2014;111(26):9515–20. 10.1073/pnas.1406191111. PMC4084422 24938781

[85] Vullhorst D, Buonanno A. Characterization of general transcription factor 3, a transcription factor involved in slow muscle-specific gene expression. J Biol Chem. 2003;278(10):8370–9. 10.1074/jbc.M209361200. 12475981

[86] Niro C, Demignon J, Vincent S, Liu Y, Giordani J, Sgarioto N, et al. Six1 and Six4 gene expression is necessary to activate the fast-type muscle gene program in the mouse primary myotome. Dev Biol. 2010;338(2):168–82. 10.1016/j.ydbio.2009.11.031. 19962975

[87] Ekmark M, Rana ZA, Stewart G, Hardie DG, Gundersen K. De-phosphorylation of MyoD is linking nerve-evoked activity to fast myosin heavy chain expression in rodent adult skeletal muscle. J Physiol. 2007;584(Pt 2):637–50. 10.1113/jphysiol.2007.141457. PMC2277165 17761773

[88] Hagiwara N, Ma B, Ly A. Slow and fast fiber isoform gene expression is systematically altered in skeletal muscle of the Sox6 mutant, p100H. Dev Dyn. 2005;234(2):301–11. 10.1002/dvdy.20535. 16124007

[89] Hagiwara N, Yeh M, Liu A. Sox6 is required for normal fiber type differentiation of fetal skeletal muscle in mice. Dev Dyn. 2007;236(8):2062–76. 10.1002/dvdy.21223. 17584907

[90] Zhou L, Liu Y, Lu L, Lu X, Dixon RA. Cardiac gene activation analysis in mammalian non-myoblasic cells by Nkx2-5, Tbx5, Gata4 and Myocd. PLoS ONE. 2012;7(10):e48028. 10.1371/journal.pone.0048028. PMC3483304 23144723

[91] Murphy AM, Thompson WR, Peng LF, Jones L 2nd. Regulation of the rat cardiac troponin I gene by the transcription factor GATA-4. Biochem J. 1997;322(Pt 2)(Pt 2):393–401. 10.1042/bj3220393. PMC1218204 9065755

[92] Cooper TA, Ordahl CP. A single troponin T gene regulated by different programs in cardiac and skeletal muscle development. Science. 1984;226(4677):979–82. 10.1126/science.6095446. 6095446

[93] Jin JP. Alternative RNA splicing-generated cardiac troponin T isoform switching: a non-heart-restricted genetic programming synchronized in developing cardiac and skeletal muscles. Biochem Biophys Res Commun. 1996;225(3):883–9. 10.1006/bbrc.1996.1267. 8780706

[94] Wang Q, Reiter RS, Huang QQ, Jin JP, Lin JJ. Comparative studies on the expression patterns of three troponin T genes during mouse development. Anat Rec. 2001;263(1):72–84. 10.1002/ar.1078. 11331973

[95] Toyota N, Shimada Y. Isoform variants of troponin in skeletal and cardiac muscle cells cultured with and without nerves. Cell. 1983;33(1):297–304. 10.1016/0092-8674(83)90358-6. 6380757

[96] Briggs MM, Schachat F. Origin of fetal troponin T: developmentally regulated splicing of a new exon in the fast troponin T gene. Dev Biol. 1993;158(2):503–9. 10.1006/dbio.1993.1208. 8344466

[97] Bhavsar PK, Dhoot GK, Cumming DV, Butler-Browne GS, Yacoub MH, Barton PJ. Developmental expression of troponin I isoforms in fetal human heart. FEBS Lett. 1991;292(1–2):5–8. 10.1016/0014-5793(91)80820-s. 1959627

[98] Sasse S, Brand NJ, Kyprianou P, Dhoot GK, Wade R, Arai M, et al. Troponin I gene expression during human cardiac development and in end-stage heart failure. Circ Res. 1993;72(5):932–8. 10.1161/01.res.72.5.932. 8477526

[99] Hastings KE, Emerson CP Jr. cDNA clone analysis of six co-regulated mRNAs encoding skeletal muscle contractile proteins. Proc Natl Acad Sci U S A. 1982;79(5):1553–7. 10.1073/pnas.79.5.1553. PMC346013 6951196

[100] Bodor GS, Porterfield D, Voss EM, Smith S, Apple FS. Cardiac troponin-I is not expressed in fetal and healthy or diseased adult human skeletal muscle tissue. Clin Chem. 1995;41(12 Pt 1):1710–5. 7497610

[101] Katrukha IA. Human cardiac troponin complex. Structure and functions. Biochemistry (Mosc). 2013;78(13):1447–65. 10.1134/S0006297913130063. 24490734

[102] Toyota N. Expression of troponin C genes during development in the chicken. Int J Dev Biol. 1993;37(4):531–7. 8179997

[103] Toyota N, Shimada Y. Differentiation of troponin in cardiac and skeletal muscles in chicken embryos as studied by immunofluorescence microscopy. J Cell Biol. 1981;91(2 Pt 1):497–504. 10.1083/jcb.91.2.497. PMC2111985 6171575

[104] Matsuda R, Obinata T, Shimada Y. Types of troponin components during development of chicken skeletal muscle. Dev Biol. 1981;82(1):11–9. 10.1016/0012-1606(81)90424-3. 7227630

[105] Gahlmann R, Wade R, Gunning P, Kedes L. Differential expression of slow and fast skeletal muscle troponin C. Slow skeletal muscle troponin C is expressed in human fibroblasts. J Mol Biol. 1988;201(2):379–91. 10.1016/0022-2836(88)90145-3. 3166492

[106] Ahmed RE, Tokuyama T, Anzai T, Chanthra N, Uosaki H. Sarcomere maturation: function acquisition, molecular mechanism, and interplay with other organelles. Philos Trans R Soc Lond B Biol Sci. 1864;2022(377):20210325. 10.1098/rstb.2021.0325. PMC9527934 36189811

[107] Siedner S, Kruger M, Schroeter M, Metzler D, Roell W, Fleischmann BK, et al. Developmental changes in contractility and sarcomeric proteins from the early embryonic to the adult stage in the mouse heart. J Physiol. 2003;548(Pt 2):493–505. 10.1113/jphysiol.2002.036509. PMC2342849 12640016

[108] Morimoto S, Goto T. Role of troponin I isoform switching in determining the pH sensitivity of Ca(2+) regulation in developing rabbit cardiac muscle. Biochem Biophys Res Commun. 2000;267(3):912–7. 10.1006/bbrc.1999.2068. 10673390

[109] Arteaga GM, Wolska BM, Martin AF, Kecskes SA, Solaro RJ. The effect of EMD 57033 is decreased in myofilaments from transgenic mouse hearts over-expressing slow skeletal troponin I (ssTnI) • 81. Pediatr Res. 1998;43(4):17. 10.1203/00006450-199804001-00102.

[110] Schiaffino S, Gorza L, Ausoni S. Troponin isoform switching in the developing heart and its functional consequences. Trends Cardiovasc Med. 1993;3(1):12–7. 10.1016/1050-1738(93)90022-X. 21244965

[111] Salameh S, Ogueri V, Posnack NG. Adapting to a new environment: postnatal maturation of the human cardiomyocyte. J Physiol. 2023;601(13):2593–619. 10.1113/JP283792. PMC10775138 37031380

[112] Fentzke RC, Buck SH, Patel JR, Lin H, Wolska BM, Stojanovic MO, et al. Impaired cardiomyocyte relaxation and diastolic function in transgenic mice expressing slow skeletal troponin I in the heart. J Physiol. 1999;517(Pt 1)(Pt 1):143–57. 10.1111/j.1469-7793.1999.0143z.x. PMC2269324 10226156

[113] Adamcova M, Pelouch V. Isoforms of troponin in normal and diseased myocardium. Physiol Res. 1999;48(4):235–47. 10638675

[114] Yin Z, Ren J, Guo W. Sarcomeric protein isoform transitions in cardiac muscle: a journey to heart failure. Biochim Biophys Acta. 2015;1852(1):47–52. 10.1016/j.bbadis.2014.11.003. PMC4268308 25446994

[115] Solaro RJ, Kobayashi T. Protein phosphorylation and signal transduction in cardiac thin filaments. J Biol Chem. 2011;286(12):9935–40. 10.1074/jbc.R110.197731. PMC3060547 21257760

[116] Zhang P, Kirk JA, Ji W, dos Remedios CG, Kass DA, Van Eyk JE, et al. Multiple reaction monitoring to identify site-specific troponin I phosphorylated residues in the failing human heart. Circulation. 2012;126(15):1828–37. 10.1161/CIRCULATIONAHA.112.096388. PMC3733556 22972900

[117] Li MX, Wang X, Lindhout DA, Buscemi N, Van Eyk JE, Sykes BD. Phosphorylation and mutation of human cardiac troponin I deferentially destabilize the interaction of the functional regions of troponin I with troponin C. Biochemistry. 2003;42(49):14460–8. 10.1021/bi035408y. 14661957

[118] Bilchick KC, Duncan JG, Ravi R, Takimoto E, Champion HC, Gao WD, et al. Heart failure-associated alterations in troponin I phosphorylation impair ventricular relaxation-afterload and force-frequency responses and systolic function. Am J Physiol Heart Circ Physiol. 2007;292(1):H318–25. 10.1152/ajpheart.00283.2006. 16936010

[119] Ravichandran VS, Patel HJ, Pagani FD, Westfall MV. Cardiac contractile dysfunction and protein kinase C-mediated myofilament phosphorylation in disease and aging. J Gen Physiol. 2019;151(9):1070–80. 10.1085/jgp.201912353. PMC6719401 31366607

[120] Steinberg SF. Oxidative stress and sarcomeric proteins. Circ Res. 2013;112(2):393–405. 10.1161/CIRCRESAHA.111.300496. PMC3595003 23329794

[121] Borbely A, Toth A, Edes I, Virag L, Papp JG, Varro A, et al. Peroxynitrite-induced alpha-actinin nitration and contractile alterations in isolated human myocardial cells. Cardiovasc Res. 2005;67(2):225–33. 10.1016/j.cardiores.2005.03.025. 16005301

[122] Maekawa A, Lee JK, Nagaya T, Kamiya K, Yasui K, Horiba M, et al. Overexpression of calpastatin by gene transfer prevents troponin I degradation and ameliorates contractile dysfunction in rat hearts subjected to ischemia/reperfusion. J Mol Cell Cardiol. 2003;35(10):1277–84. 10.1016/s0022-2828(03)00238-4. 14519437

[123] McDonough JL, Arrell DK, Van Eyk JE. Troponin I degradation and covalent complex formation accompanies myocardial ischemia/reperfusion injury. Circ Res. 1999;84(1):9–20. 10.1161/01.res.84.1.9. 9915770

[124] Palmer BS, Klawitter PF, Reiser PJ, Angelos MG. Degradation of rat cardiac troponin I during ischemia independent of reperfusion. Am J Physiol Heart Circ Physiol. 2004;287(3):H1269–75. 10.1152/ajpheart.00149.2004. 15142843

[125] Mair J. The pathophysiology of cardiac troponin release and the various circulating cardiac troponin forms-potential clinical implications. J Clin Med. 2025;14(12). 10.3390/jcm14124241. PMC12194301 40565986

[126] Rehsia NS, Dhalla NS. Mechanisms of the beneficial effects of beta-adrenoceptor antagonists in congestive heart failure. Exp Clin Cardiol. 2010;15(4):e86-95. PMC3016066 21264074

[127] Liu Y, Chen J, Fontes SK, Bautista EN, Cheng Z. Physiological and pathological roles of protein kinase A in the heart. Cardiovasc Res. 2022;118(2):386–98. 10.1093/cvr/cvab008. PMC8803072 33483740

[128] Akhtar MS, Hassan MQ, Siddiqui A, Alavudeen SS, Afzal O, Altamimi ASA, et al. Levosimendan: mechanistic insight and its diverse future aspects in cardiac care. Acta Cardiol. 2023;78(2):170–87. 10.1080/00015385.2022.2115761. 36222590

[129] Kasikcioglu HA, Cam N. A review of levosimendan in the treatment of heart failure. Vasc Health Risk Manag. 2006;2(4):389–400. 10.2147/vhrm.2006.2.4.389. PMC1994023 17323593

[130] Papp Z, Csapo K, Pollesello P, Haikala H, Edes I. Pharmacological mechanisms contributing to the clinical efficacy of levosimendan. Cardiovasc Drug Rev. 2005;23(1):71–98. 10.1111/j.1527-3466.2005.tb00158.x. 15867949

[131] Teerlink JR, Diaz R, Felker GM, McMurray JJV, Metra M, Solomon SD, et al. Cardiac myosin activation with omecamtiv mecarbil in systolic heart failure. N Engl J Med. 2021;384(2):105–16. 10.1056/NEJMoa2025797. 33185990

[132] Kallash M, Frishman WH, Aronow WS. Omecamtiv mecarbil, a cardiac myosin activator with potential efficacy in heart failure. Arch Med Sci Atheroscler Dis. 2025;10:e43–7. 10.5114/amsad/205547. PMC12223975 40620740

[133] Zhang T, Birbrair A, Wang ZM, Taylor J, Messi ML, Delbono O. Troponin T nuclear localization and its role in aging skeletal muscle. Age (Dordr). 2013;35(2):353–70. 10.1007/s11357-011-9368-4. PMC3592954 22189912

[134] Asumda FZ, Chase PB. Nuclear cardiac troponin and tropomyosin are expressed early in cardiac differentiation of rat mesenchymal stem cells. Differentiation. 2012;83(3):106–15. 10.1016/j.diff.2011.10.002. 22364878

[135] Bergmann O, Zdunek S, Alkass K, Druid H, Bernard S, Frisen J. Identification of cardiomyocyte nuclei and assessment of ploidy for the analysis of cell turnover. Exp Cell Res. 2011;317(2):188–94. 10.1016/j.yexcr.2010.08.017. 20828558

[136] Bergmann O, Bhardwaj RD, Bernard S, Zdunek S, Barnabe-Heider F, Walsh S, et al. Evidence for cardiomyocyte renewal in humans. Science. 2009;324(5923):98–102. 10.1126/science.1164680. PMC2991140 19342590

[137] Liu RM, Huang S, Hu D, Liu L, Sun HC, Tian J, et al. Decreased intranuclear cardiac troponin I impairs cardiac autophagy through FOS/ATG5 in ageing hearts. J Cell Mol Med. 2024;28(9):e18357. 10.1111/jcmm.18357. PMC11057418 38683127

[138] Chen C, Liu JB, Bian ZP, Xu JD, Wu HF, Gu CR, et al. Cardiac troponin I is abnormally expressed in non-small cell lung cancer tissues and human cancer cells. Int J Clin Exp Pathol. 2014;7(4):1314–24. PMC4014212 24817928

[139] Fritschle K, Mielke M, Seelbach OJ, Muhlthaler U, Zivanic M, Bozoglu T, et al. The V5-epitope tag for cell engineering and its use in immunohistochemistry and quantitative flow cytometry. Biology (Basel). 2025;14(7). 10.3390/biology14070890. PMC12292070 40723447

[140] Kim S, Kim J, Jung Y, Jun Y, Jung Y, Lee HY, et al. Characterization of TNNC1 as a novel tumor suppressor of lung adenocarcinoma. Mol Cells. 2020;43(7):619–31. 10.14348/molcells.2020.0075. PMC7398794 32638704

[141] Simon DN, Wilson KL. The nucleoskeleton as a genome-associated dynamic “network of networks.” Nat Rev Mol Cell Biol. 2011;12(11):695–708. 10.1038/nrm3207. 21971041

[142] Kelpsch DJ, Tootle TL. Nuclear actin: from discovery to function. Anat Rec (Hoboken). 2018;301(12):1999–2013. 10.1002/ar.23959. PMC6289869 30312531

[143] Shahid-Fuente IW, Toseland CP. Myosin in chromosome organisation and gene expression. Biochem Soc Trans. 2023;51(3):1023–34. 10.1042/BST20220939. PMC10317160 37171068

[144] King L, Jhou CR. Nuclear titin interacts with histones. Chang Gung Med J. 2010;33(2):201–10. 20438674

[145] Sobo JM, Alagna NS, Sun SX, Wilson KL, Reddy KL. Lamins: the backbone of the nucleocytoskeleton interface. Curr Opin Cell Biol. 2024;86:102313. 10.1016/j.ceb.2023.102313. 38262116

[146] Ye J, Zhao J, Hoffmann-Rohrer U, Grummt I. Nuclear myosin I acts in concert with polymeric actin to drive RNA polymerase I transcription. Genes Dev. 2008;22(3):322–30. 10.1101/gad.455908. PMC2216692 18230700

[147] Adam SA. The nucleoskeleton. Cold Spring Harb Perspect Biol. 2017;9(2). 10.1101/cshperspect.a023556. PMC5287075 28148597

[148] Miyamoto K, Harata M. Nucleoskeleton proteins for nuclear dynamics. J Biochem. 2021;169(3):237–41. 10.1093/jb/mvab006. 33479767

[149] Sidorenko E, Vartiainen MK. Nucleoskeletal regulation of transcription: Actin on MRTF. Exp Biol Med (Maywood). 2019;244(15):1372–81. 10.1177/1535370219854669. PMC6880152 31142145

[150] Gawor M, Lehka L, Lambert D, Toseland CP. Actin from within - how nuclear myosins and actin regulate nuclear architecture and mechanics. J Cell Sci. 2025;138(3). 10.1242/jcs.263550. PMC11883275 39927755

[151] Osmanagic-Myers S, Dechat T, Foisner R. Lamins at the crossroads of mechanosignaling. Genes Dev. 2015;29(3):225–37. 10.1101/gad.255968.114. PMC4318140 25644599

[152] Dechat T, Gesson K, Foisner R. Lamina-independent lamins in the nuclear interior serve important functions. Cold Spring Harb Symp Quant Biol. 2010;75:533–43. 10.1101/sqb.2010.75.018. 21209392

[153] Kapoor P, Shen X. Mechanisms of nuclear actin in chromatin-remodeling complexes. Trends Cell Biol. 2014;24(4):238–46. 10.1016/j.tcb.2013.10.007. PMC3995414 24246764

[154] Dechat T, Korbei B, Vaughan OA, Vlcek S, Hutchison CJ, Foisner R. Lamina-associated polypeptide 2alpha binds intranuclear A-type lamins. J Cell Sci. 2000;113(Pt 19):3473–84. 10.1242/jcs.113.19.3473. 10984438

[155] Brachner A, Foisner R. Lamina-associated polypeptide (LAP)2alpha and other LEM proteins in cancer biology. Adv Exp Med Biol. 2014;773:143–63. 10.1007/978-1-4899-8032-8_7. PMC4333762 24563347

[156] Zhang T, Taylor J, Jiang Y, Pereyra AS, Messi ML, Wang ZM, et al. Troponin T3 regulates nuclear localization of the calcium channel Cavbeta1a subunit in skeletal muscle. Exp Cell Res. 2015;336(2):276–86. 10.1016/j.yexcr.2015.05.005. PMC4522384 25981458

[157] Zhang T, Pereyra AS, Wang ZM, Birbrair A, Reisz JA, Files DC, et al. Calpain inhibition rescues troponin T3 fragmentation, increases Cav1.1, and enhances skeletal muscle force in aging sedentary mice. Aging Cell. 2016;15(3):488–98. 10.1111/acel.12453. PMC4854922 26892246

[158] Lu Q, Pan B, Bai H, Zhao W, Liu L, Li G, et al. Intranuclear cardiac troponin I plays a functional role in regulating Atp2a2 expression in cardiomyocytes. Genes Dis. 2022;9(6):1689–700. 10.1016/j.gendis.2021.04.007. PMC9485201 36157491

[159] Anderson PA, Malouf NN, Oakeley AE, Pagani ED, Allen PD. Troponin T isoform expression in the normal and failing human left ventricle: a correlation with myofibrillar ATPase activity. Basic Res Cardiol. 1992;87(Suppl 1):117–27. 10.1007/978-3-642-72474-9_10. 1386729

[160] Barton PJ, Felkin LE, Koban MU, Cullen ME, Brand NJ, Dhoot GK. The slow skeletal muscle troponin T gene is expressed in developing and diseased human heart. Mol Cell Biochem. 2004;263(1):91–7. 10.1023/B:MCBI.0000041851.53074.72. 27520668

[161] Bodor GS, Survant L, Voss EM, Smith S, Porterfield D, Apple FS. Cardiac troponin T composition in normal and regenerating human skeletal muscle. Clin Chem. 1997;43(3):476–84. 9068591

[162] Ricchiuti V, Voss EM, Ney A, Odland M, Anderson PA, Apple FS. Cardiac troponin T isoforms expressed in renal diseased skeletal muscle will not cause false-positive results by the second generation cardiac troponin T assay by Boehringer Mannheim. Clin Chem. 1998;44(9):1919–24. 9732977

[163] Ricchiuti V, Apple FS. RNA expression of cardiac troponin T isoforms in diseased human skeletal muscle. Clin Chem. 1999;45(12):2129–35. 10585344

[164] Huang QQ, Feng HZ, Liu J, Du J, Stull LB, Moravec CS, et al. Co-expression of skeletal and cardiac troponin T decreases mouse cardiac function. Am J Physiol Cell Physiol. 2008;294(1):C213–22. 10.1152/ajpcell.00146.2007. 17959729

[165] Huang QQ, Brozovich FV, Jin JP. Fast skeletal muscle troponin T increases the cooperativity of transgenic mouse cardiac muscle contraction. J Physiol. 1999;520 Pt 1(Pt 1):231–42. 10.1111/j.1469-7793.1999.00231.x. PMC2269565 10517814

[166] Pinto JR, Gomes AV, Jones MA, Liang J, Nguyen S, Miller T, et al. The functional properties of human slow skeletal troponin T isoforms in cardiac muscle regulation. J Biol Chem. 2012;287(44):37362–70. 10.1074/jbc.M112.364927. PMC3481333 22977240

[167] Zhang C, Zhang J, Guo K. Paeonol upregulates expression of tumor suppressors TNNC1 and SCARA5, exerting anti-tumor activity in non-small cell lung cancer cells. Naunyn Schmiedebergs Arch Pharmacol. 2024;397(7):5241–51. 10.1007/s00210-024-02963-6. 38265681

[168] Ge X, Du G, Zhou Q, Yan B, Yue G. TNNT1 accelerates migration, invasion and EMT progression in lung cancer cells. Thorac Cancer. 2024;15(23):1749–56. 10.1111/1759-7714.15400. PMC11320084 38973201

[169] Kapushesky M, Emam I, Holloway E, Kurnosov P, Zorin A, Malone J, et al. Gene expression atlas at the European bioinformatics institute. Nucleic Acids Res. 2010;38(Database issue):D690–8. 10.1093/nar/gkp936. PMC2808905 19906730

[170] Stewart M. Molecular mechanism of the nuclear protein import cycle. Nat Rev Mol Cell Biol. 2007;8(3):195–208. 10.1038/nrm2114. 17287812

[171] Wing CE, Fung HYJ, Chook YM. Karyopherin-mediated nucleocytoplasmic transport. Nat Rev Mol Cell Biol. 2022;23(5):307–28. 10.1038/s41580-021-00446-7. PMC10101760 35058649

[172] Paci G, Caria J, Lemke EA. Cargo transport through the nuclear pore complex at a glance. J Cell Sci. 2021;134(2). 10.1242/jcs.247874. 33495357

[173] Mor A, White MA, Fontoura BM. Nuclear trafficking in health and disease. Curr Opin Cell Biol. 2014;28:28–35. 10.1016/j.ceb.2014.01.007. PMC4061247 24530809

[174] Lange A, Mills RE, Lange CJ, Stewart M, Devine SE, Corbett AH. Classical nuclear localization signals: definition, function, and interaction with importin alpha. J Biol Chem. 2007;282(8):5101–5. 10.1074/jbc.R600026200. PMC4502416 17170104

[175] Lu J, Wu T, Zhang B, Liu S, Song W, Qiao J, et al. Types of nuclear localization signals and mechanisms of protein import into the nucleus. Cell Commun Signal. 2021;19(1):60. 10.1186/s12964-021-00741-y. PMC8140498 34022911

[176] Paci G, Zheng T, Caria J, Zilman A, Lemke EA. Molecular determinants of large cargo transport into the nucleus. Elife. 2020;9. 10.7554/eLife.55963. PMC7375812 32692309

[177] Nakai K, Horton P. PSORT: a program for detecting sorting signals in proteins and predicting their subcellular localization. Trends Biochem Sci. 1999;24(1):34–6. 10.1016/s0968-0004(98)01336-x. 10087920

[178] Zhang T, Birbrair A, Delbono O. Nonmyofilament-associated troponin T3 nuclear and nucleolar localization sequence and leucine zipper domain mediate muscle cell apoptosis. Cytoskeleton (Hoboken). 2013;70(3):134–47. 10.1002/cm.21095. PMC3667714 23378072

[179] Alber T. Structure of the leucine zipper. Curr Opin Genet Dev. 1992;2(2):205–10. 10.1016/s0959-437x(05)80275-8. 1638114

[180] Kumari A, Mussarrat A, Chakraborty M, Bhattacharjee A, Rajan RK. Leucine zipper proteins as emerging therapeutic regulators: structural dynamics, disease associations, and novel targeting strategies. Cell Cycle. 2025;24(21–24):552–65. 10.1080/15384101.2025.2585073. PMC12918286 41195551

[181] Laskowski RA, Jablonska J, Pravda L, Varekova RS, Thornton JM. PDBsum: structural summaries of PDB entries. Protein Sci. 2018;27(1):129–34. 10.1002/pro.3289. PMC5734310 28875543

[182] Hirst JD, Vieth M, Skolnick J, Brooks CL 3rd. Predicting leucine zipper structures from sequence. Protein Eng. 1996;9(8):657–62. 10.1093/protein/9.8.657. 8875642

[183] Bornberg-Bauer E, Rivals E, Vingron M. Computational approaches to identify leucine zippers. Nucleic Acids Res. 1998;26(11):2740–6. 10.1093/nar/26.11.2740. PMC147599 9592163

[184] Hulme JT, Scheuer T, Catterall WA. Regulation of cardiac ion channels by signaling complexes: role of modified leucine zipper motifs. J Mol Cell Cardiol. 2004;37(3):625–31. 10.1016/j.yjmcc.2004.04.014. 15350835

[185] Neduva V, Russell RB. Linear motifs: evolutionary interaction switches. FEBS Lett. 2005;579(15):3342–5. 10.1016/j.febslet.2005.04.005. 15943979

[186] Miller M. The importance of being flexible: the case of basic region leucine zipper transcriptional regulators. Curr Protein Pept Sci. 2009;10(3):244–69. 10.2174/138920309788452164. PMC2800128 19519454

[187] Madeira F, Madhusoodanan N, Lee J, Eusebi A, Niewielska A, Tivey ARN, et al. The EMBL-EBI Job Dispatcher sequence analysis tools framework in 2024. Nucleic Acids Res. 2024;52(W1):W521–5. 10.1093/nar/gkae241. PMC11223882 38597606

[188] Nunez Lopez YO, Messi ML, Pratley RE, Zhang T, Delbono O. Troponin T3 associates with DNA consensus sequence that overlaps with p53 binding motifs. Exp Gerontol. 2018;108:35–40. 10.1016/j.exger.2018.03.012. PMC5994179 29596868

[189] Sahota VK, Grau BF, Mansilla A, Ferrus A. Troponin I and tropomyosin regulate chromosomal stability and cell polarity. J Cell Sci. 2009;122(Pt 15):2623–31. 10.1242/jcs.050880. 19567471

[190] Kharitonov AV, Shubina MY, Nosov GA, Mamontova AV, Arifulin EA, Lisitsyna OM, et al. Switching of cardiac troponin I between nuclear and cytoplasmic localization during muscle differentiation. Biochim Biophys Acta Mol Cell Res. 2020;1867(2):118601. 10.1016/j.bbamcr.2019.118601. 31733262

[191] Chook YM, Suel KE. Nuclear import by karyopherin-betas: recognition and inhibition. Biochim Biophys Acta. 2011;1813(9):1593–606. 10.1016/j.bbamcr.2010.10.014. PMC3135726 21029754

[192] MacNeil DE, Lambert-Lanteigne P, Qin J, McManus FP, Bonneil E, Thibault P, et al. SUMOylation- and GAR1-dependent regulation of dyskerin nuclear and subnuclear localization. Mol Cell Biol. 2021;41(4). 10.1128/MCB.00464-20. PMC8088130 33526451

[193] Moreno-Onate M, Herrero-Ruiz AM, Garcia-Dominguez M, Cortes-Ledesma F, Ruiz JF. RanBP2-mediated SUMOylation promotes human DNA polymerase lambda nuclear localization and DNA Repair. J Mol Biol. 2020;432(13):3965–79. 10.1016/j.jmb.2020.03.020. 32224012

[194] Stubbe M, Mai J, Paulus C, Stubbe HC, Berscheminski J, Karimi M, et al. Viral DNA binding protein SUMOylation promotes PML nuclear body localization next to viral replication centers. MBio. 2020;11(2). 10.1128/mBio.00049-20. PMC7078464 32184235

[195] Fertig B, Ling J, Nollet EE, Dobi S, Busiau T, Ishikawa K, et al. SUMOylation does not affect cardiac troponin I stability but alters indirectly the development of force in response to Ca(2). FEBS J. 2022;289(20):6267–85. 10.1111/febs.16537. PMC9588612 35633070

[196] Funk WD, Pak DT, Karas RH, Wright WE, Shay JW. A transcriptionally active DNA-binding site for human p53 protein complexes. Mol Cell Biol. 1992;12(6):2866–71. 10.1128/mcb.12.6.2866-2871.1992. PMC364481 1588974

[197] Riley T, Sontag E, Chen P, Levine A. Transcriptional control of human p53-regulated genes. Nat Rev Mol Cell Biol. 2008;9(5):402–12. 10.1038/nrm2395. 18431400

[198] Aye Y, Li M, Long MJ, Weiss RS. Ribonucleotide reductase and cancer: biological mechanisms and targeted therapies. Oncogene. 2015;34(16):2011–21. 10.1038/onc.2014.155. 24909171

[199] Cho EC, Kuo ML, Cheng JH, Cheng YC, Hsieh YC, Liu YR, et al. RRM2B-mediated regulation of mitochondrial activity and inflammation under oxidative stress. Mediators Inflamm. 2015;2015:287345. 10.1155/2015/287345. PMC4451759 26089597

[200] Zilfou JT, Lowe SW. Tumor suppressive functions of p53. Cold Spring Harb Perspect Biol. 2009;1(5):a001883. 10.1101/cshperspect.a001883. PMC2773645 20066118

[201] Yant SR, Zhu W, Millinoff D, Slightom JL, Goodman M, Gumucio DL. High affinity YY1 binding motifs: identification of two core types (ACAT and CCAT) and distribution of potential binding sites within the human beta globin cluster. Nucleic Acids Res. 1995;23(21):4353–62. 10.1093/nar/23.21.4353. PMC307390 7501456

[202] Kim J, Kim J. YY1’s longer DNA-binding motifs. Genomics. 2009;93(2):152–8. 10.1016/j.ygeno.2008.09.013. PMC2668202 18950698

[203] Golebiowski FM, Gorecki A, Bonarek P, Rapala-Kozik M, Kozik A, Dziedzicka-Wasylewska M. An investigation of the affinities, specificity and kinetics involved in the interaction between the Yin Yang 1 transcription factor and DNA. FEBS J. 2012;279(17):3147–58. 10.1111/j.1742-4658.2012.08693.x. 22776217

[204] Donohoe ME, Zhang X, McGinnis L, Biggers J, Li E, Shi Y. Targeted disruption of mouse Yin Yang 1 transcription factor results in peri-implantation lethality. Mol Cell Biol. 1999;19(10):7237–44. 10.1128/MCB.19.10.7237. PMC84716 10490658

[205] Martinez-Ruiz GU, Morales-Sanchez A, Pacheco-Hernandez AF. Roles played by YY1 in embryonic, adult and cancer stem cells. Stem Cell Rev Rep. 2021;17(5):1590–606. 10.1007/s12015-021-10151-9. PMC8553684 33728560

[206] Wu S, Shi Y, Mulligan P, Gay F, Landry J, Liu H, et al. A YY1-INO80 complex regulates genomic stability through homologous recombination-based repair. Nat Struct Mol Biol. 2007;14(12):1165–72. 10.1038/nsmb1332. PMC2754171 18026119

[207] Agarwal N, Theodorescu D. The role of transcription factor YY1 in the biology of cancer. Crit Rev Oncog. 2017;22(1–2):13–21. 10.1615/CritRevOncog.2017021071. PMC8788901 29604933

[208] Verheul TCJ, van Hijfte L, Perenthaler E, Barakat TS. The why of YY1: mechanisms of transcriptional regulation by Yin Yang 1. Front Cell Dev Biol. 2020;8:592164. 10.3389/fcell.2020.592164. PMC7554316 33102493

[209] Shrivastava A, Saleque S, Kalpana GV, Artandi S, Goff SP, Calame K. Inhibition of transcriptional regulator Yin-Yang-1 by association with c-Myc. Science. 1993;262(5141):1889–92. 10.1126/science.8266081. 8266081

[210] Yao YL, Yang WM, Seto E. Regulation of transcription factor YY1 by acetylation and deacetylation. Mol Cell Biol. 2001;21(17):5979–91. 10.1128/MCB.21.17.5979-5991.2001. PMC87316 11486036

[211] Wang CY, Liang YJ, Lin YS, Shih HM, Jou YS, Yu WC. YY1AP, a novel co-activator of YY1. J Biol Chem. 2004;279(17):17750–5. 10.1074/jbc.M310532200. 14744866

[212] Gordon S, Akopyan G, Garban H, Bonavida B. Transcription factor YY1: structure, function, and therapeutic implications in cancer biology. Oncogene. 2006;25(8):1125–42. 10.1038/sj.onc.1209080. 16314846

[213] Lewis BA, Tullis G, Seto E, Horikoshi N, Weinmann R, Shenk T. Adenovirus E1A proteins interact with the cellular YY1 transcription factor. J Virol. 1995;69(3):1628–36. 10.1128/JVI.69.3.1628-1636.1995. PMC188760 7853498

[214] Huang NE, Lin CH, Lin YS, Yu WC. Modulation of YY1 activity by SAP30. Biochem Biophys Res Commun. 2003;306(1):267–75. 10.1016/s0006-291x(03)00966-5. 12788099

[215] Yang WM, Inouye C, Zeng Y, Bearss D, Seto E. Transcriptional repression by YY1 is mediated by interaction with a mammalian homolog of the yeast global regulator RPD3. Proc Natl Acad Sci U S A. 1996;93(23):12845–50. 10.1073/pnas.93.23.12845. PMC24008 8917507

[216] Xue X, Li Z, Zhao J, Zhao Z, Li Z, Li Y, et al. Advances in the relationship between AP-1 and tumorigenesis, development and therapy resistance. Discov Oncol. 2025;16(1):61. 10.1007/s12672-025-01783-1. PMC11747019 39831917

[217] Natesan S, Gilman MZ. DNA bending and orientation-dependent function of YY1 in the c-fos promoter. Genes Dev. 1993;7(12B):2497–509. 10.1101/gad.7.12b.2497. 8276234

[218] Zhou Q, Gedrich RW, Engel DA. Transcriptional repression of the c-fos gene by YY1 is mediated by a direct interaction with ATF/CREB. J Virol. 1995;69(7):4323–30. 10.1128/JVI.69.7.4323-4330.1995. PMC189172 7769693

[219] Natesan S, Gilman M. YY1 facilitates the association of serum response factor with the c-fos serum response element. Mol Cell Biol. 1995;15(11):5975–82. 10.1128/MCB.15.11.5975. PMC230849 7565750

[220] Changotra H, Kaur S, Yadav SS, Gupta GL, Parkash J, Duseja A. ATG5: a central autophagy regulator implicated in various human diseases. Cell Biochem Funct. 2022;40(7):650–67. 10.1002/cbf.3740. 36062813

[221] Li Y, Chen B, Chen J, Lou G, Chen S, Zhou D. Fast skeletal muscle troponin I is a co-activator of estrogen receptor-related receptor alpha. Biochem Biophys Res Commun. 2008;369(4):1034–40. 10.1016/j.bbrc.2008.02.147. 18331830

[222] Villena JA, Kralli A. ERRalpha: a metabolic function for the oldest orphan. Trends Endocrinol Metab. 2008;19(8):269–76. 10.1016/j.tem.2008.07.005. PMC2786240 18778951

[223] Lee HR, Kim TH, Choi KC. Functions and physiological roles of two types of estrogen receptors, ERalpha and ERbeta, identified by estrogen receptor knockout mouse. Lab Anim Res. 2012;28(2):71–6. 10.5625/lar.2012.28.2.71. PMC3389841 22787479

[224] Bramlett KS, Wu Y, Burris TP. Ligands specify coactivator nuclear receptor (NR) box affinity for estrogen receptor subtypes. Mol Endocrinol. 2001;15(6):909–22. 10.1210/mend.15.6.0649. 11376110

[225] Stein RA, Chang CY, Kazmin DA, Way J, Schroeder T, Wergin M, et al. Estrogen-related receptor alpha is critical for the growth of estrogen receptor-negative breast cancer. Cancer Res. 2008;68(21):8805–12. 10.1158/0008-5472.CAN-08-1594. PMC2633645 18974123

[226] Chaltel-Lima L, Dominguez F, Dominguez-Ramirez L, Cortes-Hernandez P. The role of the estrogen-related receptor alpha (ERRa) in hypoxia and its implications for cancer metabolism. Int J Mol Sci. 2023;24(9). 10.3390/ijms24097983. PMC10178695 37175690

[227] Zhao W, Liu L, Pan B, Xu Y, Zhu J, Nan C, et al. Epigenetic regulation of cardiac myofibril gene expression during heart development. Cardiovasc Toxicol. 2015;15(3):203–9. 10.1007/s12012-014-9278-7. 25296860

[228] Du J, Liu J, Feng HZ, Hossain MM, Gobara N, Zhang C, et al. Impaired relaxation is the main manifestation in transgenic mice expressing a restrictive cardiomyopathy mutation, R193H, in cardiac TnI. Am J Physiol Heart Circ Physiol. 2008;294(6):H2604–13. 10.1152/ajpheart.91506.2007. PMC2769498 18408133

[229] Davis J, Yasuda S, Palpant NJ, Martindale J, Stevenson T, Converso K, et al. Diastolic dysfunction and thin filament dysregulation resulting from excitation-contraction uncoupling in a mouse model of restrictive cardiomyopathy. J Mol Cell Cardiol. 2012;53(3):446–57. 10.1016/j.yjmcc.2012.05.018. PMC3443869 22683325

[230] Sherstnev I, Judina A, Luciani GB, Ghigo A, Hirsch E, Gorelik J. Role of PDE4 family in cardiomyocyte physiology and heart failure. Cells. 2025;14(6). 10.3390/cells14060460. PMC11941749 40136709

[231] Berkholz J, Orgeur M, Stricker S, Munz B. skNAC and Smyd1 in transcriptional control. Exp Cell Res. 2015;336(2):182–91. 10.1016/j.yexcr.2015.06.019. 26162853

[232] Spellmon N, Holcomb J, Trescott L, Sirinupong N, Yang Z. Structure and function of SET and MYND domain-containing proteins. Int J Mol Sci. 2015;16(1):1406–28. 10.3390/ijms16011406. PMC4307310 25580534

[233] Tracy C, Warren JS, Szulik M, Wang L, Garcia J, Makaju A, et al. The Smyd family of methyltransferases: role in cardiac and skeletal muscle physiology and pathology. Curr Opin Physiol. 2018;1:140–52. 10.1016/j.cophys.2017.10.001. PMC5807017 29435515

[234] Beca S, Helli PB, Simpson JA, Zhao D, Farman GP, Jones P, et al. Phosphodiesterase 4D regulates baseline sarcoplasmic reticulum Ca2+ release and cardiac contractility, independently of L-type Ca2+ current. Circ Res. 2011;109(9):1024–30. 10.1161/CIRCRESAHA.111.250464. PMC4138878 21903937

[235] Sommese RF, Nag S, Sutton S, Miller SM, Spudich JA, Ruppel KM. Effects of troponin T cardiomyopathy mutations on the calcium sensitivity of the regulated thin filament and the actomyosin cross-bridge kinetics of human beta-cardiac myosin. PLoS ONE. 2013;8(12):e83403. 10.1371/journal.pone.0083403. PMC3867432 24367593

[236] Wu H, Lee J, Vincent LG, Wang Q, Gu M, Lan F, et al. Epigenetic regulation of phosphodiesterases 2A and 3A underlies compromised beta-adrenergic signaling in an iPSC model of dilated cardiomyopathy. Cell Stem Cell. 2015;17(1):89–100. 10.1016/j.stem.2015.04.020. PMC4546705 26095046

[237] Jones PA. Functions of DNA methylation: islands, start sites, gene bodies and beyond. Nat Rev Genet. 2012;13(7):484–92. 10.1038/nrg3230. 22641018

[238] Mangiavacchi A, Morelli G, Orlando V. Behind the scenes: how RNA orchestrates the epigenetic regulation of gene expression. Front Cell Dev Biol. 2023;11:1123975. 10.3389/fcell.2023.1123975. PMC9905133 36760365

[239] Monteith GR, Prevarskaya N, Roberts-Thomson SJ. The calcium-cancer signalling nexus. Nat Rev Cancer. 2017;17(6):367–80. 10.1038/nrc.2017.18. 28386091

[240] Cai J, Qiu J, Wang H, Sun J, Ji Y. Identification of potential biomarkers in ovarian carcinoma and an evaluation of their prognostic value. Ann Transl Med. 2021;9(18):1472. 10.21037/atm-21-4606. PMC8506714 34734024

[241] Vergnol A, Traore M, Pietri-Rouxel F, Falcone S. New insights in CaVbeta subunits: role in the regulation of gene expression and cellular homeostasis. Front Cell Dev Biol. 2022;10:880441. 10.3389/fcell.2022.880441. PMC9019481 35465309

[242] Rima M, Daghsni M, Fajloun Z, M’Rad R, Bruses JL, Ronjat M, et al. Protein partners of the calcium channel beta subunit highlight new cellular functions. Biochem J. 2016;473(13):1831–44. 10.1042/BCJ20160125. 27354560

[243] Menck K, Sivaloganathan S, Bleckmann A, Binder C. Microvesicles in cancer: small size, large potential. Int J Mol Sci. 2020;21(15). 10.3390/ijms21155373. PMC7432491 32731639

[244] Chitti SV, Gummadi S, Kang T, Shahi S, Marzan AL, Nedeva C, et al. Vesiclepedia 2024: an extracellular vesicles and extracellular particles repository. Nucleic Acids Res. 2024;52(D1):D1694–8. 10.1093/nar/gkad1007. PMC10767981 37953359

[245] Rahat MA, Kzhyshkowska J, Iragavarapu-Charyulu V. Editorial: targeting angiogenesis to treat autoimmune diseases and cancer. Front Immunol. 2020;11:1005. 10.3389/fimmu.2020.01005. PMC7253682 32528478

[246] Moses MA, Wiederschain D, Wu I, Fernandez CA, Ghazizadeh V, Lane WS, et al. Troponin I is present in human cartilage and inhibits angiogenesis. Proc Natl Acad Sci U S A. 1999;96(6):2645–50. 10.1073/pnas.96.6.2645. PMC15822 10077564

[247] Hanahan D, Weinberg RA. Hallmarks of cancer: the next generation. Cell. 2011;144(5):646–74. 10.1016/j.cell.2011.02.013. 21376230

[248] Davis J, Wen H, Edwards T, Metzger JM. Thin filament disinhibition by restrictive cardiomyopathy mutant R193H troponin I induces Ca2+-independent mechanical tone and acute myocyte remodeling. Circ Res. 2007;100(10):1494–502. 10.1161/01.RES.0000268412.34364.50. 17463320

[249] Luo J, Zhao W, Gan Y, Pan B, Liu L, Liu Z, et al. Cardiac troponin I R193H mutation is associated with mitochondrial damage in cardiomyocytes. DNA Cell Biol. 2021;40(2):184–91. 10.1089/dna.2020.5828. 33465007

[250] Orchard S, Ammari M, Aranda B, Breuza L, Briganti L, Broackes-Carter F, et al. The MIntAct project--IntAct as a common curation platform for 11 molecular interaction databases. Nucleic Acids Res. 2014;42(Database issue):D358-63. 10.1093/nar/gkt1115. PMC3965093 24234451

[251] Vilar S, Cozza G, Moro S. Medicinal chemistry and the molecular operating environment (MOE): application of QSAR and molecular docking to drug discovery. Curr Top Med Chem. 2008;8(18):1555–72. 10.2174/156802608786786624. 19075767

[252] Alam MS. Proximity Ligation Assay (PLA). Curr Protoc Immunol. 2018;123(1):e58. 10.1002/cpim.58. PMC6205916 30238640

[253] Young RM. Proximity ligation assay. Methods Mol Biol. 1956;2019:363–70. 10.1007/978-1-4939-9151-8_18. 30779045

[254] Wang P, Yang Y, Hong T, Zhu G. Proximity ligation assay: an ultrasensitive method for protein quantification and its applications in pathogen detection. Appl Microbiol Biotechnol. 2021;105(3):923–35. 10.1007/s00253-020-11049-1. 33427935

[255] Gao H, Wang K, Li H, Fan Y, Sun X, Wang X, et al. Recent advances in electrochemical proximity ligation assay. Talanta. 2023;254:124158. 10.1016/j.talanta.2022.124158. 36502611

[256] Yang Z, Marston SB, Gould IR. Modulation of structure and dynamics of cardiac troponin by phosphorylation and mutations revealed by molecular dynamics simulations. J Phys Chem B. 2023;127(41):8736–48. 10.1021/acs.jpcb.3c02337. PMC10591477 37791815

[257] Gaze DC, Collinson PO. Multiple molecular forms of circulating cardiac troponin: analytical and clinical significance. Ann Clin Biochem. 2008;45(Pt 4):349–55. 10.1258/acb.2007.007229. 18583618

[258] Al-Otaiby MA, Al-Amri HS, Al-Moghairi AM. The clinical significance of cardiac troponins in medical practice. J Saudi Heart Assoc. 2011;23(1):3–11. 10.1016/j.jsha.2010.10.001. PMC3727460 23960628

[259] Babuin L, Jaffe AS. Troponin: the biomarker of choice for the detection of cardiac injury. CMAJ. 2005;173(10):1191–202. 10.1503/cmaj/051291. PMC1277047 16275971

[260] Chen Z, Lu W, Garcia-Prieto C, Huang P. The Warburg effect and its cancer therapeutic implications. J Bioenerg Biomembr. 2007;39(3):267–74. 10.1007/s10863-007-9086-x. 17551814

[261] Zong WX, Rabinowitz JD, White E. Mitochondria and cancer. Mol Cell. 2016;61(5):667–76. 10.1016/j.molcel.2016.02.011. PMC4779192 26942671

[262] Tan YQ, Zhang X, Zhang S, Zhu T, Garg M, Lobie PE, et al. Mitochondria: the metabolic switch of cellular oncogenic transformation. Biochim Biophys Acta Rev Cancer. 2021;1876(1):188534. 10.1016/j.bbcan.2021.188534. 33794332

[263] Kenny TC, Birsoy K. Mitochondria and cancer. Cold Spring Harb Perspect Med. 2024;14(12). 10.1101/cshperspect.a041534. PMC11610758 38692736

[264] Esparza-Molto PB, Cuezva JM. The role of mitochondrial H(+)-ATP synthase in cancer. Front Oncol. 2018;8:53. 10.3389/fonc.2018.00053. PMC5845864 29564224

[265] Galber C, Acosta MJ, Minervini G, Giorgio V. The role of mitochondrial ATP synthase in cancer. Biol Chem. 2020;401(11):1199–214. 10.1515/hsz-2020-0157. 32769215

[266] Cuezva JM, Dominguez-Zorita S. The ATPase Inhibitory Factor 1 (IF1) contributes to the Warburg Effect and is regulated by its phosphorylation in S39 by a protein kinase A-like activity. Cancers (Basel). 2024;16(5). 10.3390/cancers16051014. PMC10931224 38473373

[267] Solaini G, Sgarbi G, Baracca A. The F1Fo-ATPase inhibitor, IF1, is a critical regulator of energy metabolism in cancer cells. Biochem Soc Trans. 2021;49(2):815–27. 10.1042/BST20200742. 33929490

[268] Gatto C, Grandi M, Solaini G, Baracca A, Giorgio V. The F1Fo-ATPase inhibitor protein IF1 in pathophysiology. Front Physiol. 2022;13:917203. 10.3389/fphys.2022.917203. PMC9389554 35991181

[269] Faccenda D, Campanella M. Molecular regulation of the mitochondrial F(1)F(o)-ATPsynthase: physiological and pathological significance of the inhibitory factor 1 (IF(1)). Int J Cell Biol. 2012;2012:367934. 10.1155/2012/367934. PMC3433140 22966230

[270] Gledhill JR, Montgomery MG, Leslie AG, Walker JE. How the regulatory protein, IF(1), inhibits F(1)-ATPase from bovine mitochondria. Proc Natl Acad Sci U S A. 2007;104(40):15671–6. 10.1073/pnas.0707326104. PMC1994141 17895376

[271] Jonckheere AI, Smeitink JA, Rodenburg RJ. Mitochondrial ATP synthase: architecture, function and pathology. J Inherit Metab Dis. 2012;35(2):211–25. 10.1007/s10545-011-9382-9. PMC3278611 21874297

[272] Patel BA, D’Amico TL, Blagg BSJ. Natural products and other inhibitors of F(1)F(O) ATP synthase. Eur J Med Chem. 2020;207:112779. 10.1016/j.ejmech.2020.112779. PMC7891226 32942072

[273] Fang C, Zhang X, Li C, Liu F, Liu H. Troponin C-1 activated by E2F1 accelerates gastric cancer progression via regulating TGF-beta/Smad signaling. Dig Dis Sci. 2022;67(9):4444–57. 10.1007/s10620-021-07287-6. 34797443

[274] Tsuruda T, Sato Y, Tomita M, Tanaka H, Hatakeyama K, Otsu M, et al. Aberrant expression of cardiac troponin-T in lung cancer tissues in association with pathological severity. Front Cardiovasc Med. 2022;9:833649. 10.3389/fcvm.2022.833649. PMC9037982 35479276

[275] Casas-Tinto S, Maraver A, Serrano M, Ferrus A. Troponin-I enhances and is required for oncogenic overgrowth. Oncotarget. 2016;7(33):52631–42. 10.18632/oncotarget.10616. PMC5288137 27437768

[276] Pitts S, Laiho M. Regulation of RNA polymerase I stability and function. Cancers (Basel). 2022;14(23). 10.3390/cancers14235776. PMC9737084 36497261

[277] Turner N, Grose R. Fibroblast growth factor signalling: from development to cancer. Nat Rev Cancer. 2010;10(2):116–29. 10.1038/nrc2780. 20094046

[278] Jing Q, Wang Y, Liu H, Deng X, Jiang L, Liu R, et al. FGFs: crucial factors that regulate tumour initiation and progression. Cell Prolif. 2016;49(4):438–47. 10.1111/cpr.12275. PMC6496028 27383016

[279] Akl MR, Nagpal P, Ayoub NM, Tai B, Prabhu SA, Capac CM, et al. Molecular and clinical significance of fibroblast growth factor 2 (FGF2 /bFGF) in malignancies of solid and hematological cancers for personalized therapies. Oncotarget. 2016;7(28):44735–62. 10.18632/oncotarget.8203. PMC5190132 27007053

[280] Ardizzone A, Bova V, Casili G, Repici A, Lanza M, Giuffrida R, et al. Role of basic fibroblast growth factor in cancer: biological activity, targeted therapies, and prognostic value. Cells. 2023;12(7). 10.3390/cells12071002. PMC10093572 37048074

[281] Circu ML, Aw TY. Reactive oxygen species, cellular redox systems, and apoptosis. Free Radic Biol Med. 2010;48(6):749–62. 10.1016/j.freeradbiomed.2009.12.022. PMC2823977 20045723

[282] Redza-Dutordoir M, Averill-Bates DA. Activation of apoptosis signalling pathways by reactive oxygen species. Biochim Biophys Acta. 2016;1863(12):2977–92. 10.1016/j.bbamcr.2016.09.012. 27646922

[283] Scholtes C, Giguere V. Transcriptional regulation of ROS homeostasis by the ERR subfamily of nuclear receptors. Antioxidants (Basel). 2021;10(3). 10.3390/antiox10030437. PMC7999130 33809291

[284] Xia H, Dufour CR, Giguere V. ERRalpha as a bridge between transcription and function: role in liver metabolism and disease. Front Endocrinol (Lausanne). 2019;10:206. 10.3389/fendo.2019.00206. PMC6459935 31024446

[285] Hong EJ, Levasseur MP, Dufour CR, Perry MC, Giguere V. Loss of estrogen-related receptor alpha promotes hepatocarcinogenesis development via metabolic and inflammatory disturbances. Proc Natl Acad Sci U S A. 2013;110(44):17975–80. 10.1073/pnas.1315319110. PMC3816417 24127579

[286] Cerutti C, Shi JR, Vanacker JM. Multifaceted transcriptional network of estrogen-related receptor alpha in health and disease. Int J Mol Sci. 2023;24(5). 10.3390/ijms24054265. PMC10002233 36901694

[287] Milacic M, Beavers D, Conley P, Gong C, Gillespie M, Griss J, et al. The reactome pathway knowledgebase 2024. Nucleic Acids Res. 2024;52(D1):D672–8. 10.1093/nar/gkad1025. PMC10767911 37941124

[288] Yang X, Wu K, Li S, Hu L, Han J, Zhu D, et al. MFAP5 and TNNC1: potential markers for predicting occult cervical lymphatic metastasis and prognosis in early stage tongue cancer. Oncotarget. 2017;8(2):2525–35. 10.18632/oncotarget.12446. PMC5356821 27713166

[289] Ma F, Wang Z, Qiang Y, Xu L, Ding P, Wang Y, et al. LukS-PV inhibits hepatocellular carcinoma cells migration via the TNNC1/PI3K/AKT Axis. Onco Targets Ther. 2020;13:10221–30. 10.2147/OTT.S278540. PMC7578518 33116603

[290] Yin JH, Elumalai P, Kim SY, Zhang SZ, Shin S, Lee M, et al. TNNC1 knockout reverses metastatic potential of ovarian cancer cells by inactivating epithelial-mesenchymal transition and suppressing F-actin polymerization. Biochem Biophys Res Commun. 2021;547:44–51. 10.1016/j.bbrc.2021.02.021. 33592378

[291] Leung CS, Yeung TL, Yip KP, Pradeep S, Balasubramanian L, Liu J, et al. Calcium-dependent FAK/CREB/TNNC1 signalling mediates the effect of stromal MFAP5 on ovarian cancer metastatic potential. Nat Commun. 2014;5:5092. 10.1038/ncomms6092. PMC4185407 25277212

[292] Tsai PJ, Lai YH, Manne RK, Tsai YS, Sarbassov D, Lin HK. Akt: a key transducer in cancer. J Biomed Sci. 2022;29(1):76. 10.1186/s12929-022-00860-9. PMC9526305 36180910

[293] Kao SH, Wang WL, Chen CY, Chang YL, Wu YY, Wang YT, et al. GSK3beta controls epithelial-mesenchymal transition and tumor metastasis by CHIP-mediated degradation of Slug. Oncogene. 2017;36(42):5916. 10.1038/onc.2017.302. 28869596

[294] Maharati A, Moghbeli M. PI3K/AKT signaling pathway as a critical regulator of epithelial-mesenchymal transition in colorectal tumor cells. Cell Commun Signal. 2023;21(1):201. 10.1186/s12964-023-01225-x. PMC10424373 37580737

[295] Choi SH, Kim JK, Chen CT, Wu C, Marco MR, Barriga FM, et al. KRAS mutants upregulate integrin beta4 to promote invasion and metastasis in colorectal cancer. Mol Cancer Res. 2022;20(8):1305–19. 10.1158/1541-7786.MCR-21-0994. PMC9357101 35394541

[296] Murrell M, Oakes PW, Lenz M, Gardel ML. Forcing cells into shape: the mechanics of actomyosin contractility. Nat Rev Mol Cell Biol. 2015;16(8):486–98. 10.1038/nrm4012. PMC7443980 26130009

[297] Davis MJ, Earley S, Li YS, Chien S. Vascular mechanotransduction. Physiol Rev. 2023;103(2):1247–421. 10.1152/physrev.00053.2021. PMC9942936 36603156

[298] Moran CM, Garriock RJ, Miller MK, Heimark RL, Gregorio CC, Krieg PA. Expression of the fast twitch troponin complex, fTnT, fTnI and fTnC, in vascular smooth muscle. Cell Motil Cytoskeleton. 2008;65(8):652–61. 10.1002/cm.20291. PMC2570210 18548613

[299] Kajioka S, Takahashi-Yanaga F, Shahab N, Onimaru M, Matsuda M, Takahashi R, et al. Endogenous cardiac troponin T modulates Ca(2+)-mediated smooth muscle contraction. Sci Rep. 2012;2:979. 10.1038/srep00979. PMC3522072 23248744

[300] Schaks M, Giannone G, Rottner K. Actin dynamics in cell migration. Essays Biochem. 2019;63(5):483–95. 10.1042/EBC20190015. PMC6823167 31551324

[301] SenGupta S, Parent CA, Bear JE. The principles of directed cell migration. Nat Rev Mol Cell Biol. 2021;22(8):529–47. 10.1038/s41580-021-00366-6. PMC8663916 33990789

[302] Aseervatham J. Cytoskeletal remodeling in cancer. Biology (Basel). 2020;9(11). 10.3390/biology9110385. PMC7695181 33171868

[303] Pratiwi L, Elisa E, Sutanto H. Probing the protrusions: lamellipodia and filopodia in cancer invasion and beyond. Mechanobiol Med. 2024;2(2):100064. 10.1016/j.mbm.2024.100064. PMC12082305 40395858

[304] Jin Y, Yang Y. Bioinformatics-based discovery of PYGM and TNNC2 as potential biomarkers of head and neck squamous cell carcinoma. Biosci Rep. 2019;39(7). 10.1042/BSR20191612. PMC6663994 31324732

[305] Dickinson A, Saraswat M, Makitie A, Silen R, Hagstrom J, Haglund C, et al. Label-free tissue proteomics can classify oral squamous cell carcinoma from healthy tissue in a stage-specific manner. Oral Oncol. 2018;86:206–15. 10.1016/j.oraloncology.2018.09.013. 30409303

[306] Zhang Q, Hao L, Wang F, Yu Q, Wu S, Han C. Troponin T1 in tumorigenesis and immune modulation: insights into multiple cancers and kidney renal clear cell carcinoma. J Cell Mol Med. 2024;28(11):e18410. 10.1111/jcmm.18410. PMC11163025 38853457

[307] Hao YH, Yu SY, Tu RS, Cai YQ. TNNT1, a prognostic indicator in colon adenocarcinoma, regulates cell behaviors and mediates EMT process. Biosci Biotechnol Biochem. 2020;84(1):111–7. 10.1080/09168451.2019.1664891. 31512553

[308] Jope RS, Bijur GN. Mood stabilizers, glycogen synthase kinase-3beta and cell survival. Mol Psychiatry. 2002;7(Suppl 1):S35-45. 10.1038/sj.mp.4001017. 11986994

[309] Li H, Huang K, Liu X, Liu J, Lu X, Tao K, et al. Lithium chloride suppresses colorectal cancer cell survival and proliferation through ROS/GSK-3beta/NF-kappaB signaling pathway. Oxid Med Cell Longev. 2014;2014:241864. 10.1155/2014/241864. PMC4070474 25002914

[310] Costabile V, Duraturo F, Delrio P, Rega D, Pace U, Liccardo R, et al. Lithium chloride induces mesenchymal-to-epithelial reverting transition in primary colon cancer cell cultures. Int J Oncol. 2015;46(5):1913–23. 10.3892/ijo.2015.2911. PMC4383027 25738332

[311] Snitow ME, Bhansali RS, Klein PS. Lithium and therapeutic targeting of GSK-3. Cells (Basel). 2021;10(2). 10.3390/cells10020255. PMC7910927 33525562

[312] Zhou BP, Deng J, Xia W, Xu J, Li YM, Gunduz M, et al. Dual regulation of Snail by GSK-3beta-mediated phosphorylation in control of epithelial-mesenchymal transition. Nat Cell Biol. 2004;6(10):931–40. 10.1038/ncb1173. 15448698

[313] McCubrey JA, Fitzgerald TL, Yang LV, Lertpiriyapong K, Steelman LS, Abrams SL, et al. Roles of GSK-3 and microRNAs on epithelial mesenchymal transition and cancer stem cells. Oncotarget. 2017;8(8):14221–50. 10.18632/oncotarget.13991. PMC5355173 27999207

[314] Lamouille S, Xu J, Derynck R. Molecular mechanisms of epithelial-mesenchymal transition. Nat Rev Mol Cell Biol. 2014;15(3):178–96. 10.1038/nrm3758. PMC4240281 24556840

[315] Wendt MK, Allington TM, Schiemann WP. Mechanisms of the epithelial-mesenchymal transition by TGF-beta. Future Oncol. 2009;5(8):1145–68. 10.2217/fon.09.90. PMC2858056 19852727

[316] Piao S, Lee SH, Kim H, Yum S, Stamos JL, Xu Y, et al. Direct inhibition of GSK3beta by the phosphorylated cytoplasmic domain of LRP6 in Wnt/beta-catenin signaling. PLoS ONE. 2008;3(12):e4046. 10.1371/journal.pone.0004046. PMC2603313 19107203

[317] Jung YS, Park JI. Wnt signaling in cancer: therapeutic targeting of Wnt signaling beyond beta-catenin and the destruction complex. Exp Mol Med. 2020;52(2):183–91. 10.1038/s12276-020-0380-6. PMC7062731 32037398

[318] Lee S, Choi EJ, Cho EJ, Lee YB, Lee JH, Yu SJ, et al. Inhibition of PI3K/Akt signaling suppresses epithelial-to-mesenchymal transition in hepatocellular carcinoma through the Snail/GSK-3/beta-catenin pathway. Clin Mol Hepatol. 2020;26(4):529–39. 10.3350/cmh.2019.0056n. PMC7641573 32829570

[319] Moghbeli M. PI3K/AKT pathway as a pivotal regulator of epithelial-mesenchymal transition in lung tumor cells. Cancer Cell Int. 2024;24(1):165. 10.1186/s12935-024-03357-7. PMC11084110 38730433

[320] Peng Y, Croce CM. The role of MicroRNAs in human cancer. Signal Transduct Target Ther. 2016;1:15004. 10.1038/sigtrans.2015.4. PMC5661652 29263891

[321] Chen Y, Wang J, Wang D, Kang T, Du J, Yan Z, et al. TNNT1, negatively regulated by miR-873, promotes the progression of colorectal cancer. J Gene Med. 2020;22(2):e3152. 10.1002/jgm.3152. PMC7027576 31830337

[322] Zhu T, Zhou P, Yang L, Fang X, Zhi X. Troponin T1 silencing inhibits paclitaxel resistance and the development of breast cancer via suppressing rat sarcoma virus/rapidly accelerated fibrosarcoma 1 pathway. Environ Toxicol. 2024;39(4):2064–76. 10.1002/tox.24084. 38095131

[323] Jing L, Feng L, Zhou Z, Shi S, Deng R, Wang Z, et al. TNNT2 as a potential biomarker for the progression and prognosis of colorectal cancer. Oncol Rep. 2020;44(2):628–36. 10.3892/or.2020.7637. PMC7336514 32627044

[324] Sternberg M, Pasini E, Chen-Scarabelli C, Corsetti G, Patel H, Linardi D, et al. Elevated cardiac troponin in clinical scenarios beyond obstructive coronary artery disease. Med Sci Monit. 2019;25:7115–25. 10.12659/MSM.915830. PMC6774266 31542787

[325] Savic L, Stankovic S, Antonijevic N, Matic D, Lasica R, Krljanac G, et al. Elevated cardiac troponin in non-cardiac conditions unrelated to acute myocardial infarction. Int J Mol Sci. 2025;26(23). 10.3390/ijms262311655. PMC12692183 41373801

[326] Giannitsis E, Katus HA. Cardiac troponin level elevations not related to acute coronary syndromes. Nat Rev Cardiol. 2013;10(11):623–34. 10.1038/nrcardio.2013.129. 23979214

[327] Blich M, Sebbag A, Attias J, Aronson D, Markiewicz W. Cardiac troponin I elevation in hospitalized patients without acute coronary syndromes. Am J Cardiol. 2008;101(10):1384–8. 10.1016/j.amjcard.2008.01.011. 18471446

[328] Scheitz JF, Stengl H, Nolte CH, Landmesser U, Endres M. Neurological update: use of cardiac troponin in patients with stroke. J Neurol. 2021;268(6):2284–92. 10.1007/s00415-020-10349-w. PMC8179917 33372247

[329] Murphy D, Banerjee D. Cardiac troponins in kidney disease. Eur Cardiol. 2025;20:e22. 10.15420/ecr.2024.51. PMC12400178 40901487

[330] Shvilkina T, Shapiro N. Sepsis-Induced myocardial dysfunction: heterogeneity of functional effects and clinical significance. Front Cardiovasc Med. 2023;10:1200441. 10.3389/fcvm.2023.1200441. PMC10375025 37522079

[331] Tanindi A, Cemri M. Troponin elevation in conditions other than acute coronary syndromes. Vasc Health Risk Manag. 2011;7:597–603. 10.2147/VHRM.S24509. PMC3212425 22102783

[332] McEvoy JW, Chen Y, Nambi V, Ballantyne CM, Sharrett AR, Appel LJ, et al. High-sensitivity cardiac troponin T and risk of hypertension. Circulation. 2015;132(9):825–33. 10.1161/CIRCULATIONAHA.114.014364. PMC4558242 26152706

[333] Shahid MH, Ali N, Ratnala M, Ishaque R, Gundala Raja H, Rahbani R, et al. Prognostic value of high-sensitivity troponin in predicting long-term cardiovascular outcomes in patients with type 2 diabetes: a systematic review. Cureus. 2025;17(5):e83925. 10.7759/cureus.83925. PMC12152219 40502917

[334] Pavasini R, d’Ascenzo F, Campo G, Biscaglia S, Ferri A, Contoli M, et al. Cardiac troponin elevation predicts all-cause mortality in patients with acute exacerbation of chronic obstructive pulmonary disease: Systematic review and meta-analysis. Int J Cardiol. 2015;191:187–93. 10.1016/j.ijcard.2015.05.006. 25965630

[335] Elmenawi KA, Anil V, Gosal H, Kaur H, Chakwop Ngassa H, Mohammed L. The importance of measuring troponin in chronic obstructive pulmonary disease exacerbations: a systematic review. Cureus. 2021;13(8):e17451. 10.7759/cureus.17451. PMC8475747 34603857

[336] Hinton J, Mariathas M, Grocott MP, Curzen N. High sensitivity troponin measurement in critical care: flattering to deceive or “never means nothing”? J Intensive Care Soc. 2020;21(3):232–40. 10.1177/1751143719870095. PMC7401433 32782463

[337] Lazar DR, Lazar FL, Homorodean C, Cainap C, Focsan M, Cainap S, et al. High-sensitivity troponin: a review on characteristics, assessment, and clinical implications. Dis Markers. 2022;2022:9713326. 10.1155/2022/9713326. PMC8965602 35371340

[338] de Lemos JA. Increasingly sensitive assays for cardiac troponins: a review. JAMA. 2013;309(21):2262–9. 10.1001/jama.2013.5809. 23736735

[339] Kociol RD, Pang PS, Gheorghiade M, Fonarow GC, O’Connor CM, Felker GM. Troponin elevation in heart failure prevalence, mechanisms, and clinical implications. J Am Coll Cardiol. 2010;56(14):1071–8. 10.1016/j.jacc.2010.06.016. 20863950

[340] Chaulin AM. The metabolic pathway of cardiac troponins release: mechanisms and diagnostic role. Cardiol Res. 2022;13(4):190–205. 10.14740/cr1351. PMC9451590 36128422

[341] Ahmed AN, Blonde K, Hackam D, Iansavichene A, Mrkobrada M. Prognostic significance of elevated troponin in non-cardiac hospitalized patients: a systematic review and meta-analysis. Ann Med. 2014;46(8):653–63. 10.3109/07853890.2014.959558. 25307362

[342] Khan NA, Hemmelgarn BR, Tonelli M, Thompson CR, Levin A. Prognostic value of troponin T and I among asymptomatic patients with end-stage renal disease: a meta-analysis. Circulation. 2005;112(20):3088–96. 10.1161/CIRCULATIONAHA.105.560128. 16286604

[343] Michos ED, Wilson LM, Yeh HC, Berger Z, Suarez-Cuervo C, Stacy SR, et al. Prognostic value of cardiac troponin in patients with chronic kidney disease without suspected acute coronary syndrome: a systematic review and meta-analysis. Ann Intern Med. 2014;161(7):491–501. 10.7326/M14-0743. 25111499

[344] El-Menyar A, Sathian B, Al-Thani H. Elevated serum cardiac troponin and mortality in acute pulmonary embolism: systematic review and meta-analysis. Respir Med. 2019;157:26–35. 10.1016/j.rmed.2019.08.011. 31476570

[345] McGowan JV, Chung R, Maulik A, Piotrowska I, Walker JM, Yellon DM. Anthracycline chemotherapy and cardiotoxicity. Cardiovasc Drugs Ther. 2017;31(1):63–75. 10.1007/s10557-016-6711-0. PMC5346598 28185035

[346] Dempsey N, Rosenthal A, Dabas N, Kropotova Y, Lippman M, Bishopric NH. Trastuzumab-induced cardiotoxicity: a review of clinical risk factors, pharmacologic prevention, and cardiotoxicity of other HER2-directed therapies. Breast Cancer Res Treat. 2021;188(1):21–36. 10.1007/s10549-021-06280-x. 34115243

[347] Copeland-Halperin RS, Liu JE, Yu AF. Cardiotoxicity of HER2-targeted therapies. Curr Opin Cardiol. 2019;34(4):451–8. 10.1097/HCO.0000000000000637. PMC7313632 31082851

[348] Nielsen DL, Juhl CB, Nielsen OH, Chen IM, Herrmann J. Immune checkpoint inhibitor-induced cardiotoxicity: a systematic review and meta-analysis. JAMA Oncol. 2024;10(10):1390–9. 10.1001/jamaoncol.2024.3065. PMC11342217 39172480

[349] van den Berg PF, Bracun V, Noordman M, van der Meer P, Shi C, Oosting SF, et al. Elevations of cardiac troponin in patients receiving immune checkpoint inhibitors: data from a prospective study. JACC Adv. 2024;3(12):101375. 10.1016/j.jacadv.2024.101375. PMC11584941 39583866

[350] Cheng E, Ivanovic M, Chan A, Xu S, Franquiz M, Lee C, et al. Cardiac troponin screening and clinical outcomes in patients receiving immunotherapy. JACC CardioOncol. 2025;7(6):708–21. 10.1016/j.jaccao.2025.06.009. PMC12790047 40810717

[351] Camilli M, Cipolla CM, Dent S, Minotti G, Cardinale DM. Anthracycline cardiotoxicity in adult cancer patients: JACC: cardiooncology state-of-the-art review. JACC CardioOncol. 2024;6(5):655–77. 10.1016/j.jaccao.2024.07.016. PMC11520218 39479333

[352] Upshaw JN, Parson SK, Buchsbaum RJ, Schlam I, Ruddy KJ, Durani U, et al. Dexrazoxane to prevent cardiotoxicity in adults treated with anthracyclines: JACC: cardiooncology controversies in cardio-oncology. JACC CardioOncol. 2024;6(2):322–4. 10.1016/j.jaccao.2024.02.004. PMC11103024 38773999

[353] Kubes J, Karabanovich G, Cong ATQ, Melnikova I, Lencova O, Kollarova P, et al. Topobexin targets the Topoisomerase II ATPase domain for beta isoform-selective inhibition and anthracycline cardioprotection. Nat Commun. 2025;16(1):4928. 10.1038/s41467-025-60167-9. PMC12116762 40425539

[354] Diaz-Anton B, Madurga R, Zorita B, Wasniewski S, Moreno-Arciniegas A, Lopez-Melgar B, et al. Early detection of anthracycline- and trastuzumab-induced cardiotoxicity: value and optimal timing of serum biomarkers and echocardiographic parameters. ESC Heart Fail. 2022;9(2):1127–37. 10.1002/ehf2.13782. PMC8934964 35106939

[355] Sorodoc V, Sirbu O, Lionte C, Haliga RE, Stoica A, Ceasovschih A, et al. The value of troponin as a biomarker of chemotherapy-induced cardiotoxicity. Life (Basel). 2022;12(8). 10.3390/life12081183. PMC9410123 36013362

[356] Ferreira de Souza T, Quinaglia ACST, Osorio Costa F, Shah R, Neilan TG, Velloso L, et al. Anthracycline therapy is associated with cardiomyocyte atrophy and preclinical manifestations of heart disease. JACC Cardiovasc Imaging. 2018;11(8):1045–55. 10.1016/j.jcmg.2018.05.012. PMC6196358 30092965

[357] Demissei BG, Hubbard RA, Zhang L, Smith AM, Sheline K, McDonald C, et al. Changes in cardiovascular biomarkers with breast cancer therapy and associations with cardiac dysfunction. J Am Heart Assoc. 2020;9(2):e014708. 10.1161/JAHA.119.014708. PMC7033834 31959034

[358] Jin G, Wang K, Zhao Y, Yuan S, He Z, Zhang J. Targeting histone deacetylases for heart diseases. Bioorg Chem. 2023;138:106601. 10.1016/j.bioorg.2023.106601. 37224740

[359] Lu J, Qian S, Sun Z. Targeting histone deacetylase in cardiac diseases. Front Physiol. 2024;15:1405569. 10.3389/fphys.2024.1405569. PMC11232433 38983721

[360] He X, Zhang H, Zhang Y, Ye Y, Wang S, Bai R, et al. Drug discovery of histone lysine demethylases (KDMs) inhibitors (progress from 2018 to present). Eur J Med Chem. 2022;231:114143. 10.1016/j.ejmech.2022.114143. 35101649

[361] Gold S, Shilatifard A. Epigenetic therapies targeting histone lysine methylation: complex mechanisms and clinical challenges. J Clin Invest. 2024;134(20). 10.1172/JCI183391. PMC11473148 39403928

[362] Kim GE, Kass DA. Cardiac phosphodiesterases and their modulation for treating heart disease. Handb Exp Pharmacol. 2017;243:249–69. 10.1007/164_2016_82. PMC5665023 27787716

[363] Kamel R, Leroy J, Vandecasteele G, Fischmeister R. Cyclic nucleotide phosphodiesterases as therapeutic targets in cardiac hypertrophy and heart failure. Nat Rev Cardiol. 2023;20(2):90–108. 10.1038/s41569-022-00756-z. 36050457

[364] Yan D, Malyavko A, Wang Q, Ostrikov KK, Sherman JH, Keidar M. Multi-modal biological destruction by cold atmospheric plasma: capability and mechanism. Biomedicines. 2021;9(9). 10.3390/biomedicines9091259. PMC8465976 34572443

[365] Talukdar P, Gohain RB, Biswas S, Bala A. Cold atmospheric plasma in biomedicine and pharmacology: multidisciplinary perspectives on therapeutic applications. ACS Pharmacol Transl Sci. 2026;9(4):859–79. 10.1021/acsptsci.6c00042. PMC13077500 41988368

[366] Fu D, Lin S, Xu Q, Cao F, Khan I, Xu S, et al. Cold atmospheric plasma as a novel “drug” for cancer therapy. J Control Release. 2025;386:114118. 10.1016/j.jconrel.2025.114118. 40803446

[367] Iuchi K, Morisada Y, Yoshino Y, Himuro T, Saito Y, Murakami T, et al. Cold atmospheric-pressure nitrogen plasma induces the production of reactive nitrogen species and cell death by increasing intracellular calcium in HEK293T cells. Arch Biochem Biophys. 2018;654:136–45. 10.1016/j.abb.2018.07.015. 30026027

[368] Schneider C, Gebhardt L, Arndt S, Karrer S, Zimmermann JL, Fischer MJM, et al. Cold atmospheric plasma causes a calcium influx in melanoma cells triggering CAP-induced senescence. Sci Rep. 2018;8(1):10048. 10.1038/s41598-018-28443-5. PMC6030087 29968804

[369] Dai X, Wang M, Liu Y. A Hiplot-based web service for cold atmospheric plasma high-throughput data integration and analysis on breast cancer. Imeta. 2025;4(4):e70045. 10.1002/imt2.70045. PMC12371264 40860433

[370] Wang P, Zhou R, Zhou R, Feng S, Zhao L, Li W, et al. Epidermal growth factor potentiates EGFR(Y992/1173)-mediated therapeutic response of triple negative breast cancer cells to cold atmospheric plasma-activated medium. Redox Biol. 2024;69:102976. 10.1016/j.redox.2023.102976. PMC10746566 38052106

[371] Xiang L, Xu X, Zhang S, Cai D, Dai X. Cold atmospheric plasma conveys selectivity on triple negative breast cancer cells both in vitro and in vivo. Free Radic Biol Med. 2018;124:205–13. 10.1016/j.freeradbiomed.2018.06.001. 29870749

